# A Chemocentric Approach to the Identification of Cancer Targets

**DOI:** 10.1371/journal.pone.0035582

**Published:** 2012-04-25

**Authors:** Beáta Flachner, Zsolt Lörincz, Angelo Carotti, Orazio Nicolotti, Praveena Kuchipudi, Nikita Remez, Ferran Sanz, József Tóvári, Miklós J. Szabó, Béla Bertók, Sándor Cseh, Jordi Mestres, György Dormán

**Affiliations:** 1 TargetEx, Dunakeszi, Hungary; 2 Medicinal Chemistry Department, University of Bari “Aldo Moro”, Bari, Italy; 3 IMIM – Hospital del Mar Research Institute and Universitat Pompeu Fabra, Barcelona, Catalonia, Spain; 4 National Institute of Oncology, Budapest, Hungary; 5 AMRI Hungary Zrt., Budapest, Hungary; University of California Los Angeles, United States of America

## Abstract

A novel chemocentric approach to identifying cancer-relevant targets is introduced. Starting with a large chemical collection, the strategy uses the list of small molecule hits arising from a differential cytotoxicity screening on tumor HCT116 and normal MRC-5 cell lines to identify proteins associated with cancer emerging from a differential virtual target profiling of the most selective compounds detected in both cell lines. It is shown that this smart combination of differential *in vitro* and *in silico* screenings (DIVISS) is capable of detecting a list of proteins that are already well accepted cancer drug targets, while complementing it with additional proteins that, targeted selectively or in combination with others, could lead to synergistic benefits for cancer therapeutics. The complete list of 115 proteins identified as being hit uniquely by compounds showing selective antiproliferative effects for tumor cell lines is provided.

## Introduction

Cancer is a disease of the cell [1]. This rather simple statement implies an enormous complexity when attempting to identify efficacious anticancer agents. One of the major issues associated with anticancer research is that traditional target-directed strategies are confronted with the essentiality of the function of the target in healthy cells. Inevitably, targeting proteins that have essential functions are likely to lead to chemical entities with narrow therapeutic windows and significant toxic effects [2]. An additional challenge is the unstable epigenetic and genetic status of cancer cells, undergoing multiple mutations, gene copy alterations, and chromosomal abnormalities that have a direct impact on the efficacy of anticancer agents at different stages of the disease [3]. All these aspects make cancer drug discovery extremely difficult and have led to poor clinical approval success rates compared to other therapeutic areas [2].

The advent of high-throughput cell-based cytotoxicity assays opened new perspectives for anticancer discovery [4]. The implementation of differential cytotoxicity screens marked the departure from small molecule screens on preconceived individual protein targets and allowed the identification of small molecules potentially acting through a richness of mechanisms of action [5], while showing at the same time selective antiproliferative effects in cancer cells compared to healthy cells [6]. However, as recently pointed out [1], for those cell-based strategies to have a true impact in cancer drug discovery, means to uncover the target profile of bioactive small molecules in antiproliferative or toxicity assays are absolutely necessary. In this respect, extensive proteomic profiling is often applied subsequently to identify differentially expressed proteins in cancer cell lines that may explain the biological effect of small molecule hits [7], [8]. However, profiling the cellular activities of molecular libraries is both technically and logistically a laborious task [9] and thus, alternative approaches for fast and efficient profiling of hundreds of compounds on thousands of proteins are required.

In recent years, the availability of an increasing amount of protein-ligand interaction data in the public domain has promoted the development of ligand-based computational methods aiming at predicting the affinity profile of small molecules across multiple targets [10]. An early application of these initiatives was the prediction of the biological activity spectrum of all small molecules contained in the National Cancer Institute database [11]. Lately, virtual target profiling was successfully used to identify new targets for known drugs [12], to predict the mechanism of action of antimalarials discovered in a high-throughput cell-based screen [13], and to suggest the targets against which selected compounds from a chemical library should be tested, leading to the identification of novel antagonists for all four members of the adenosine receptor family [14]. Given the current levels of performance achieved, in terms of sensitivity and specificity, against experimentally-determined complete ligand-protein interaction matrices [15], these methods are emerging as a true fast and efficient alternative to the more laborious proteomic profiling.

The integration of differential cytotoxicity screening and virtual target profiling for the identification of cancer-relevant targets was put into practice within the context of CancerGrid, a European Commission project under Framework Programme 6 [16]. Details on the approach followed and the results achieved are discussed in the following sections.

## Results

**Figure 1:**
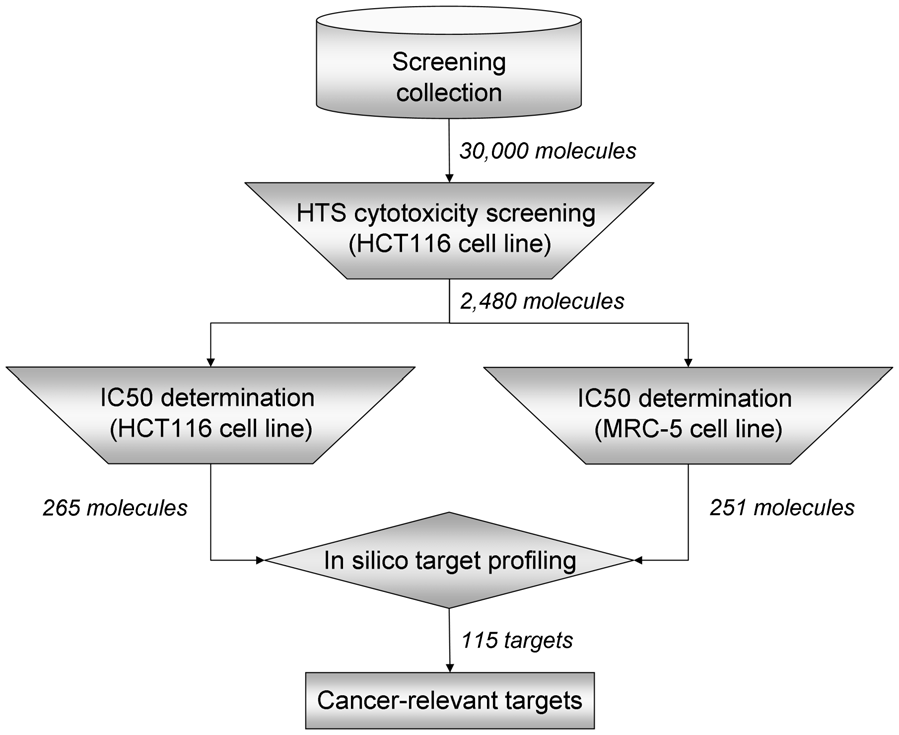
Schematic flowchart of the DIVISS approach applied in this work leading to the identification of 115 proteins of potential relevance to cancer.

For the sake of clarity, a summary scheme of the overall differential *in vitro* and *in silico* screening (DIVISS) process followed in this work is depicted in Figure 1. Starting with a chemical collection of 30,000 compounds, differential cytotoxicity screening resulted in the identification of two sets of small molecule hits showing selective antiproliferative effects for tumor and healthy cells, respectively, which by virtual target profiling led ultimately to the identification of a list of 115 proteins of potential relevance to cancer. Details of the results obtained at each stage of this novel chemocentric approach to cancer target identification are provided next.

### High-throughput cytotoxicity screening

**Figure 2:**
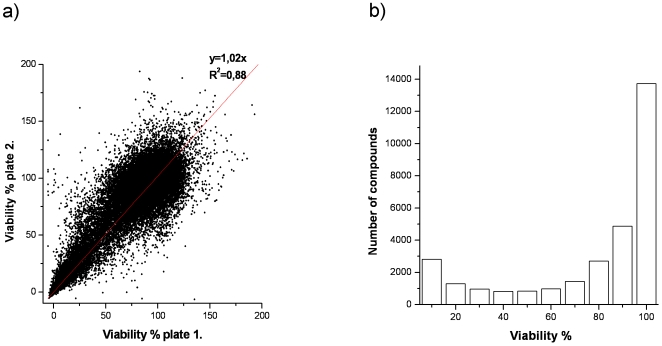
**a) Correlation of two independent viability values determined for the same compound and b) distribution of viability values for the chemical library of 30,000 compounds.**

A cell-based cytotoxicity screening campaign was performed on a chemical collection composed of 30,000 diverse molecules selected mainly from the entire AMRI catalogue [17]. Single point screening of these compounds at 50 µM concentration was completed in duplicate on a colon cancer HCT116 cell line. The correlation of the two independent viability values determined for each compound is depicted in Figure 2a. An average Z′ factor of 0.58 was derived from analysis of these duplicate data, which is indicative of the quality of the assay and the data obtained. The distribution of the number of compounds resulting in different average percentages of cell viability is provided in Figure 2b. As can be observed, almost 50% of the compounds had basically no effect on the viability of the HCT116 cells. But most interestingly, over 13% of the compounds showed remarkable toxic effects on HCT116 cells, with viability values of 20% or lower. This cytotoxic set of 4,158 compounds was selected for a follow-up dose-response screening.

### Differential cytotoxicity dose-response screening

To optimise our capacity of dose-response screening, a diverse set of 2,000 molecules was first selected from the 4,158 cytotoxic compounds identified in the previous high-throughput screening campaign [18]. Dose-response curves on both tumor HCT116 and normal MRC-5 cells were determined in duplicate for these 2,000 compounds. To identify those small molecules that have levels of toxicity on tumor cells significantly higher than those observed on healthy cells, the ratio between the IC_50_ values obtained in MRC-5 cells, IC_50_ (MRC-5), and those obtained in HCT116 cells, IC_50_ (HCT116), was derived for each compound. A total of 230 compounds were identified to be 5 times or more cytotoxic in tumor cells than in healthy cells (IC_50_ MRC-5/IC_50_ HCT116≥5). A chemotype clustering analysis [19] was then performed on this first set of 2,000 compounds for which dose-response data was produced. A cytotoxicity enrichment score was then assigned to each chemotype cluster based on its relative presence in the set of 230 compounds showing most selective antiproliferative effects on tumor cells. Those chemotypes having higher than 20% hit rate were selected and used to recover compounds from the remaining 2,158 for which only single-point measurements were available. This bias towards selective tumor cytotoxic chemotypes led to the identification of 150 compounds that were complemented with an additional set of 330 compounds added on the basis of diversity criteria [18]. Dose-response curves on both cell lines were obtained in duplicate for these 480 compounds, from which an additional set of 35 compounds was identified as having cytotoxic selectivity for tumor cells relative to healthy cells. Altogether, 2,480 compounds went through differential cytotoxicity dose-response *in vitro* screening, leading to the identification of 265 compounds with selective cytotoxicity for tumor cells (Figure 1). Overall, 119,520 cytotoxicity data points were generated, 60,000 from the primary cytotoxicity screenings on HCT116 cells (30,000 compounds in duplicate) and 59,520 from the dose-response screenings (2,480 compounds at 6 concentrations in duplicate on two cell lines), which represents a significant screening effort.

**Figure 3:**
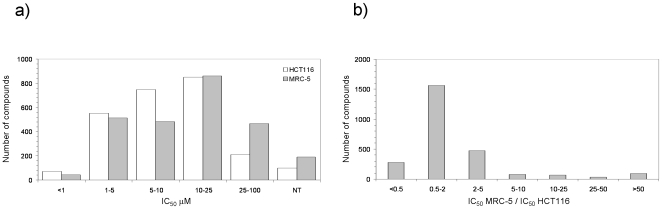
**a) Distribution of the cytotoxicity (IC50 values) of the selected compounds on HCT116 and MRC5 cells and b) distribution of the selective cytotoxicity against HCT116. NT means “non toxic”.**

The distributions of the resulting average IC_50_ values for all 2,480 compounds on tumor HCT116 and normal MRC-5 cells are illustrated in Figure 3a. The fact that most screened compounds have determined IC_50_ values below 25 µM is a good indication of the validity of the first screening. In this respect, just over 12% of the compounds for tumor cells, compared to the almost 26% for normal cells, gave an IC_50_ value above 25 µM, whereas 25% and 22% of the compounds screened on tumor and normal cells, respectively, returned an IC_50_ value below 5 µM. The final distribution of the cytotoxicity ratios per compound is provided in Figure 3b, where large values are associated to promising compounds having some degree of selective cytotoxicity for tumor cells relative to healthy cells. As can be observed, the vast majority of compounds (over 60%) returned cytotoxicity ratios between 0.5 and 2 meaning that they were basically unselective between tumor and healthy cells. But most interestingly, 711 compounds (29%) were found to be 2 times or more cytotoxic in tumor cells than in healthy cells, with 265 of them showing cytotoxicity ratios above 5. In contrast, 277 compounds (11%) were found to be 2 times or more cytotoxic in healthy cells than in tumor cells, with 251 of them having cytotoxicity ratios below 0.2. These two sets of 265 and 251 compounds (Figures S1 and S2) showing selective antiproliferative effects for tumor and normal cells, respectively, will be carried over to the next phase of virtual target profiling (Figure 1). A similarity analysis (Figure S3) highlighted the diversity of chemical structures within each set but also between the two sets, a point worth stressing in support of phenotypic screening approaches over target-directed strategies for complex diseases.

### Virtual target profiling

Each one of the two cell-line selective compound sets was processed *in silico* against the 4,643 ligand-based protein models derived from publicly available resources [20]–[28] using a validated similarity-based approach described earlier [14], [15]. With regards to the 265 selective tumor cytotoxic compounds, at least one target interaction was predicted for 173 of them (65%), reflecting that the chemical space defined by the set of tumor selective compounds was decently covered by small molecules present in public chemogenomic databases. For these compounds, a total of 2,356 molecule-protein interactions were predicted. Of those, 818 interactions between 139 molecules and 229 proteins were predicted to have activities of 1 µM or better (pAct≥6), meaning that on average each tumor selective compound was expected to potentially interact with 6 targets. In comparison, at least one target interaction was predicted for 117 of the 251 selective cytotoxic compounds on normal cells (47%), meaning that 53% of those compounds was found to be outside the applicability domain defined by small molecules in public chemogenomic databases [15]. For these compounds, a total of 1,023 molecule-protein interactions were predicted. Of those, 463 interactions between 84 molecules and 160 proteins were predicted to have activities of 1 µM or better (pAct≥6), resulting in an average number of 5 interacting proteins per compound.

**Figure 4:**
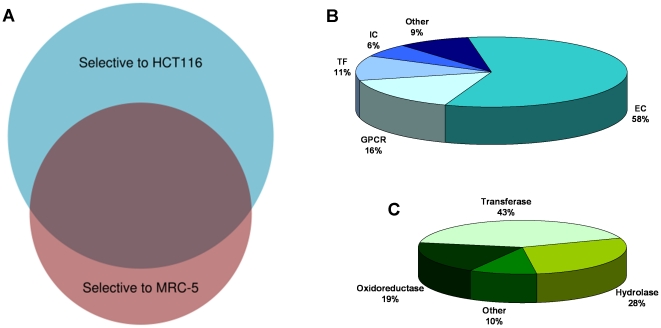
**a) Venn diagram of the protein targets predicted for the selective cytotoxic compounds to HCT116 and MRC-5 cell lines; b) distribution across protein families of the 115 targets predicted to interact uniquely with selective cytotoxic compounds to tumor cells; and c) distribution across enzyme classes of the 67 enzymes present in the list of 115 putative cancer targets.**

A comparative analysis of the predicted interactions from the two cell-line selective compound sets allows gaining a better insight on the proteins likely to be differentially relevant for tumor cell lines. The results are illustrated in the Venn diagram depicted in Figure 4a, which schematically shows the degree of overlap and uniqueness between the two target lists. In this respect, it was found that up to 114 proteins were predicted to be hit at least once by some compound in either set, with the list of proteins being mainly composed by G protein-coupled receptors (45%) and enzymes (37%). In contrast, only 46 proteins were found to be solely hit by compounds with selective cytotoxicity for healthy cells, with a distribution among protein families very similar to the one obtained previously for the list of shared proteins (41% of G protein-coupled receptors and 37% of enzymes). But most interestingly, a list of 115 proteins hit uniquely by compounds with selective cytotoxicity for tumor cells was identified (Table S1). Analysis of its composition among the main protein families of therapeutic relevance reveals a clearly differentiated signature from the other two lists of proteins. As shown in Figure 4b, the list is mainly composed of enzymes (58%) and the presence of G protein-coupled receptors has been reduced significantly (16%). To complement this picture, Figure 4c provides the class distribution of the 67 enzymes found in this list. A clear bias towards transferases (43%) is observed, very much in agreement with the importance conferred to kinases as therapeutic targets for cancer [29], [30].

### Proof of concept

It may not escape the scrutinous eye of the cancer researcher that within the list of 115 potential tumor selective proteins (Table S1) there are two widely recognized anticancer targets, namely, histone deacetylases (HDACs) and heat shock protein 90-alpha (HSP90), both of which known to be expressed in colon cancer HCT116 cell lines [7], [8] and to confer tumor selectivity upon small molecule inhibition [31], [32]. Accordingly, in an attempt to close the cycle of the DIVISS approach presented above, inhibitors of these two targets were used to exemplify at this stage that indeed selective antiproliferative effects can be achieved on the tumor HCT116 and normal MRC-5 cell lines used in this work.

To this end, suberoylanilide hydroxamic acid (SAHA) and 17-(allylamino)-17-demethoxygeldanamycin (17AAG) were selected as representative pan-HDAC and HSP90 inhibitors, respectively. Dose-response curves on both HCT116 and MRC-5 cell lines were determined for the two inhibitors (Figure S4). The results confirmed that both compounds inhibited the proliferation of HCT116 cells in a dose dependent manner, while having little or no effect on MRC-5 cells. In particular, the IC_50_ values of SAHA and 17AAG on HCT116 cells were 0.64 µM and 0.2 µM, respectively, which resulted in 781 and 93 fold selectivity, respectively, relative to the antiproliferative effect on MRC-5 cells. These observations provide confirmation of the ability of the DIVISS approach for identifying cancer-relevant targets.

We checked also whether within the set of 265 compounds showing selective antiproliferative effects for tumor cell lines there was any compound that could have been tested on a range of colon cancer cell lines and for which screening data was also available in the public domain. Much to our surprise, we found experimental data in PubChem [23] for eight compounds that were also present in our tumor selective set (Table S2). Among them, five compounds are reported to have affinity for the amine oxidase flavin-containing B enzyme (MAO-B), a target present in our list of 115 putative cancer-relavant proteins (Table S1). But, most interestingly, one of them, NSC680350 (CID 387030), was reported to have an IC_50_ of 80 nM for MAO-B, in good agreement with our predictions. In addition, it was also tested at multiple human tumor cell lines, including six colon cancer cell lines. Among them, the pGI50 value reported in PubChem for colon HCT116 cell lines (4.64) is, within the variability limits of this type of experiments, in good agreement with the pGI50 value obtained in this work for the same type of cell lines (5.19). The dose-response curve of the cytotoxicity of NSC680350 on HCT116 cell lines in this work and a summary of all colon cancer data found in PubChem for this compound is provided in Figure S5.

## Discussion

Substantiation of the potential relevance to cancer of the list of 115 proteins identified as being targeted solely by tumor selective compounds was performed by two independent perspectives. On the one hand, all 115 proteins were scored on the basis of recently derived oncogene probabilities (OncoScores) and checked for currently available experimental data on the up- and down-regulation in colon cancer samples [33], [34]. On the other hand, we used all drug-target interaction data available from public resources [20]–[28] to rank order all drugs based on the number of known targets within the list of 115 proteins and check for whether cancer was the primary indication among the top ranked. The results provide ample support for the use of the DIVISS approach to identifying cancer-relevant targets.

**Figure 5:**
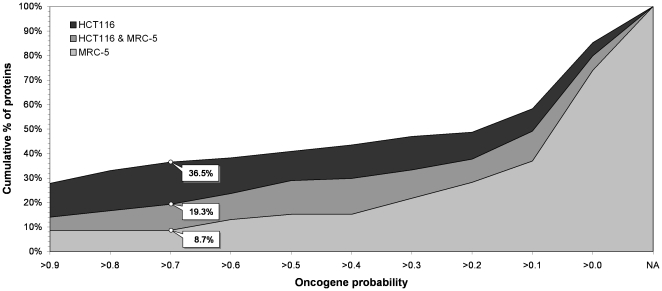
Distribution of oncogene probabilities for the proteins predicted uniquely for compounds selective to HCT116 (black) and MRC-5 (light grey) and the proteins found in both selective sets (dark grey). NA collects all proteins for which oncogene probabilities were not available from CGPrio [34].

The OncoScores for all 115 proteins targeted by tumor selective compounds were obtained from the CGPrio website [34]. To assess whether this list of proteins is enriched with probable oncogenes with respect to other lists of proteins, OncoScores were also calculated for all the 46 proteins targeted by normal selective compounds and the 114 proteins shared by the two sets of cell-line selective compounds. The trends of the cumulative percentage of proteins with OncoScores above a certain probability value found within each list are displayed in Figure 5. As can be observed, it is found that 36.5% of the 115 proteins targeted by tumor selective compounds have an oncogene probability above 0.7 and that, under the same OncoScore cutoff, this percentage is significantly higher than the 8.7% and 19.3% of the 46 proteins targeted by normal selective compounds and the 114 proteins shared by the two sets of compounds, respectively. Having provided evidence that this selection of 115 tumor selective proteins is enriched with putative oncogenes, the IntOGen platform [33] was then used to inspect whether any protein from the list was in addition known to be significantly altered (corrected p-value <0.05) in terms of up- or down-regulation in colon cancer. A total of 29 of those proteins (25%) could indeed be confirmed to be significantly altered in colon cancer, 10 of which having an OncoScore above 0.7. The OncoScores and regulation marks for the whole list of 115 tumor selective proteins are provided Table S1.

The subset of 42 tumor selective proteins with OncoScore higher than 0.7 is provided in Table 1. Not surprisingly, its composition is highly biased by protein kinases (52%), although there is also an important representation (21%) of transcription factors. Of mention is however the fact that a couple of G protein-coupled receptors (GPCRs) are found in this highly probable oncogene subset, namely, the D(1A) dopamine receptor (DRD1) and the sphingosine 1-phosphate receptor 1 (S1PR1). GPCRs have traditionally been regarded as the main targets for diseases of the central nervous system. But most interestingly, the relevance of GPCRs in cancer drug discovery was revisited recently and the potential role of S1PR1 in particular highlighted [35].

**Table 1:** List of 42 proteins with OncoScore >0.7 among the 115 proteins identified by the DIVISS approach.

No.	Protein Name	Gene Name	OncoScore
1	Alpha-type platelet-derived growth factor receptor	PDGFRA **↓**	1.000
2	Androgen receptor	AR	1.000
3	Angiopoietin-1 receptor	TEK	1.000
4	B-Raf proto-oncogene serine/threonine-protein kinase	BRAF	1.000
5	Epidermal growth factor receptor	EGFR	1.000
6	Estrogen receptor	ESR1	1.000
7	FL cytokine receptor	FLT3	1.000
8	Hepatocyte growth factor receptor	MET **↑**	1.000
9	Mast/stem cell growth factor receptor	KIT **↓**	1.000
10	Proto-oncogene tyrosine-protein kinase ABL1	ABL1	1.000
11	Proto-oncogene tyrosine-protein kinase Src	SRC	1.000
12	RAF proto-oncogene serine/threonine-protein kinase	RAF1	1.000
13	Vascular endothelial growth factor receptor 1	FLT1	1.000
14	Vascular endothelial growth factor receptor 3	FLT4	1.000
15	Cell division protein kinase 2	CDK2	0.999
16	Nuclear factor of activated T-cells, cytoplasmic 1	NFATC1	0.999
17	Peptidyl-prolyl cis-trans isomerase FKBP1A	FKBP1A **↓**	0.999
18	Signal transducer and activator of transcription 3	STAT3	0.999
19	Cell division protein kinase 5	CDK5	0.998
20	Estrogen receptor beta	ESR2	0.998
21	Glycogen synthase kinase-3 alpha	GSK3A	0.996
22	Proto-oncogene tyrosine-protein kinase FGR	FGR	0.992
23	Mitogen-activated protein kinase kinase kinase 8	MAP3K8 **↑**	0.984
24	Short transient receptor potential channel 4	TRPC4	0.981
25	Histone deacetylase 4	HDAC4	0.975
26	Mitogen-activated protein kinase 10	MAPK10	0.974
27	TGF-beta receptor type-1	TGFBR1	0.970
28	E3 ubiquitin-protein ligase Mdm2	MDM2 **↑**	0.966
29	Histone deacetylase 7	HDAC7	0.959
30	Peroxisome proliferator-activated receptor gamma	PPARG	0.959
31	Histone deacetylase 9	HDAC9 **↓**	0.953
32	Acyl-CoA desaturase	SCD **↑**	0.940
33	Dual specificity mitogen-activated protein kinase kinase 1	MAP2K1	0.895
34	Histone deacetylase 1	HDAC1	0.895
35	Histone deacetylase 6	HDAC6	0.895
36	D(1A) dopamine receptor	DRD1	0.866
37	Sphingosine 1-phosphate receptor 1	S1PR1	0.863
38	Signal transducer and activator of transcription 1-alpha/beta	STAT1 **↑**	0.824
39	Krueppel-like factor 5	KLF5 **↓**	0.745
40	Poly [ADP-ribose] polymerase 1	PARP1	0.711
41	Phosphatidylinositol-4,5-bisphosphate 3-kinase	PIK3CD	0.708
42	Cyclin-dependent kinase 5 activator 1	CDK5R1	0.701

The OncoScore is the oncogene probability calculated from CGPrio [34].The arrows next to the gene name mark the set of 10 proteins from this list that are known to be significantly altered (corrected p-value <0.05) in terms of up- or down-regulation in colon cancer, as extracted from the IntOGen platform [33].

A close look at the top-20 ranked proteins present in Table 1 reveals that the list contains proteins that may be somewhat unexpected from the viewpoint of its relationship to colorectal cancer. For example, the androgen (AR) and estrogen (both ESR1 and ESR2) nuclear hormone receptors are known to be relevant in prostate and breast cancers, and the alpha-type platelet-derived (PDGFRA) and epidermal (EGFR) growth factor receptors are recognised angiogenesis factors. However, recent studies suggest a role in intestinal carcinogenesis for nuclear receptors in general [36] and growth factor receptors [37], including precisely AR [38], ESR1 [39], ESR2 [40], PDGFRA [41] and EGFR [42]. PDGFRA in particular is also known to be significantly down-regulated in colon cancer [33]. In addition, further evidences exist in the literature of drugs targeting primarily some of those targets and having an effect on the proliferation of human colorectal tumour cell lines, including HCT116 [40], [43]. Among them, raloxifene is a high affinity binder of both ESR1 and ESR2 and has been reported to inhibit HCT116 cell growth in a dose-dependent manner [40] and afatinib is a potent EGFR inhibitor that was recently shown to inhibit the growth of HCT116 cell lines with an IC_50_ value of 1.62 µM [43]. These examples provide ample bibliographical support to the relevance in colon cancer for some of those proteins that would have been otherwise completely overlooked.

It may also surprise that currently recognised cancer targets, such as HSP90, are not present in Table 1. In this particular case, the target is indeed contained in the full list of 115 proteins provided in Table S1 but with a low OncoScore = 0.023. It is thus worth stressing here that CGPrio [34] is a machine learning method based on the differential properties of known cancer genes and on the assumption that genes with similar properties (including sequence conservation, protein domains and interactions, and regulatory data) to known cancer genes are more likely to be involved in cancer. It is used here as a prioritization method, as it has been shown that a large percentage of new cancer genes have high CGPrio probabilities [33], [34], but it doesn't mean that absolutely all cancer genes share these properties, and thus there may well be some *bona fide* cancer targets, such as HSP90, with a low CGPrio probability. In this respect, the low OncoScore obtained for HSP90 means only that, on the basis of current knowledge on cancer genes, HSP90 does not share properties with the rest of cancer genes for which information is available. Taken together, these results emphasize the potential applicability of the DIVISS approach as a complementary strategy to the identification of cancer-relevant targets.

The BioCarta resource [44] was then used to perform an analysis of the main pathways in which these 42 highly probable oncogenes are involved. A total of 131 pathways were retrieved, with 68 of them (52%) having two or more proteins and only 9 (7%) containing five or more proteins. The latter group is composed mainly of signaling pathways. Among them, the MAPKinase signaling pathway contains seven of those probable oncogenes, namely, BRAF, MAP2K1, MAP3K8, MAPK10, RAF1, STAT1, and TGFBR1, and the Erk1/Erk2 MAPK signaling pathway involves six of them, namely, EGFR, MAP2K1, PDGFRA, RAF1, SRC, and STAT3 (see Table 1). The remaining 7 pathways are the Bioactive peptide induced, EGF, and PDGF signaling pathways, the signaling of hepatocyte growth factor receptor, and the ones defining the role of ERBB2 in signal transduction and oncology, the CARM1 and regulation of the estrogen receptor, and the sumoylation by RANBP2 regulates transcriptional repression, all involving 5 of those probable oncogenes (Table 1). The link between some of these pathways and cancer has been already recognised in previous studies [45], [46].

**Figure 6:**
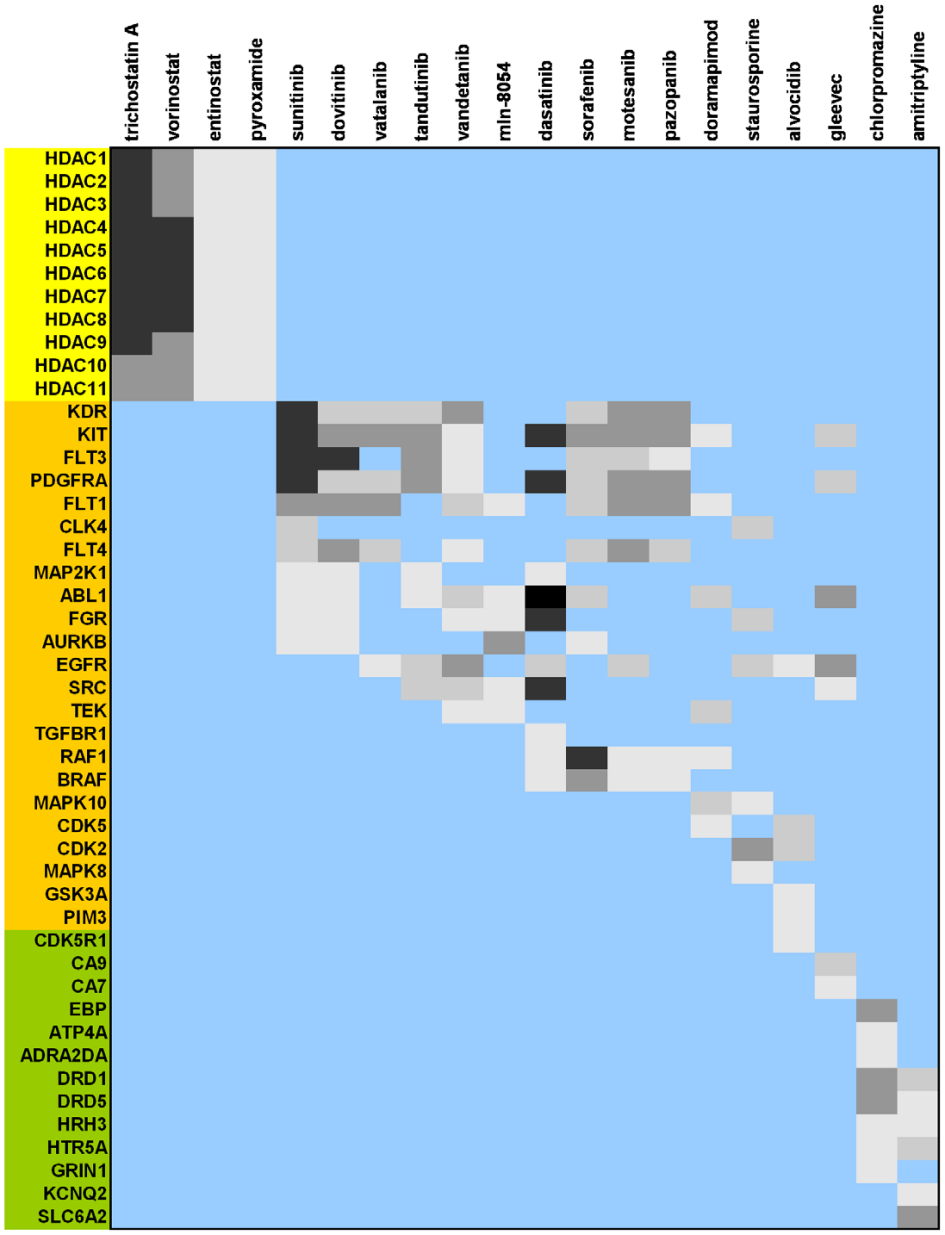
Profiles of experimental affinity data of the 20 drugs, among 4,819, hitting more than 5 targets found solely in tumor selective compounds. Only affinities above 1 µM are considered. Color coding reflects pAffinity ranges: white 6–7; light grey 7–8; dark grey 8–9; black >9. Color codes for targets refer to HDACs (yellow), kinases (orange), and other (green).

In recent years, the amount of publicly available *in vitro* data on the interaction of drugs with multiple proteins has increased dramatically [20]–[28]. Analysis of these data has revealed that most cancer drugs are multitarget agents rather than selective molecules [47]. Accordingly, we took the list of 115 targets hit by selective compounds on HCT116 and performed a search for those drugs that, based on currently available affinity data determined experimentally [20]–[28], would show at least micromolar affinity on the largest number of those targets. Figure 6 collects the results obtained for the 20 drugs having at least micromolar affinity for more than 5 tumor selective proteins. Remarkably, 18 of those drugs have cancer as their primary indication, 4 of which target mainly HDACs, whereas the other 14 have different affinity profiles on a wide range of kinases. The presence of chlorpromazine and amitriptyline in this list, indicated for psychosis and depression, respectively, and targeting mainly GPCRs instead of HDACs or kinases, may come as a surprise at this stage. However, in the line of what was previously mentioned about the new perception of GPCRs in cancer [35], recent reports indicate that chlorpromazine, potentially through its action on multiple tumor selective GPCRs, can change influx properties of membranes and that this property makes it a promising chemosensitizing compound for enhancing the cytotoxic effect of tamoxifen, an antagonist of the estrogen receptor, present also in the list of 115 tumor selective proteins [48]. From a drug perspective, these results provide further support to the relevance for cancer of the 115 proteins identified.

There are two recognisable extensions to the version of the DIVISS approach presented here. The first obvious extension is in the use of other cell lines. In this particular study, HCT116 and MRC-5 cell lines have been taken as models of tumor and healthy cell lines, respectively. However, there are numerous alternative human tumor cell lines that can be used instead and those can in turn be differentially compared to several healthy cell lines as well [49]. Accordingly, differential anticancer screens on each particular combination of tumor and healthy cell lines will in principle lead to different, yet complementary, lists of cancer-relevant targets. The second potential extension is in the coverage of larger chemical spaces, an aspect that is inherent to any screening campaign. The present study focussed on a diverse selection of 30,000 molecules from the AMRI catalogue, currently containing over 240,000 compounds. The size and nature of the chemical library used in the differential cytotoxicity screens essentially determines the number and diversity of small molecule hits identified and they ultimately define the type of targets that, by means of *in silico* target profiling, will be selectively associated to each cell line.

### Conclusions

Cell systems are implicitly robust and selectively acting on one particular target may not be the most efficacious way of modulating or interfering with them as the system may always find ways to compensate for the selective perturbation incorporated. Instead, targeting multiple essential targets in tumor cells may be a more efficient strategy to make more difficult for the cell system to compensate for all perturbations introduced. Indeed, recent evidences indicate that most cancer drugs attain their *in vivo* efficacy through modulation of multiple targets rather than selective interaction on a single target. The big question is then defining the essential protein signature of each cancer type, so it can be thoroughly addressed by novel cancer therapeutic agents [50]. The DIVISS strategy presented here represents a novel chemocentric approach to the identification of cancer-relevant drug targets that complements efficiently other established bioinformatics and functional approaches [51], [52] and thus may contribute to increasing our confidence on potential drug targets [53].

## Materials and Methods

### Screening Library

The CancerGrid consortium had privileged access to the entire chemical catalogue at AMRI [17], currently containing 241,000 compounds and found particularly relevant for drug discovery purposes in a comparative analysis of 23 supplier databases [54]. This relatively vast, diverse and unique chemical space was complemented with a focused set of 1,500 compounds synthesized at the University of Bari. To adjust the number of compounds to our screening capacity, an optimal diversity selection was performed [55]. The final screening collection was limited to a diverse selection of 30,000 compounds, a number that was fitting optimally our capacity for cytotoxicity screening.

### Cell types and culture conditions

A colon adenocarcinoma (HCT116) and normal human lung fibroblast (MRC-5) cell lines were purchased from the American Type Culture Collection (ATCC, Manassas, VA). HCT116 cells were maintained in McCoy's 5a Modified Medium supplemented with 10% FBS, 100 units/ml penicillin, and 100 µg/ml streptomycin at 37°C in a humidified 5% CO_2_ incubator. Subculturing was done using 1∶5 ratio twice a week. MRC-5 cells were maintained in DMEM supplemented with 10% FBS, 100 units/ml penicillin, and 100 µg/ml streptomycin at 37°C in a humidified 5% CO_2_ incubator. Subculturing was done using 1∶3 ratio twice a week. A cell bank was established for the cell lines used during the differential toxicity screening campaign. In all experiments, the doubling time between cancer and normal cells was approximately 24 hours.

### Assay developments and validation

The high-throughput *in vitro* assay for measuring toxicity and antiproliferative effects of small molecules was implemented as described earlier [56]. Cell lines were grown in culture flasks to 90% confluences, then harvested in counted cell density and seeded into 384-well microtiter plates. Test compounds were then added in various concentrations (in 2% DMSO final concentration) and incubated with the cells in CO_2_ incubators at 37°C for 48 h. This incubation period has the advantage of allowing those compounds that are not directly toxic but can block or slow down cell proliferation to have enough time to show their effect. During this period, FBS content of the medium was decreased to avoid the masking effect of FBS on toxicity. Detection of viability is based on the reduction of resazurin (Alamar blue) by living cells, resulting in an increased fluorescent signal [57]. For the transformation of the assay into a high-throughput format, a Beckman Biomek liquid handling system and a Wallac Victor plate reader were used. Protocols needed for handling the 384-well plates were established for both single-concentration screening and dose-response curve determination. A plate map was used for the validation of the assay on 384-well plates, which is suitable for the determination of dose-response curves for 16 compounds at the same time using 6 concentrations in triplicate. This experiment was run in parallel on 5 plates and repeated three times on different days. From the dose-response curves, IC_50_ values were determined and analyzed. To test the reproducibility and robustness of the assay for high-throughput screening, Z′ factors (∼0.72) and S/B ratios (∼10) were determined and the respective plate-to-plate and day-to-day coefficients of variation found to be 5.0%, 2.7%, 1.7% and 5.6%. Based on the established assay protocol, single-point screenings were done at 50 µM compound concentration in duplicate. Likewise, IC_50_ values were obtained from the toxicity dose-response curves from six compound concentrations in duplicate and calculated with Microcal Origin 5.0. Compounds showing selective cytotoxicity for tumor cells relative to healthy cells are identified by large values of the ratio IC_50_(MRC-5)/IC_50_(HCT116), whereas the inverse of this ratio serves to recognize compounds with selective cytotoxicity for healthy cells relative to tumor cells.

### Chemogenomic databases

There are currently several public sources that contain chemical structures with information on the binding or functional activity to protein targets. Those used in the present work include ChEMBLdb [20], PDSP [21], IUPHARdb [22], PubChem [23], DrugBank [24], BindingDB [25], BindingMOAD [26], AffinDB [27], and NRacl [28]. Altogether contain a total of 329,303 unique ligands with 1,505,348 interactions to 4,643 unique proteins. Among them, there are 4,819 small-molecule drugs with 30,875 interactions to 4,120 unique protein targets.

### Affinity predictions and validation

To be processed efficiently, molecular structure information needs to be encoded using some sort of mathematical descriptors. In this work, three types of two-dimensional descriptors were used, namely, SHED, FPD, and PHRAG [58], [59], each one of them characterizing chemical structures with a different degree of fuzziness and thus complementing each other in terms of structural similarity and hopping abilities. For any biological target under study, the ensemble of molecular descriptors capturing the structural and pharmacophoric features of all molecules for which affinity data is publicly available from chemogenomic databases represents a mathematical description of this target from a chemical perspective. On this basis, the affinity of a compound for a given target can be estimated by inverse distance weighting interpolation of the experimental affinities from all neighboring molecules found within a pre-determined applicability domain [15]. Based on the ligand-based target models defined from all the pharmacological data available in chemogenomic databases, each small molecule can be currently processed against 4,643 proteins. The output returns a list of the targets for which affinity is predicted for every query molecule. The method has been successfully validated retrospectively, on its ability to predict the entire experimental interaction matrix between 13 antipsychotic drugs and 34 protein targets [15], but also prospectively, on its capacity to identify the correct targets for all molecules contained in a biologically-orphan chemical library [14] and to correctly anticipate the affinity profile of the drug cyclobenzaprine on a panel of 8 protein targets [60].

### Oncogene expression and probabilities

Expression profiles for proteins in various types of cancer were directly extracted from IntOGen [33]. IntOGen is a framework that currently contains and integrates data from almost 800 independent experiments collecting transcriptomic alterations, genomic gains and losses, and somatic mutation information in different human cancer types. Oncogene probabilities (OncoScores) were calculated with CGPrio [34] using the PC-GS-PD-PI-RD dataset that integrates a set of heterogeneous data accounting for protein conservation (PC), gene structure (GS), protein domains (PD), protein-protein interactions (PI), and regulatory data (RD).

### Small molecule inhibitors

SAHA, a pan-HDAC inhibitor, was kindly provided by Ciro Mercurio (DAC s.r.l., Milan, Italy) with 95% purity and 17AAG, a HSP90 inhibitor, was obtained from Sigma (St Louis, MO) with 95% purity.

## Supporting Information

Figure S1 **Distributions of pairwise similarities using PHRAGS (top) and PFPD (bottom) descriptors between compounds with selective cytotoxicity in HCT116 cell lines (left), MRC-5 cell lines (middle), and HCT116 and MRC-5 cell lines (right).**
(PDF)

Figure S2 **List of chemical structures showing selective cytotoxicity for HCT116 cell lines.**
(PDF)

Figure S3 **List of chemical structures showing selective cytotoxicity for MRC-5 cell lines.**
(PDF)

Figure S4 **Dose-response curves of 17AAG, an HSP90 inhibitor (left), and SAHA, a HDAC inhibitor (right), on the HCT116 and MRC-5 cell lines.**
(PDF)

Figure S5 **Dose-response curve (left) of the cytotoxicity of compound NSC680350 (CID 387030; internally known as MC-309) on HCT116 cell lines (GI50 = 6.4 µM).** Also provided (right) are the pGI50 values of the compound on the two cell lines tested in this work and the six colon cancer cell lines for which data is available in PubChem.
(PDF)

Table S1 **List of all 115 proteins identified by the DIVISS approach as from small molecule hits selective to HCT116 relative to MRC-5.** The OncoScore is the oncogene probability calculated with CGPrio [34]. The arrows next to the gene name mark the set of 29 proteins that are known to be significantly altered (corrected p-value <0.05) in terms of up- or down-regulation in colon cancer, as extracted from the IntOGen platform [33].
(DOC)

Table S2 **List of all interactions available in PubChem for compounds present within the list of 265 cytotoxic selective in HCT116 cell lines.**
(DOC)
