# Supplementary figures and images for: A Chemocentric Approach to the Identification of Cancer Targets
Source: PLoS One. 2012 Apr 25;7(4):e35582. doi: 10.1371/journal.pone.0035582 (PMC3338416; doi:10.1371/journal.pone.0035582)

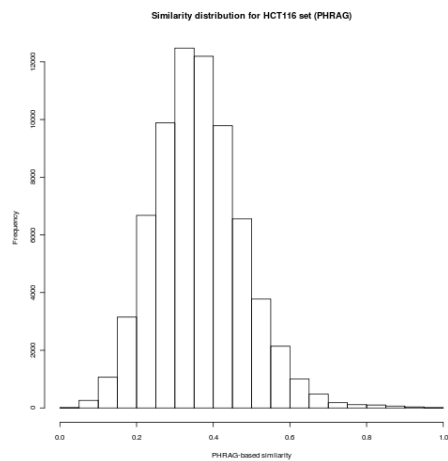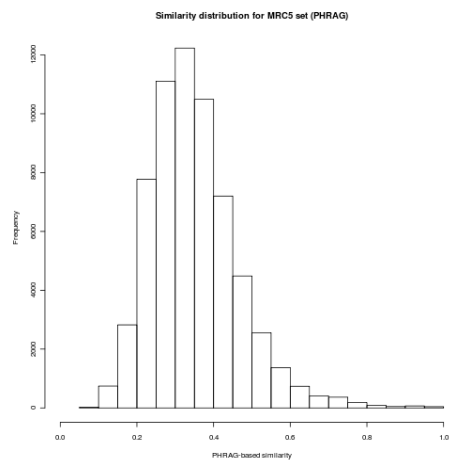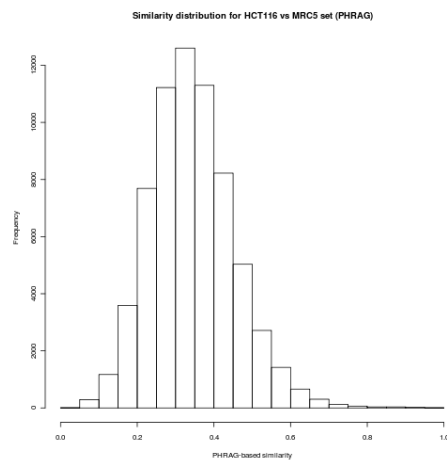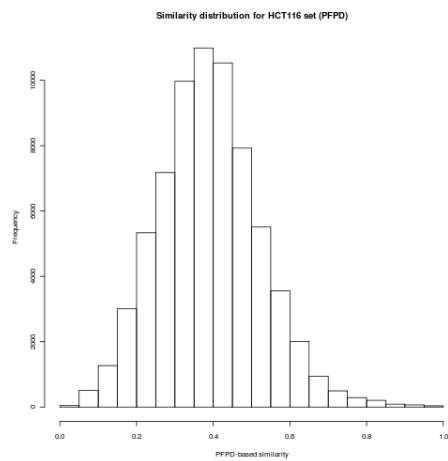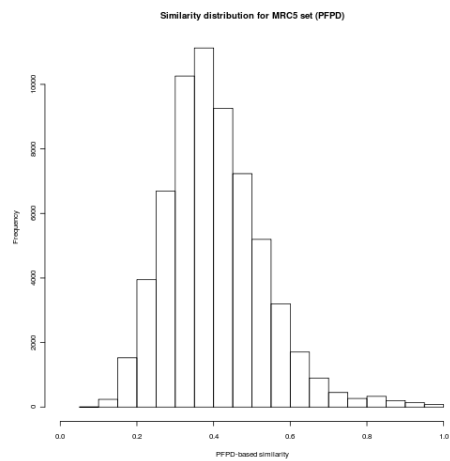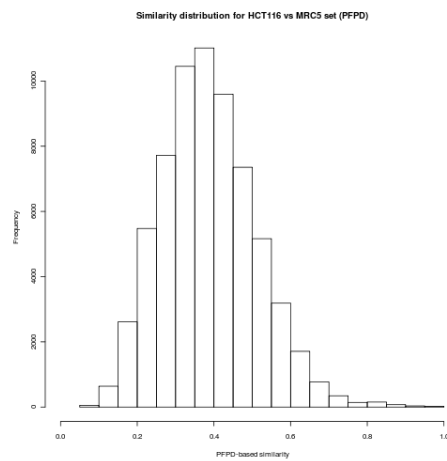

Supplement: Figure S1 — Distributions of pairwise similarities using PHRAGS (top) and PFPD (bottom) descriptors between compounds with selective cytotoxicity in HCT116 cell lines (left), MRC-5 cell lines (middle), and HCT116 and MRC-5 cell lines (right). (PDF) [file pone.0035582.s001.pdf]

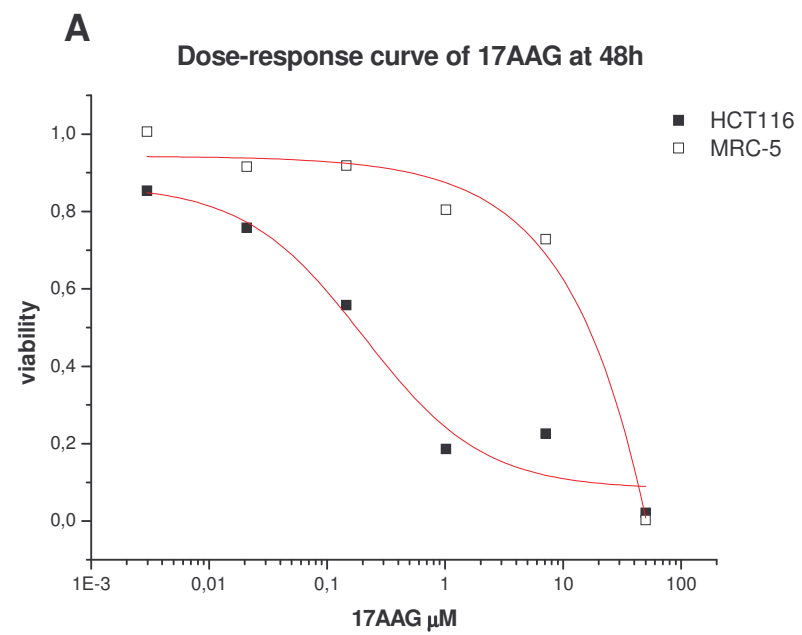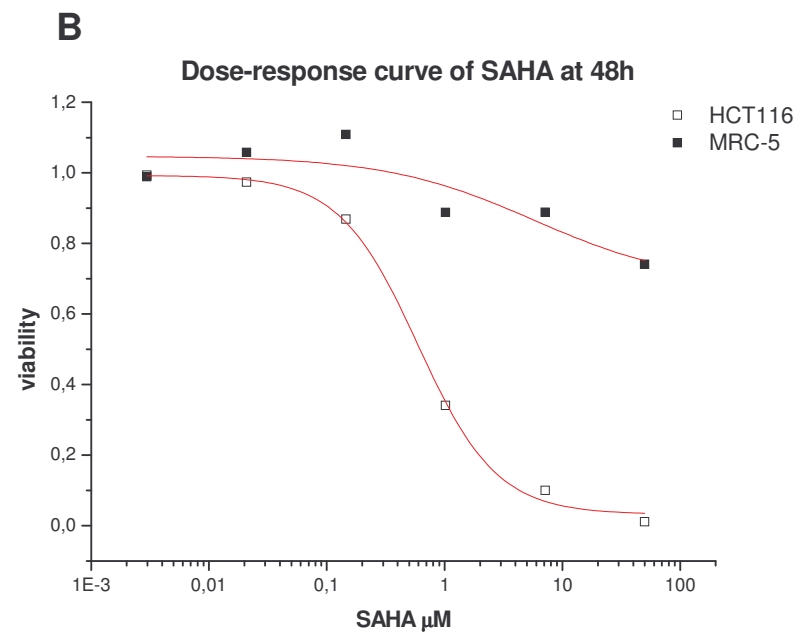

Supplement: Figure S4 — Dose-response curves of 17AAG, an HSP90 inhibitor (left), and SAHA, a HDAC inhibitor (right), on the HCT116 and MRC-5 cell lines. (PDF) [file pone.0035582.s004.pdf]

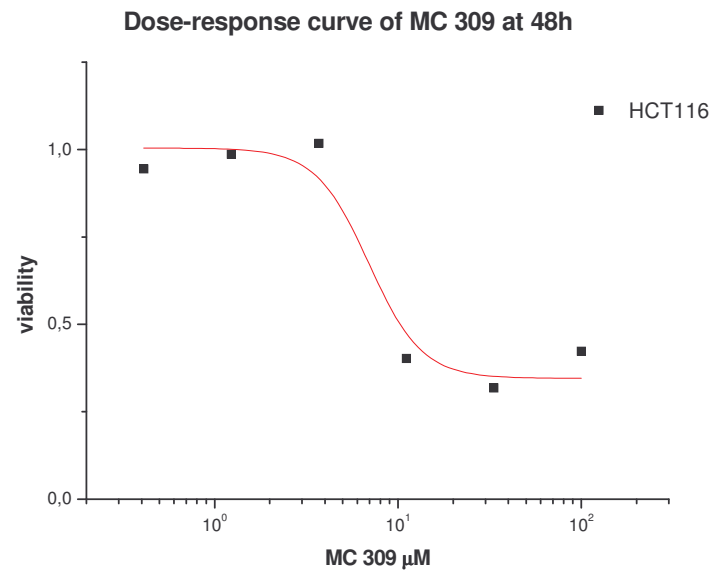

| This Work |       |
|-----------|-------|
| Assay     | pGI50 |
| HCT116    | 5.19  |
| MRC5      | 4.15  |

  

| PubChem      |       |
|--------------|-------|
| Assay (AID)  | pGI50 |
| HCT116 (79)  | 4.64  |
| HT29 (65)    | 5.74  |
| COLO205 (67) | 5.41  |
| HCT15 (71)   | 4.81  |
| KM12 (73)    | 5.10  |
| SW620 (81)   | 4.80  |

Supplement: Figure S5 — Dose-response curve (left) of the cytotoxicity of compound NSC680350 (CID 387030; internally known as MC-309) on HCT116 cell lines (GI50 = 6.4 µM). Also provided (right) are the pGI50 values of the compound on the two cell lines tested in this work and the six colon cancer cell lines for which data is available in PubChem. (PDF) [file pone.0035582.s005.pdf]
