# Supplementary material for: A Chemocentric Approach to the Identification of Cancer Targets
Source: PLoS One. 2012 Apr 25;7(4):e35582. doi: 10.1371/journal.pone.0035582 (PMC3338416; doi:10.1371/journal.pone.0035582)

#1 [1 - 24]

ALB-H03182139

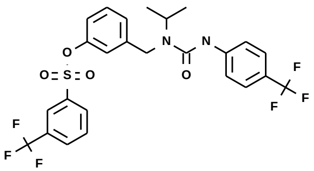

ALB-H10705704

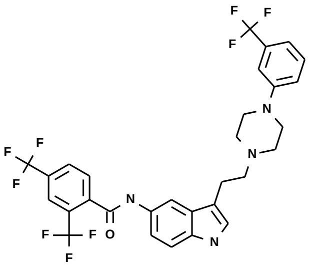

ALB-H03182151

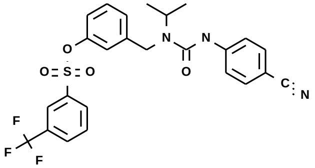

ALB-H10719516

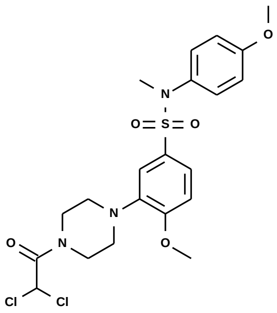

ALB-H10729695

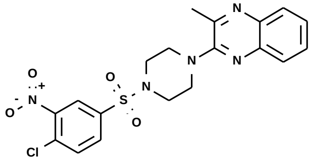

ALB-H10721792

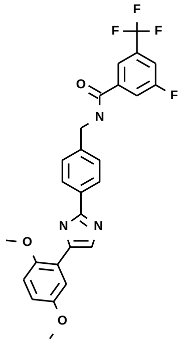

ALB-H09997172

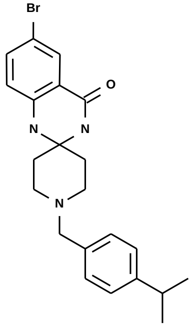

ALB-H00740616

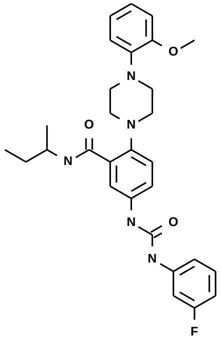

ALB-H04120057

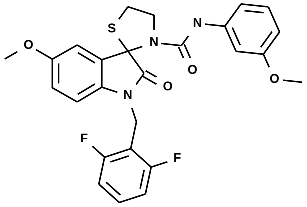

ALB-H10715834

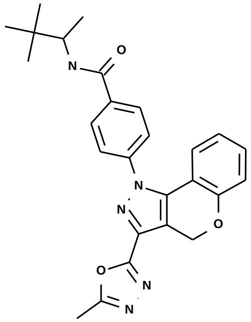

ALB-H01847238

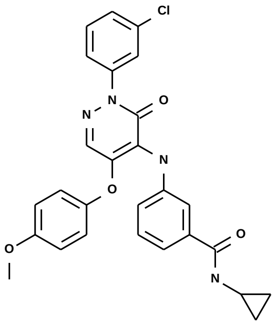

ALB-H00737497

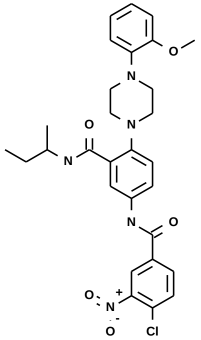

ALB-H00741347

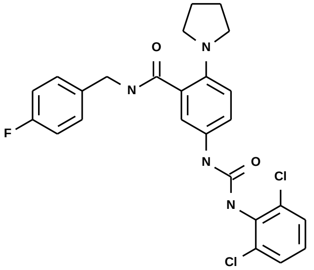

ALB-H01835414

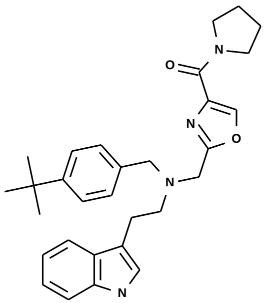

ALB-H02094606

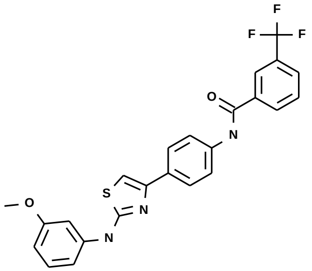

IP_0036

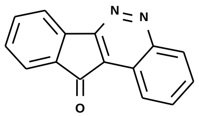

ALB-H01278802

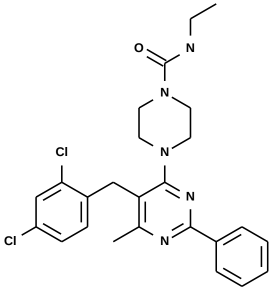

ALB-H09994745

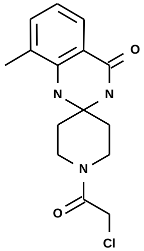

ALB-H09451630

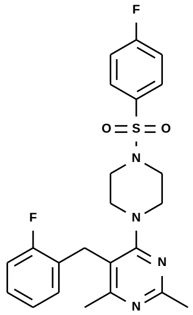

IP_0085

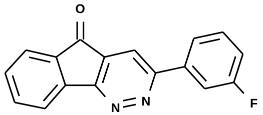

ALB-H10717024

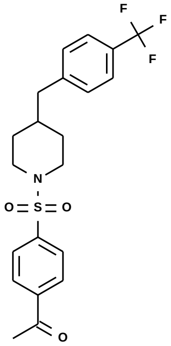

ES_1901

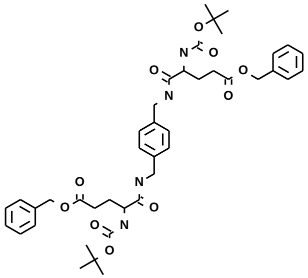

ALB-H10722657

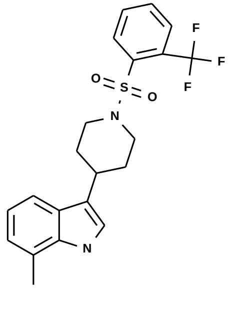

ALB-H00884710

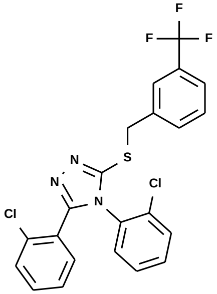

#2 [25 - 48]

ALB-H10724022

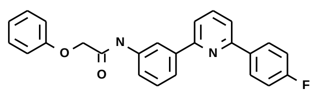

ALB-H03209455

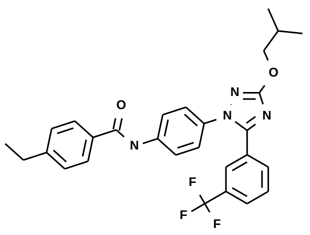

AS_0299

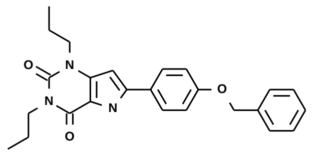

ALB-H10995923

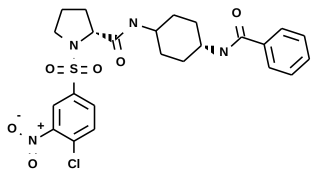

CE_C068

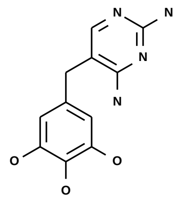

FP_0161

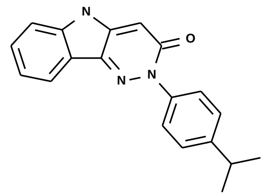

ALB-H10726215

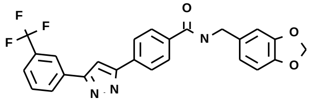

ALB-H01848004

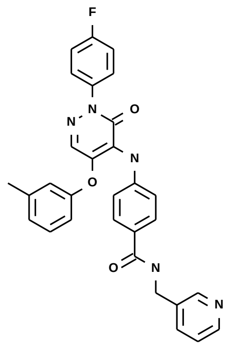

ALB-H00735714

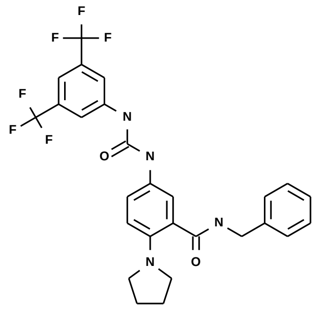

IP_0076

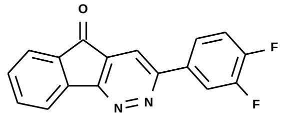

ALB-H00748802

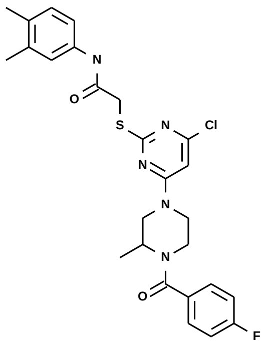

NB0028

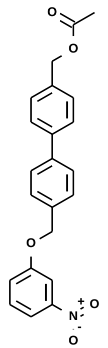

ALB-H04016914

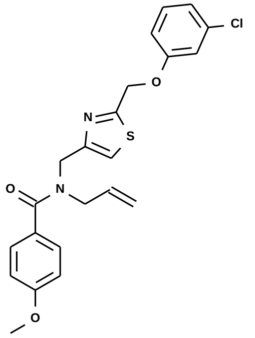

AQ_0022

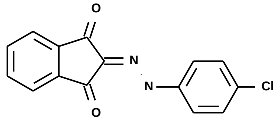

ALB-H01128507

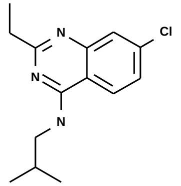

ALB-H10722128

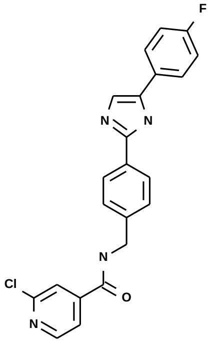

ALB-H10729325

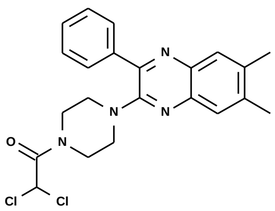

ALB-H10707802

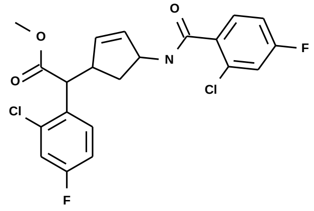

ALB-H10708842

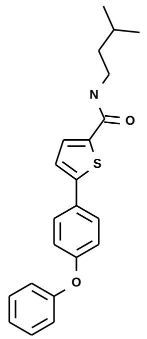

ALB-H10735471

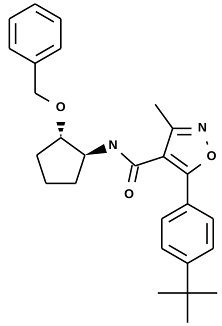

ALB-H10721631

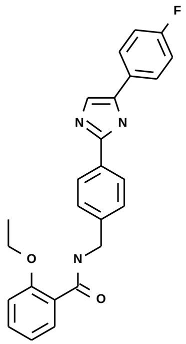

ALB-H01472397

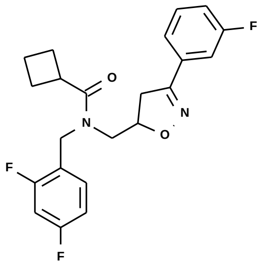

ALB-H00881482

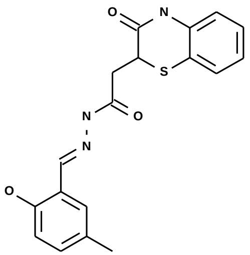

ALB-H10721537

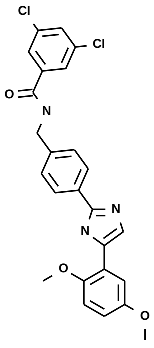

ALB-H04324916

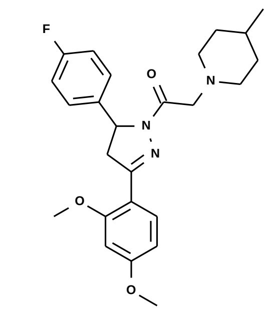

ALB-H10742806

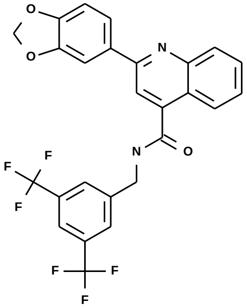

ALB-H10706059

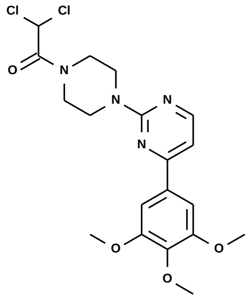

ALB-H10729982

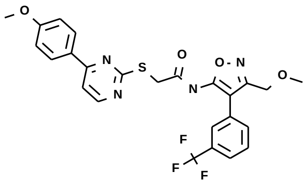

ALB-H10724339

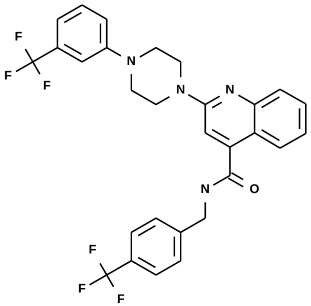

ALB-H03037430

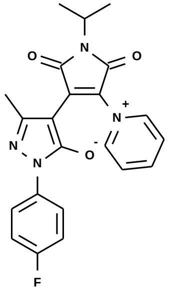

ALB-H10711821

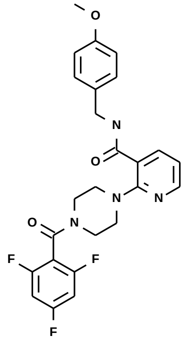

ALB-H10717173

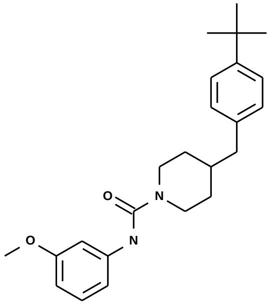

ALB-H03199406

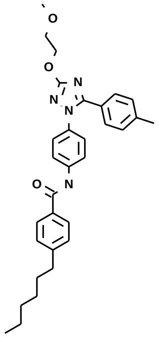

BM0447

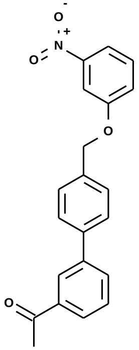

ALB-H10742776

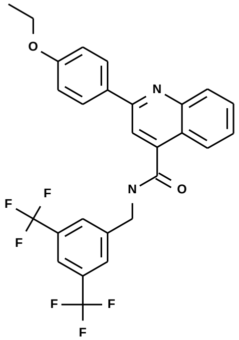

ALB-H03114363

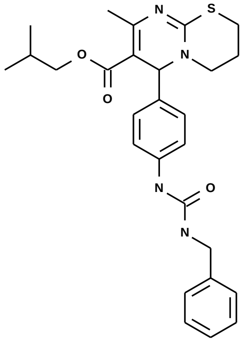

ALB-H00748922

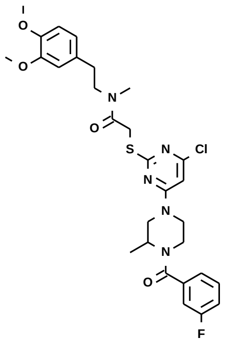

LP_0194

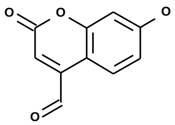

ALB-H10721559

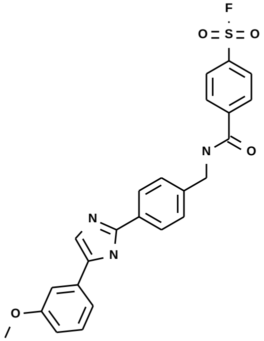

ALB-H01243227

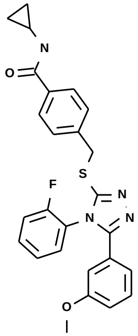

ALB-H10726740

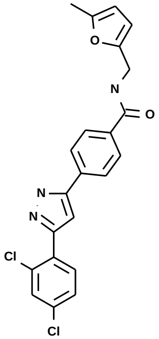

ALB-H03051787

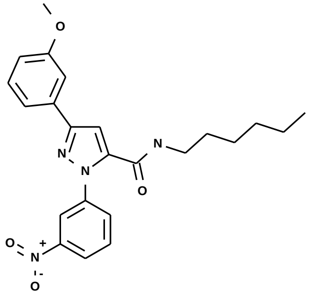

ALB-H10726841

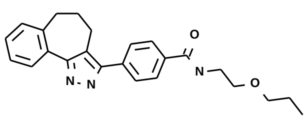

ALB-H04013163

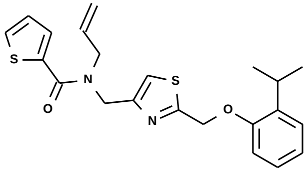

ALB-H10722264

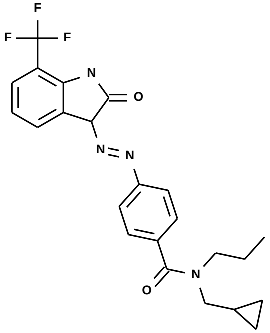

ALB-H01832702

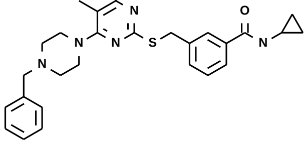

RA_0189

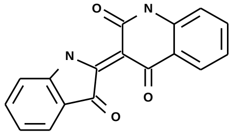

ALB-H09255461

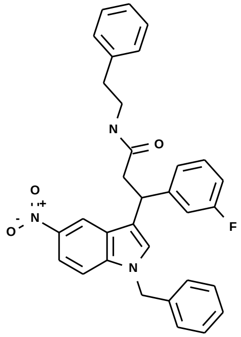

ALB-H10747242

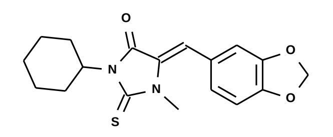

ALB-H03132805

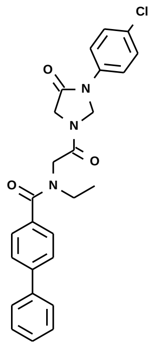

ALB-H01332768

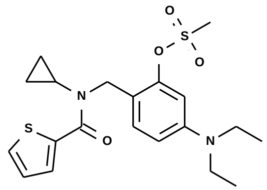

ALB-H01406084

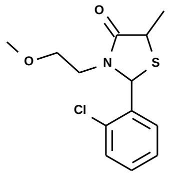

ALB-H01151710

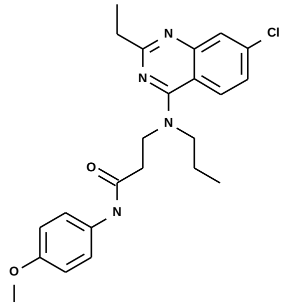

ALB-H04334205

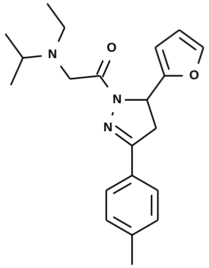

ALB-H00740967

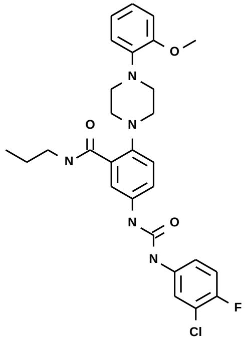

LEU_065

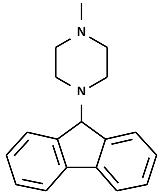

ALB-H09952353

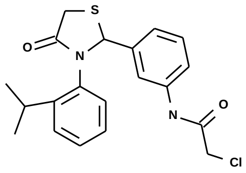

ALB-H01139275

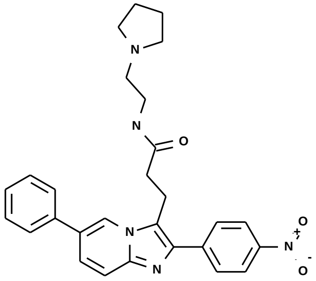

ALB-H10721785

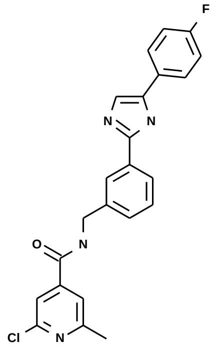

ALB-H10721911

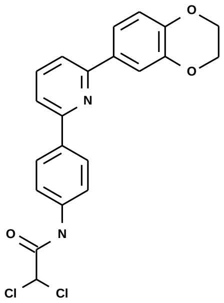

ALB-H10708673

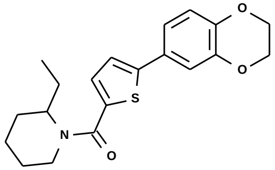

ALB-H04045182

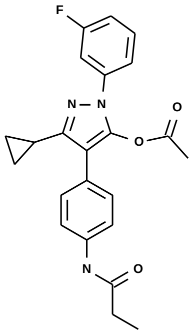

ALB-H10744883

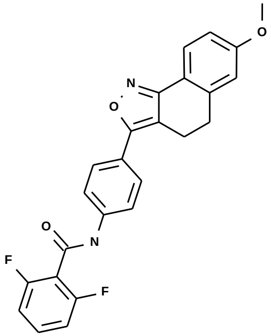

ALB-H10721847

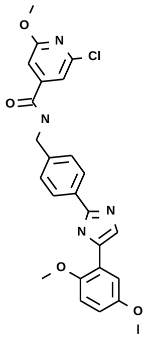

ALB-H04143833

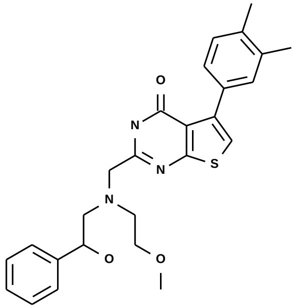

FC74

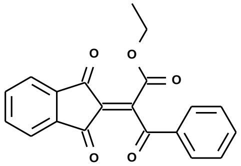

ALB-H04417939

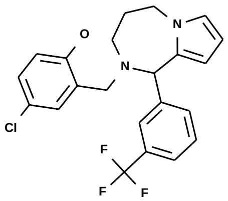

ALB-H10730960

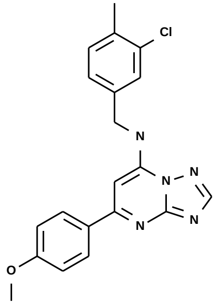

ALB-H01293999

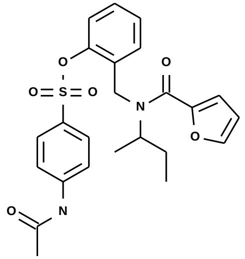

ALB-H01130458

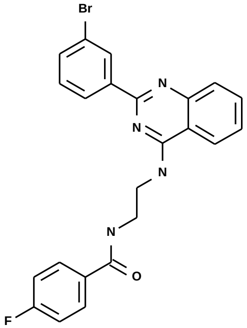

ALB-H04153206

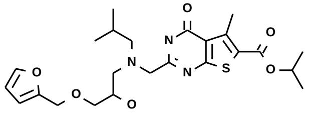

ALB-H00695267

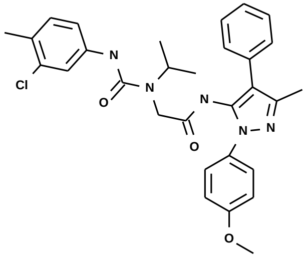

ALB-H09064715 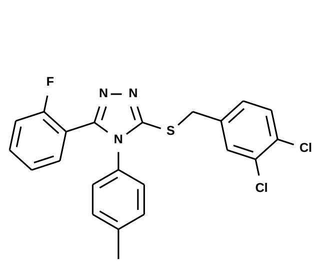	ALB-H10744553 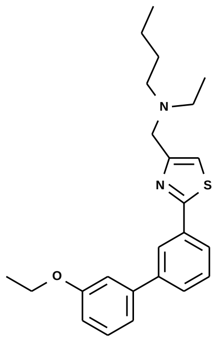	ALB-H01130102 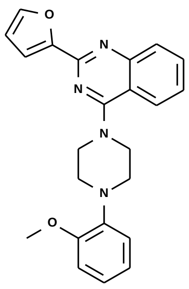	ALB-H00883972 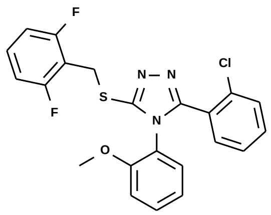	FP_0120 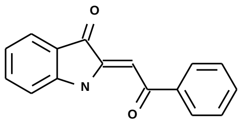	ALB-H01130434 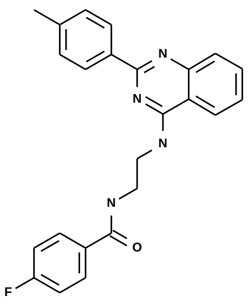
ALB-H01851593 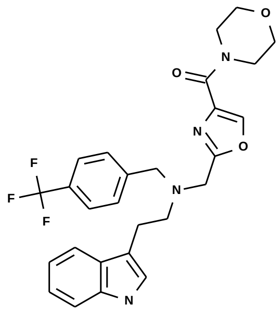	ALB-H04142019 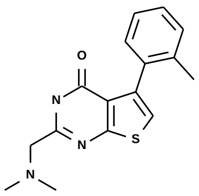	ALB-H04413622 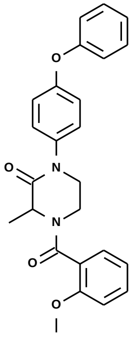	ALB-H10746644 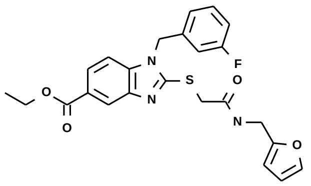	ALB-H00695766 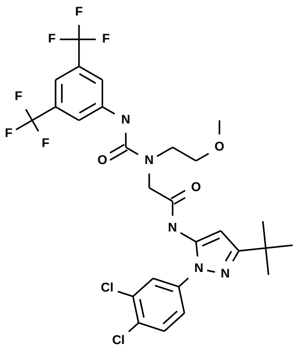	DID_058 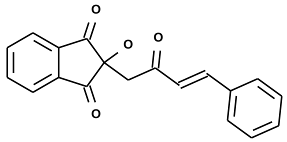
ALB-H10711527 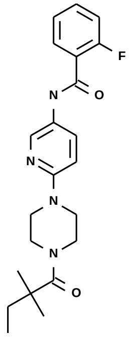	ALB-H04298034 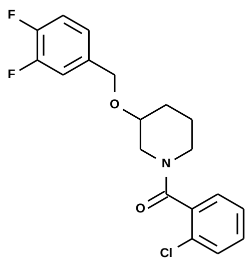	ALB-H10720266 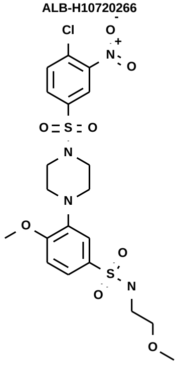	ALB-H09065100 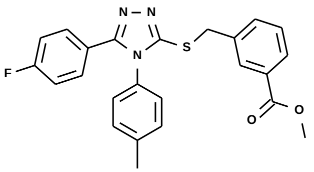	ALB-H01129322 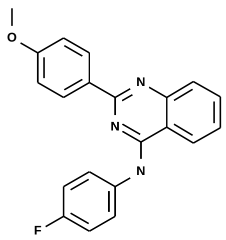	ALB-H01851560 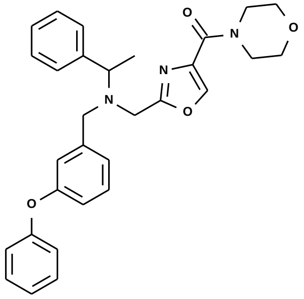
ALB-H00752521 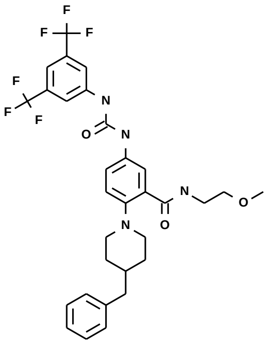	ALB-H01129422 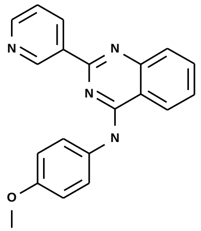	ALB-H00824371 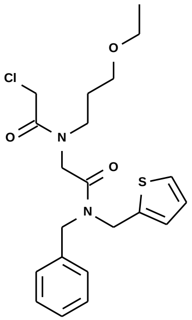	ALB-H01835558 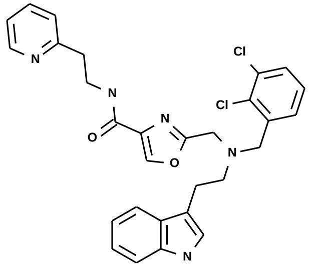	ALB-H10718985 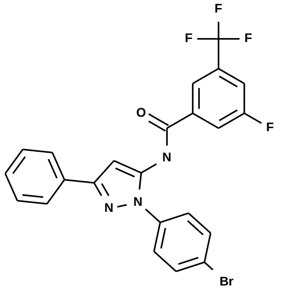	ALB-H09509601 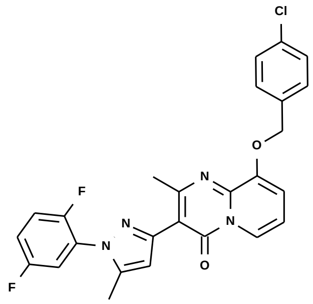

ALB-H04017974

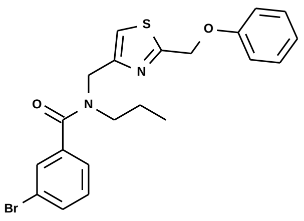

ALB-H10747384

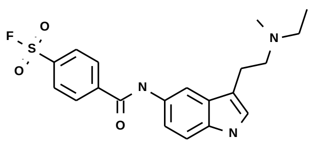

ALB-H04354866

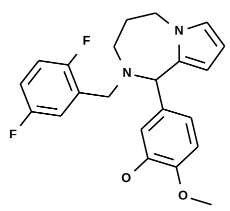

ALB-H10725865

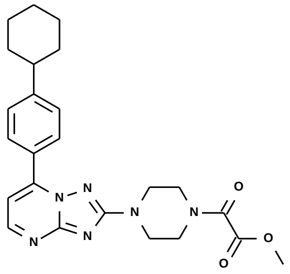

ALB-H10726355

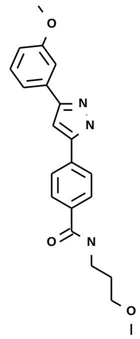

ALB-H10109575

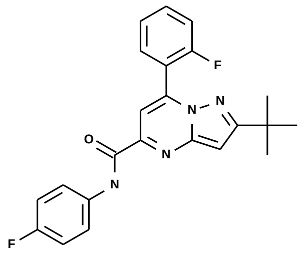

ALB-H04003567

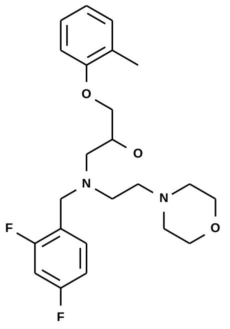

RA_0191

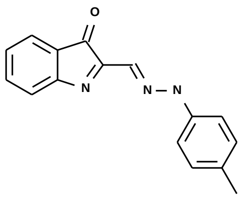

ALB-H10705716

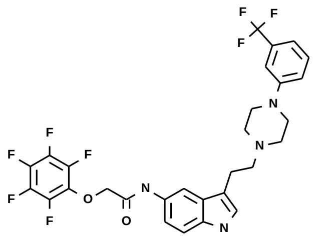

ALB-H01288828

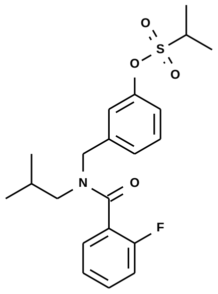

ALB-H10705644

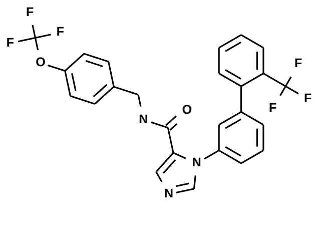

ALB-H09509027

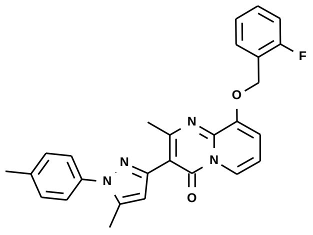

ALB-H09065428

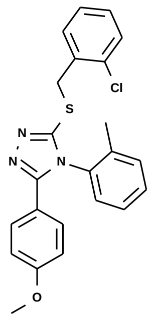

ALB-H10736734

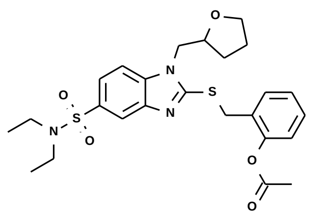

ALB-H10706955

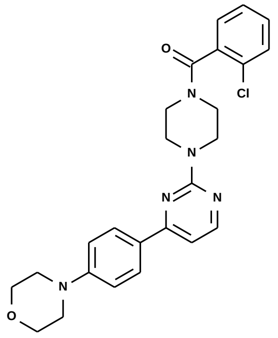

ALB-H10712333

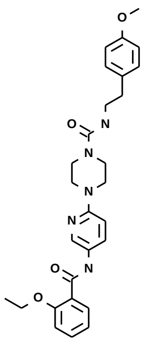

CE_C075

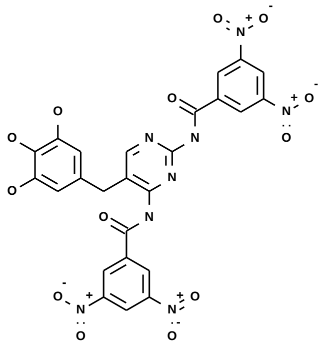

ALB-H04098122

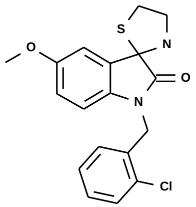

MC_0279

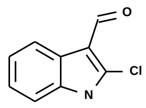

MC_0575

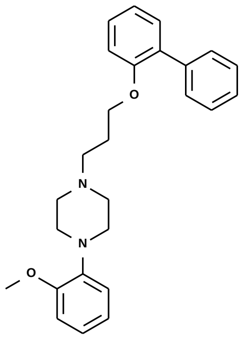

ALB-H00849826

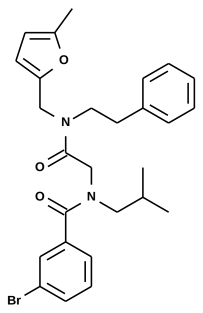

ALB-H04354419

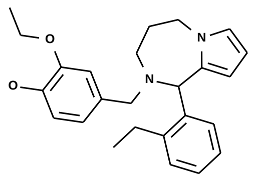

MC_0309

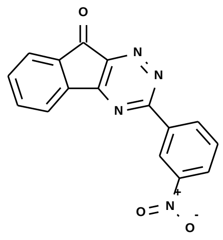

ALB-H01894436

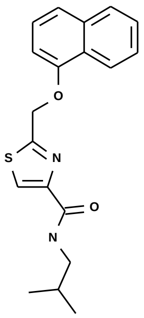

ALB-H09460191

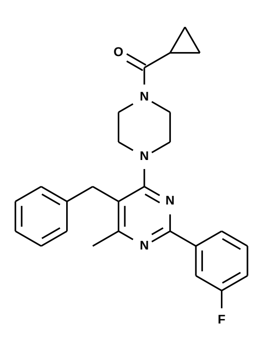

ALB-H04018427

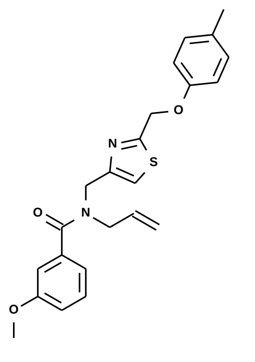

ALB-H04267640

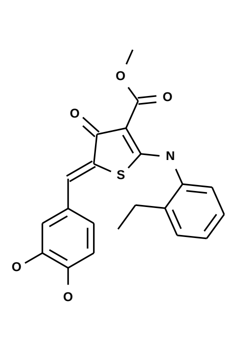

ALB-H09733130

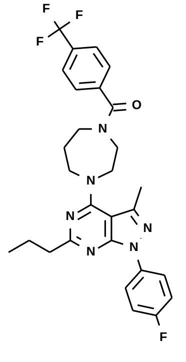

ALB-H04019419

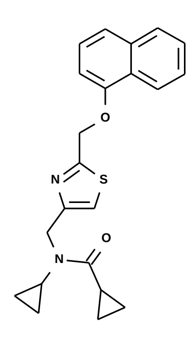

ALB-H04019294

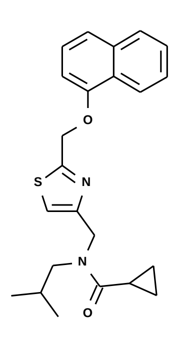

ALB-H10727290

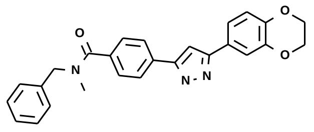

ALB-H10707456

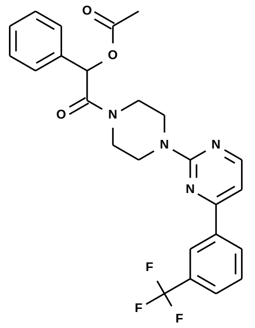

ALB-H03338145

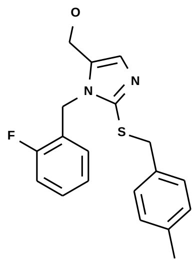

MA16

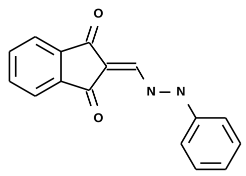

ALB-H01751063

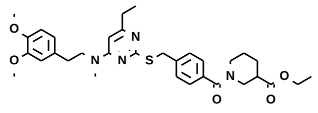

ALB-H10705976

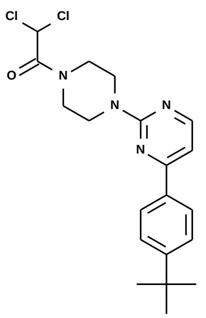

IP_0025

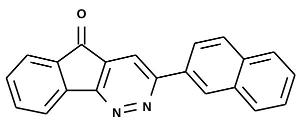

ALB-H01470034

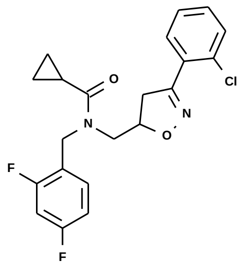

ALB-H01260511

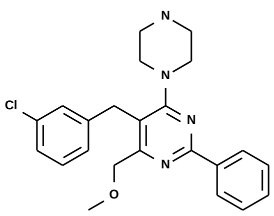

ALB-H04085773

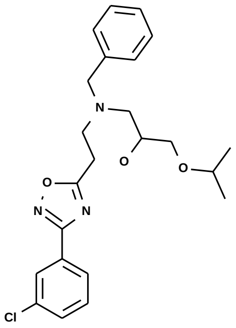

ALB-H10728258

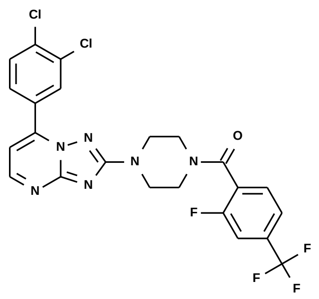

ALB-H10748669

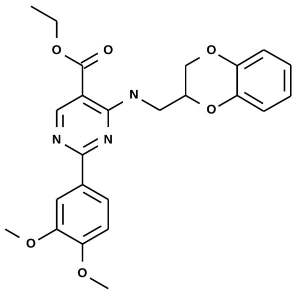

ALB-H04080256

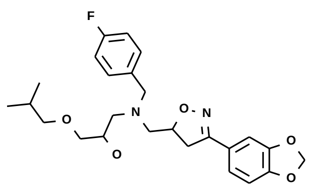

ALB-H10711539

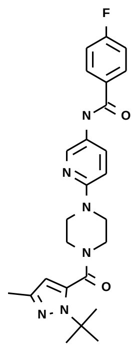

ALB-H10716124

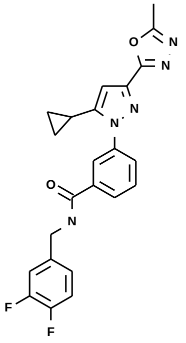

ALB-H03236487

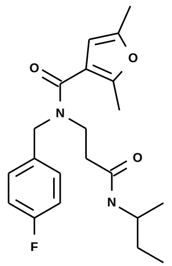

ALB-H01002268

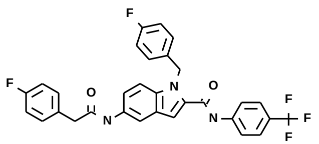

FP_0159

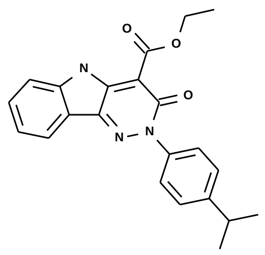

FP_0159

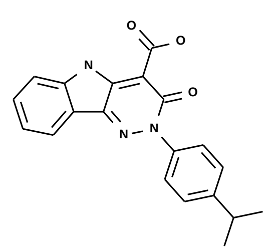

ALB-H09729755

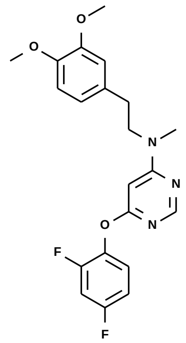

ALB-H09615654

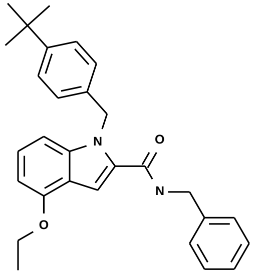

ALB-H10729321

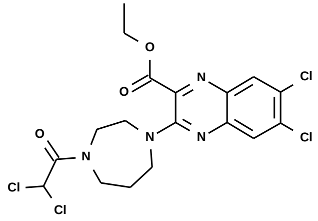

ALB-H01333296

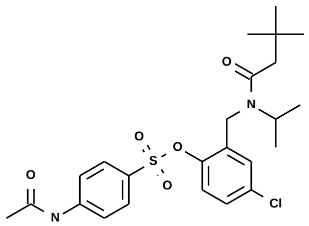

ALB-H03203531

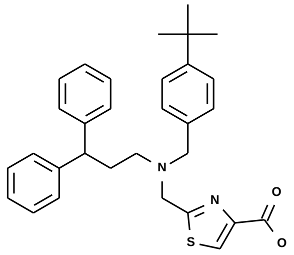

ALB-H04286962

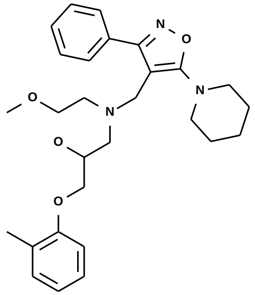

ALB-H09064705

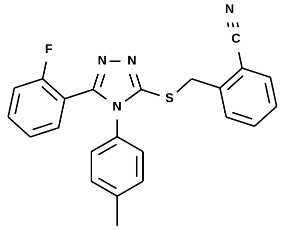

ALB-H01133698

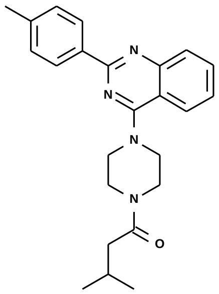

ALB-H03038259

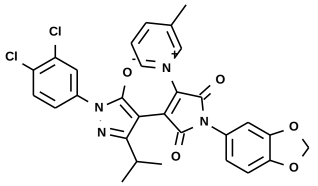

ALB-H09545384

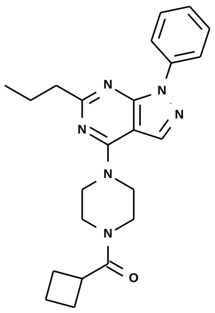

ALB-H09453615

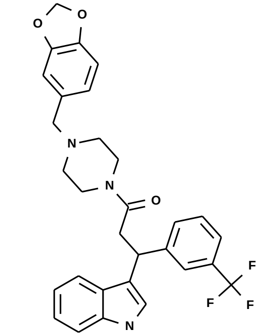

ALB-H09829381

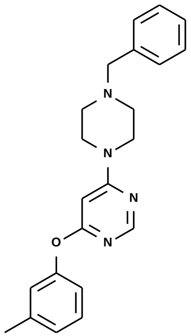

ALB-H04283003

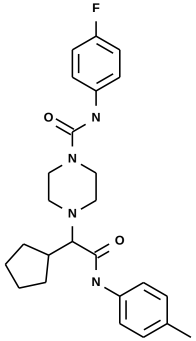

ALB-H04418298

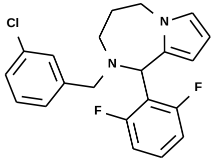

ALB-H10728691

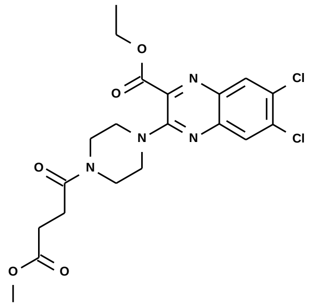

ALB-H01995746

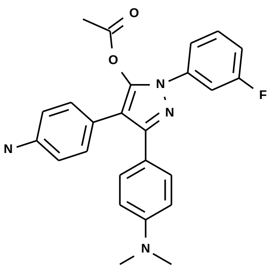

ALB-H05433944

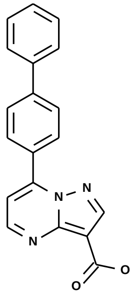

ALB-H10728698

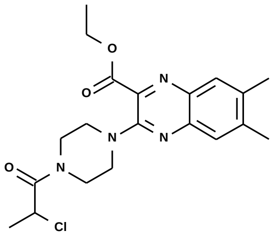

ALB-H09066839

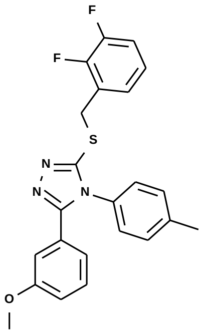

ALB-H10707942

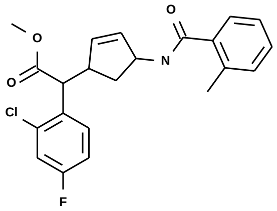

ALB-H01291632

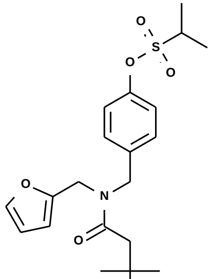

ALB-H01299953

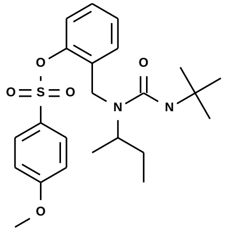

ALB-H09087015

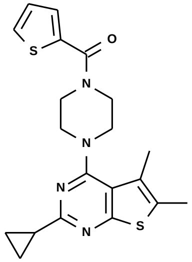

ALB-H09449983	ALB-H10721841	ALB-H10723161	ALB-H10725834	ALB-H01998559	ALB-H10738882
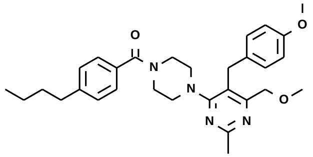	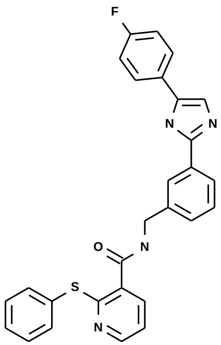	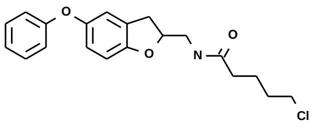	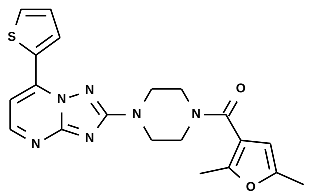	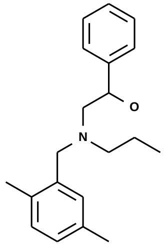	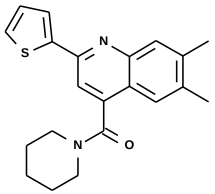
ALB-H04088423	ALB-H10735179	ALB-H10723272	ALB-H01472413	ALB-H09018917	ALB-H01997030
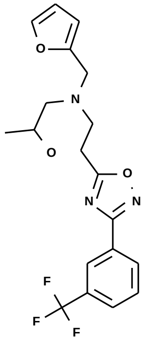	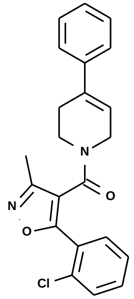	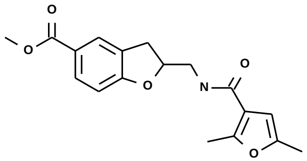	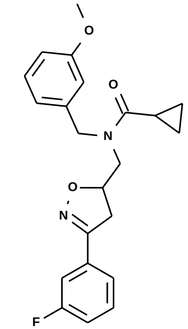	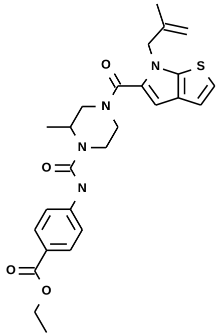	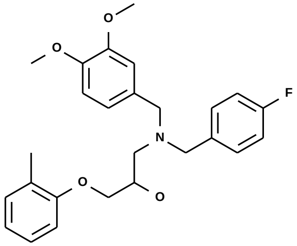
ALB-H02132937	ALB-H01822240	ALB-H10732839	ALB-H10747417	DID_080	ALB-H10709990
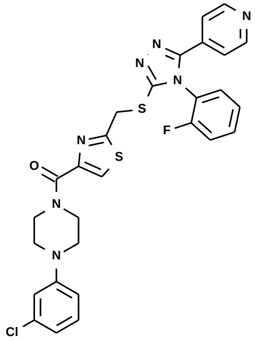	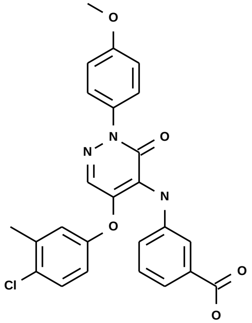	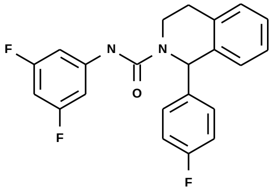	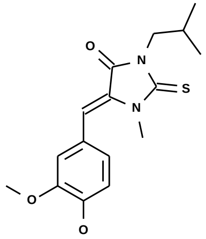	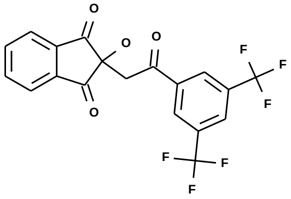	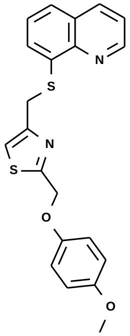
ALB-H09067275	ALB-H04045133	ALB-H04124557	LEU_117	ALB-H04268157	ALB-H00814576
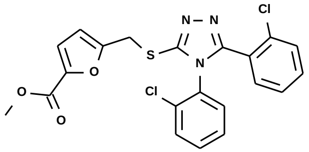	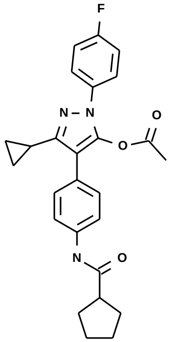	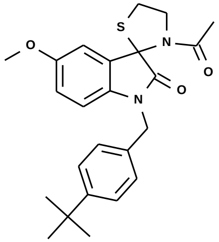	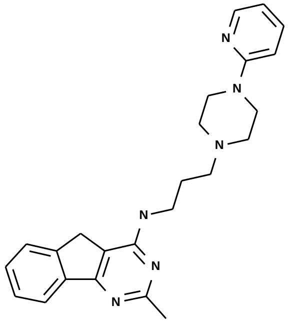	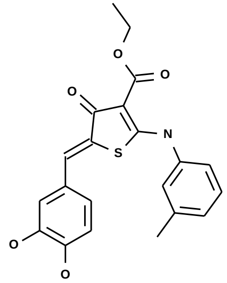	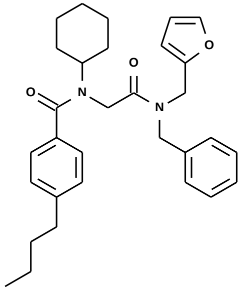

ALB-H10722450

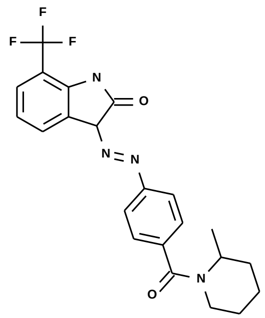

ALB-H01813536

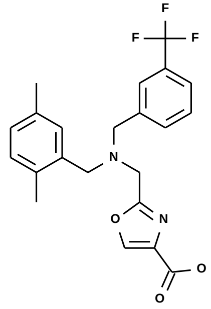

ALB-H04351847

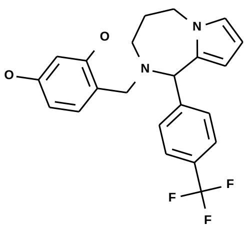

ALB-H04002044

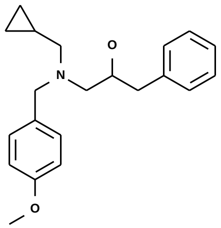

ALB-H04027795

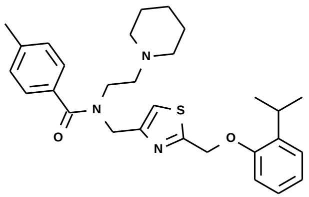

ALB-H04130153

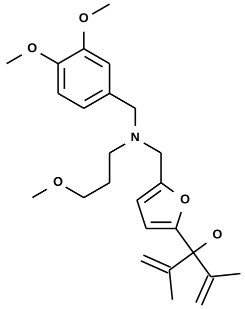

ALB-H10721048

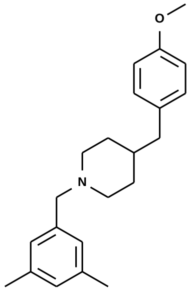

ALB-H03049561

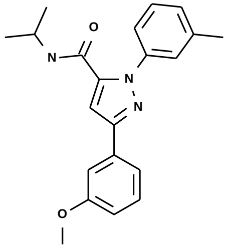

ALB-H04043494

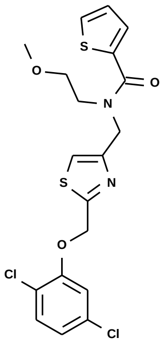

ALB-H01742855

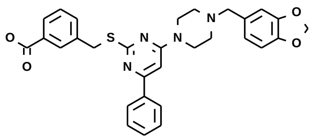

ALB-H04142631

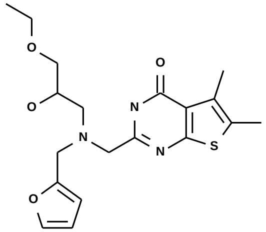

ALB-H10711494

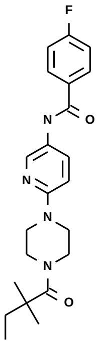

LEU_122

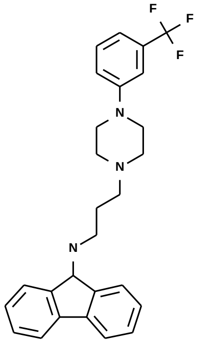

ALB-H00740414

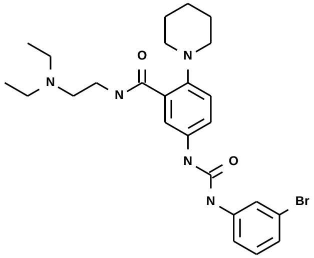

FP_0201

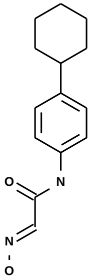

ALB-H01901798

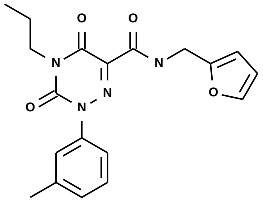

ALB-H10721016

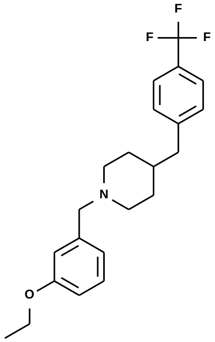

ALB-H10721076

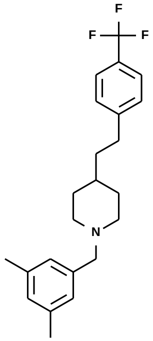

LEU_050

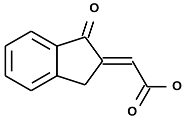

ALB-H03168506

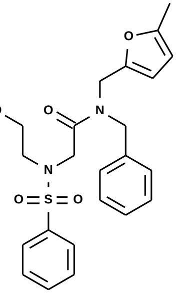

ALB-H04017177

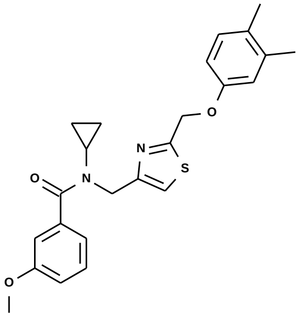

ALB-H10714452

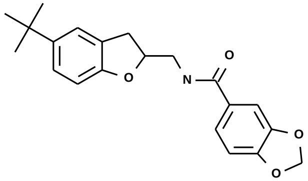

ALB-H03341365

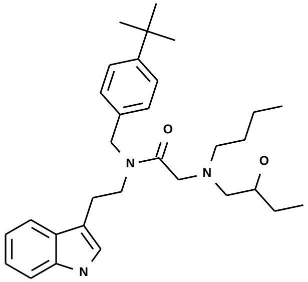

ALB-H10747197

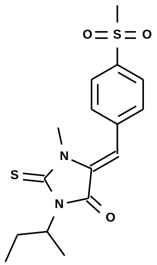

ALB-H03339487

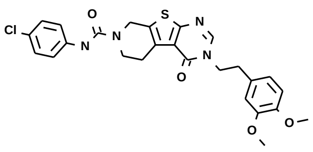

ALB-H10727289

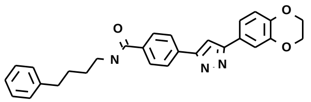

ALB-H04088434

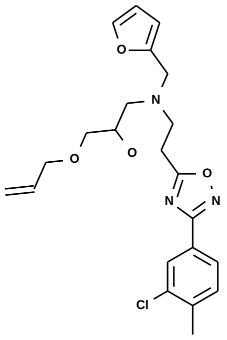

ALB-H10746789

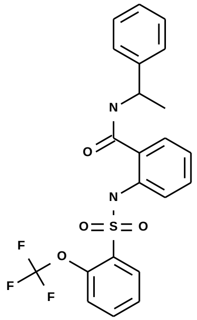

ALB-H01742478

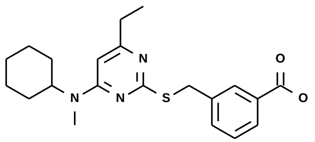

ALB-H01286589

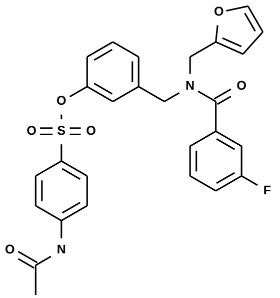

ALB-H04136859

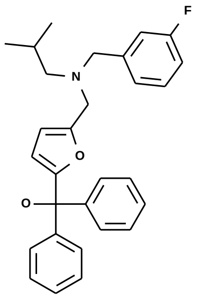

ALB-H04016853

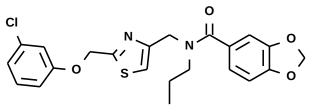

ALB-H04298432

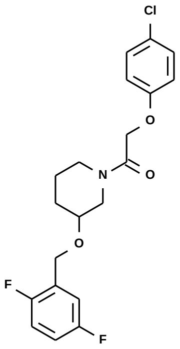

ALB-H04153893

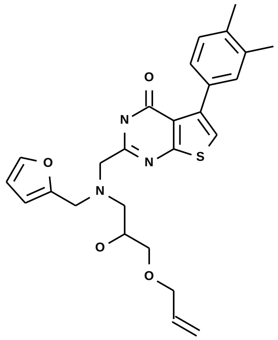

ALB-H04015796

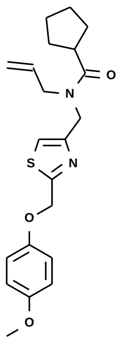

ALB-H03166827

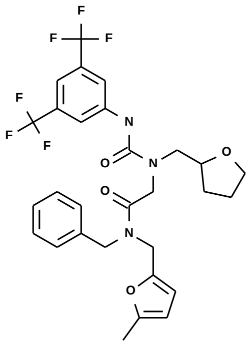

ALB-H10727041

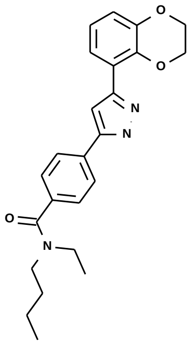

ALB-H10736923

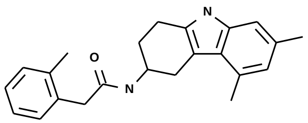

ALB-H00890866

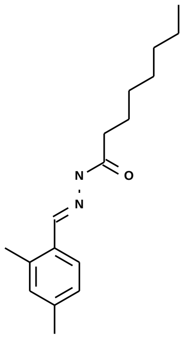

ALB-H10119217

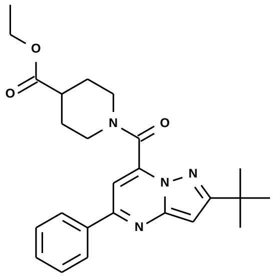

ALB-H01742819

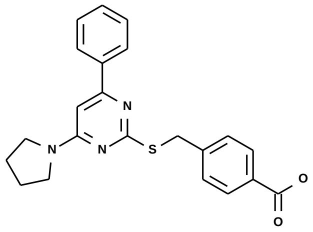

Supplement: Figure S2 — List of chemical structures showing selective cytotoxicity for HCT116 cell lines. (PDF) [file pone.0035582.s002.pdf]
