# Supplementary material for: A Chemocentric Approach to the Identification of Cancer Targets
Source: PLoS One. 2012 Apr 25;7(4):e35582. doi: 10.1371/journal.pone.0035582 (PMC3338416; doi:10.1371/journal.pone.0035582)
Supplement: Figure S3 — List of chemical structures showing selective cytotoxicity for MRC-5 cell lines. (PDF) [file pone.0035582.s003.pdf]

#1 [1 - 24]

ALB-H00441995

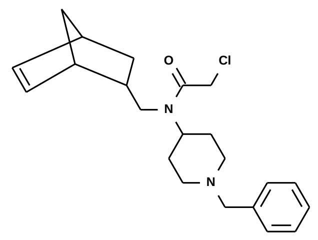

ALB-H00600049

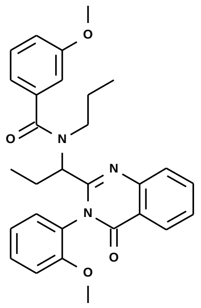

ALB-H00735197

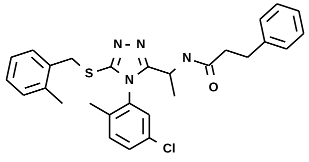

ALB-H00739584

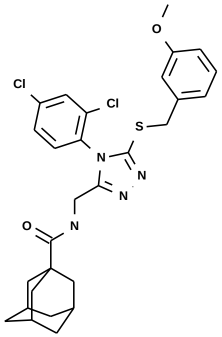

ALB-H00741434

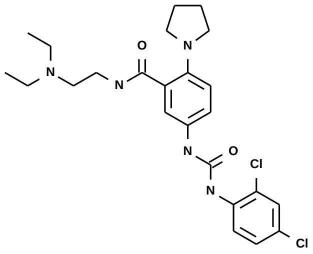

ALB-H00751202

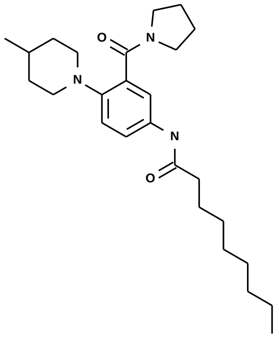

ALB-H00753699

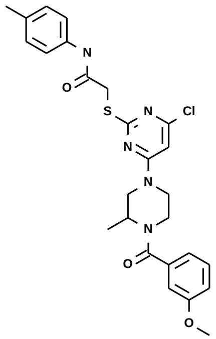

ALB-H00810012

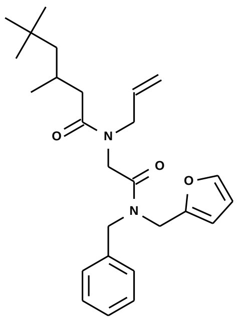

ALB-H00822700

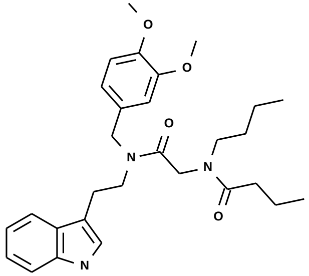

ALB-H00829834

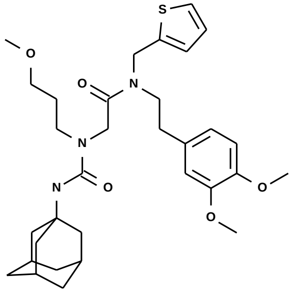

ALB-H00831742

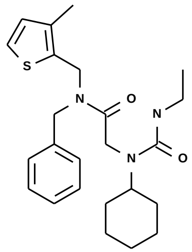

ALB-H01129023

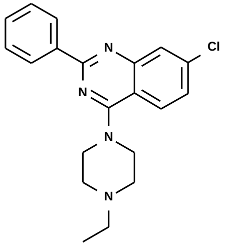

ALB-H01129467

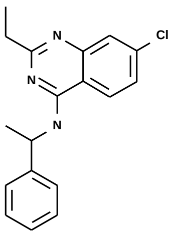

ALB-H01129723

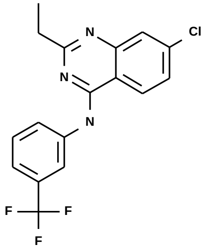

ALB-H01130455

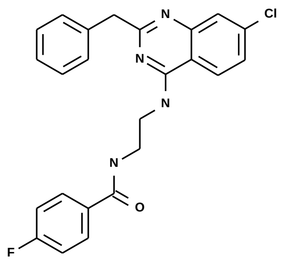

ALB-H01130627

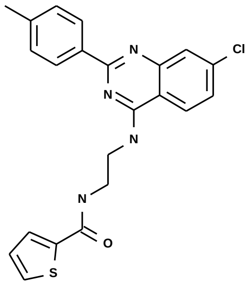

ALB-H01131842

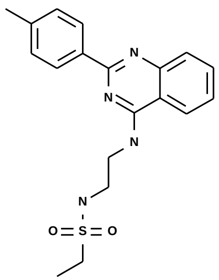

ALB-H01149429

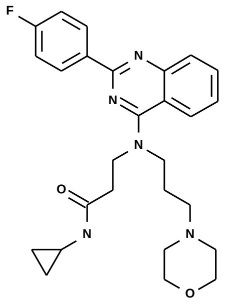

ALB-H01151015

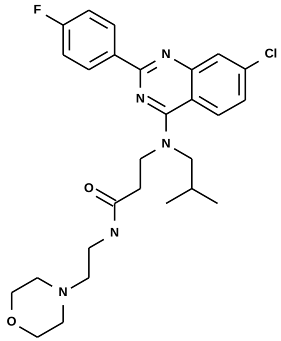

ALB-H01176322

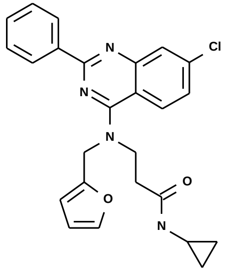

ALB-H01176330

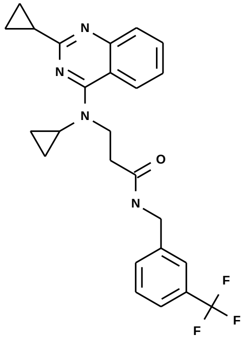

ALB-H01272585

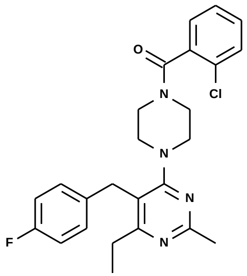

ALB-H01316829

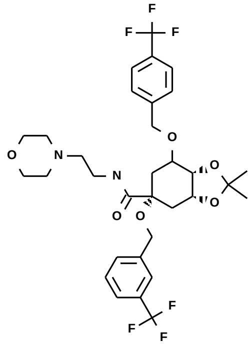

ALB-H01320674

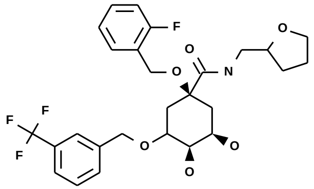

ALB-H01342715

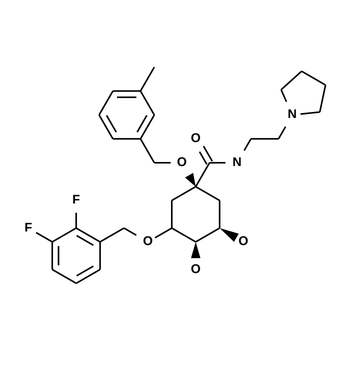

ALB-H01344748

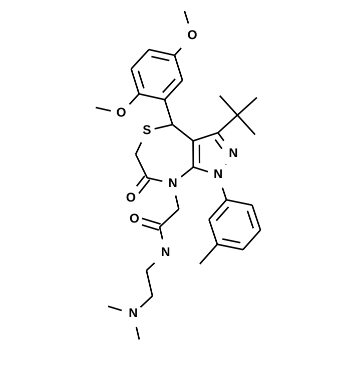

ALB-H01382499

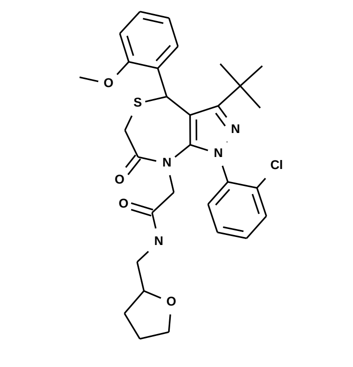

ALB-H01392886

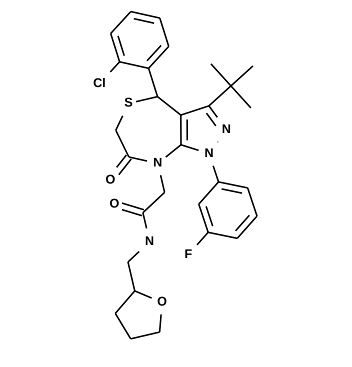

ALB-H01405942

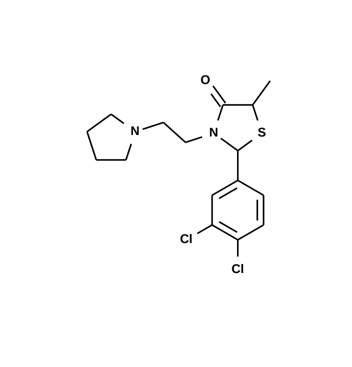

ALB-H01470571

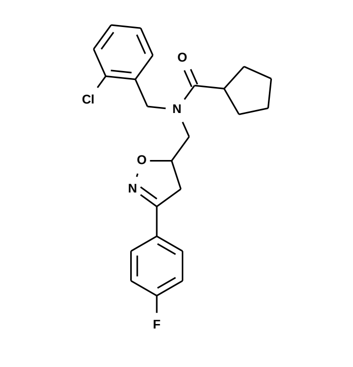

ALB-H01742822

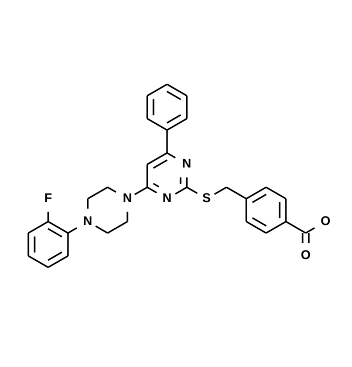

ALB-H01751149

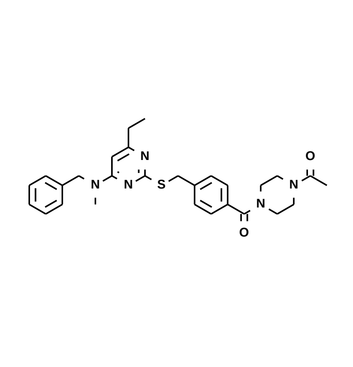

ALB-H01765518

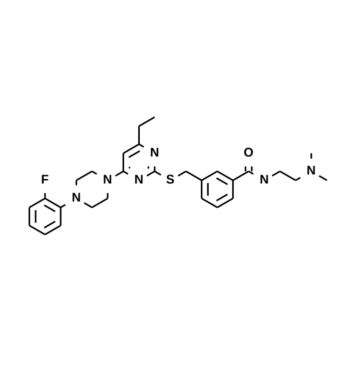

ALB-H01767751

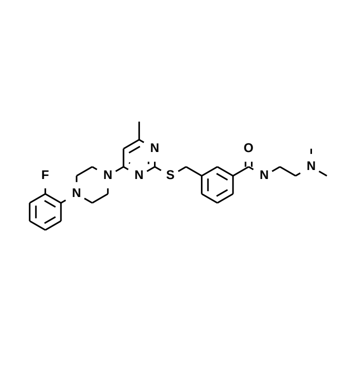

ALB-H01812746

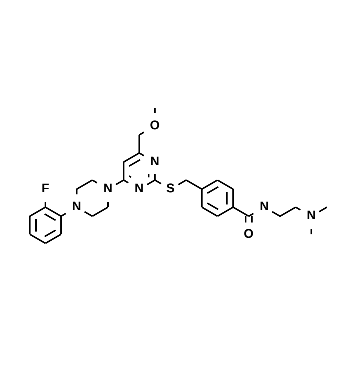

ALB-H01822262

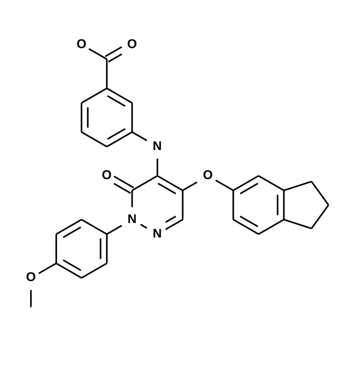

ALB-H01822263

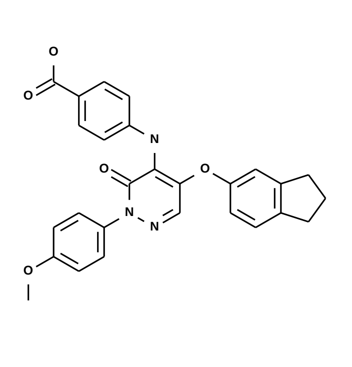

ALB-H01835822

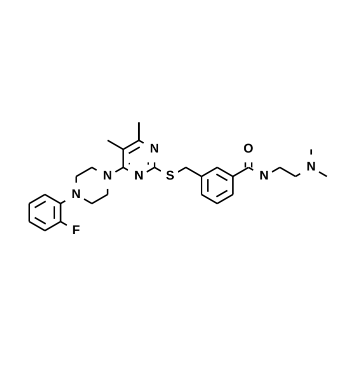

ALB-H01836063

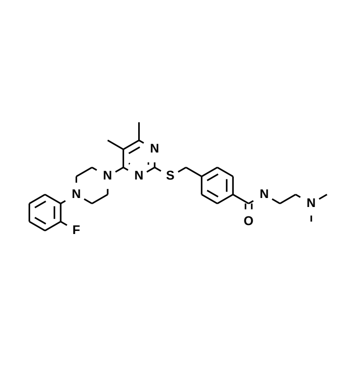

ALB-H01836388

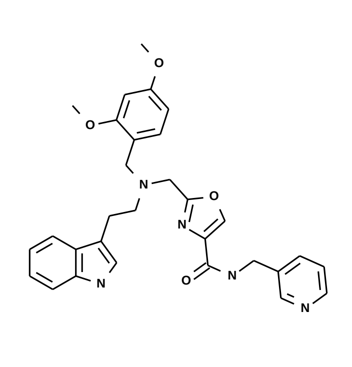

ALB-H01836519

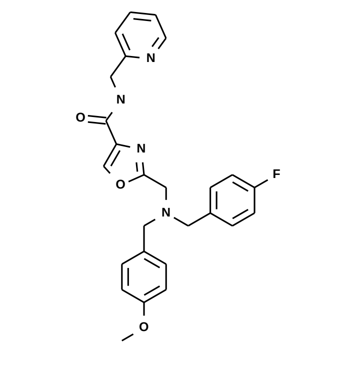

ALB-H01841312

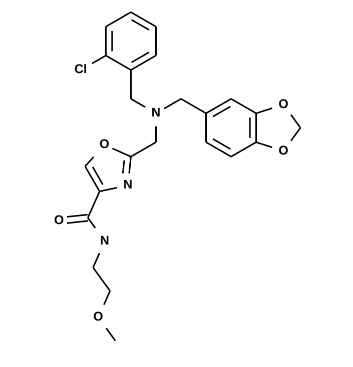

ALB-H01841782

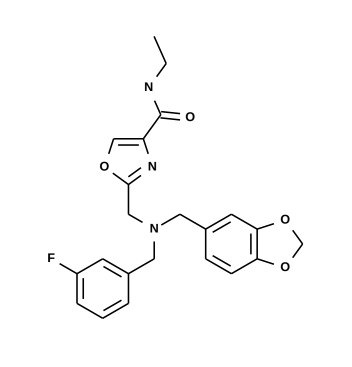

ALB-H01852110

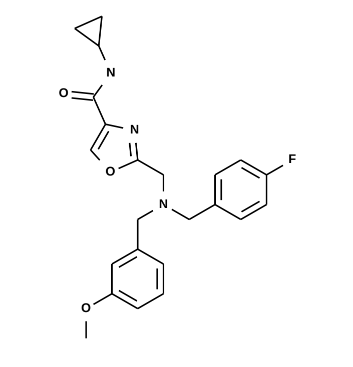

ALB-H01852117

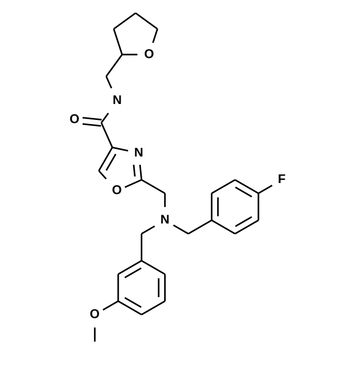

ALB-H01852266

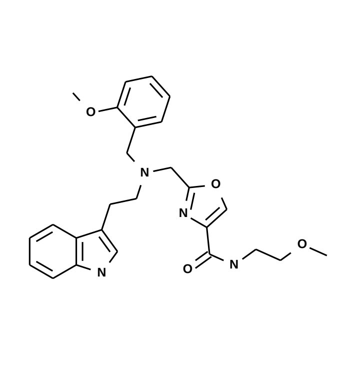

ALB-H01866312

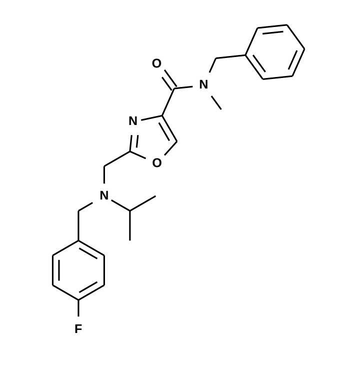

ALB-H01873363

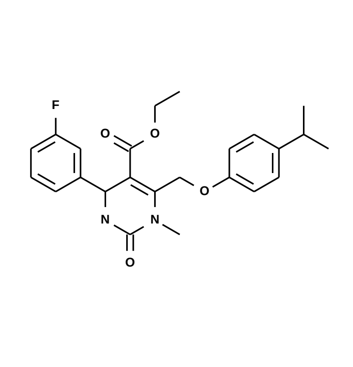

**ALB-H01873757**

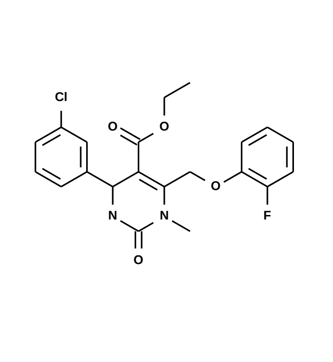

**ALB-H01996281**

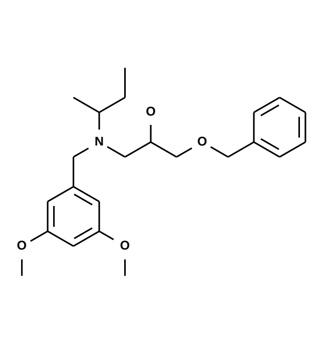

**ALB-H01996644**

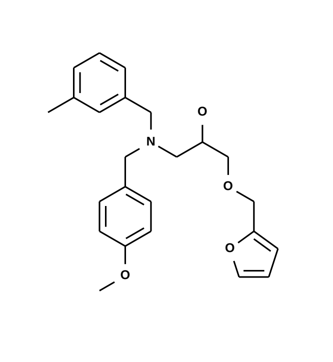

**ALB-H01997188**

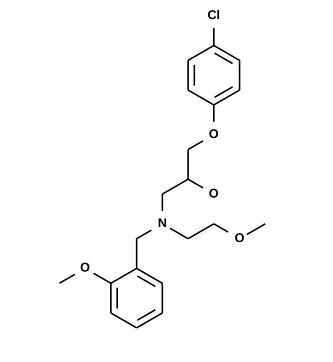

**ALB-H01997751**

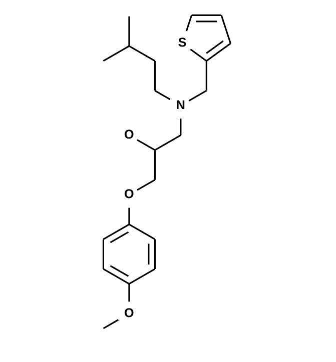

**ALB-H01998750**

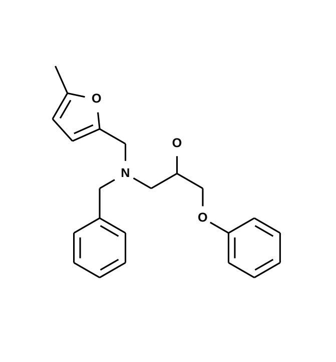

**ALB-H01999230**

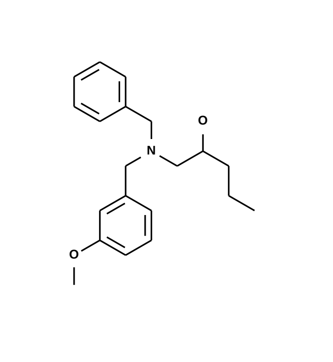

**ALB-H01999271**

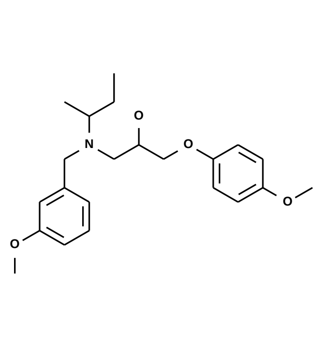

**ALB-H01999383**

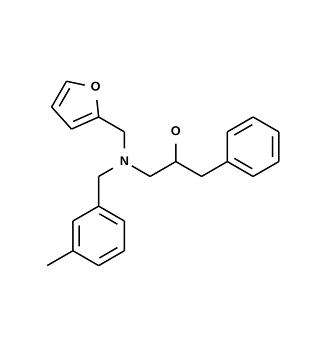

**ALB-H01999454**

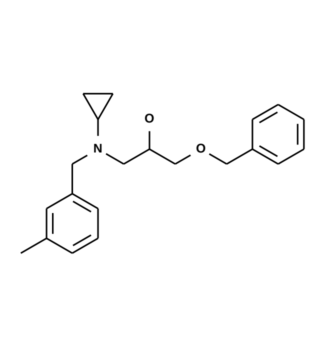

**ALB-H01999604**

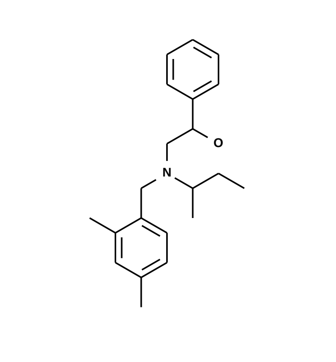

**ALB-H01999633**

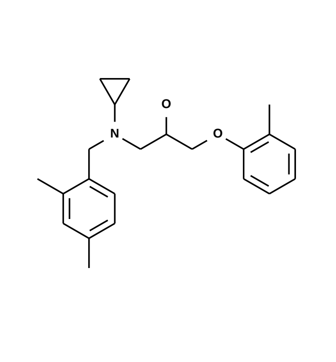

**ALB-H01999905**

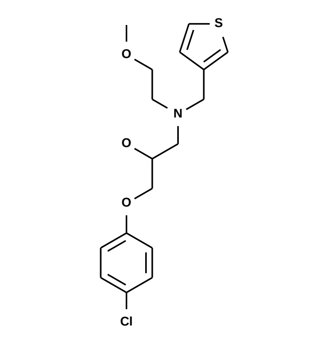

**ALB-H02000179**

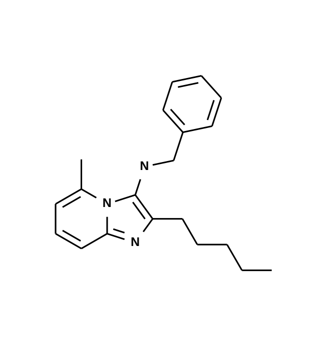

**ALB-H02001373**

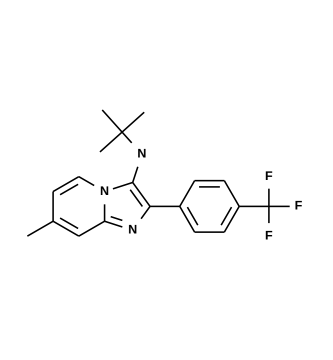

**ALB-H02002740**

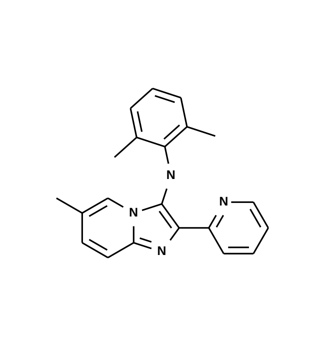

**ALB-H02015571**

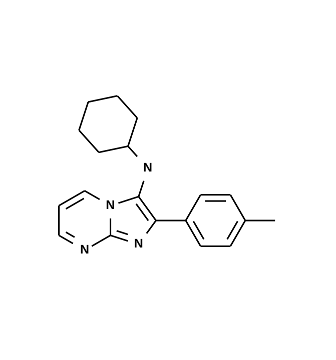

**ALB-H02015653**

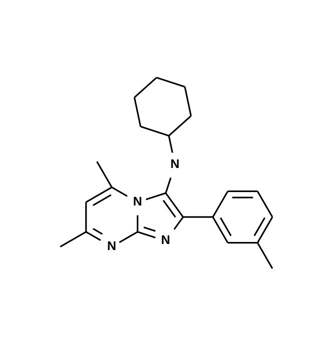

**ALB-H02021273**

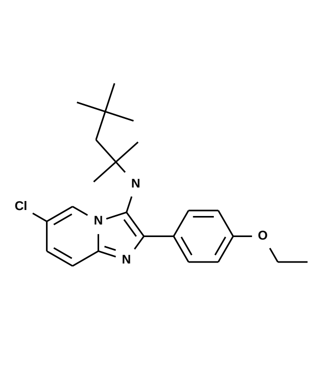

**ALB-H02121188**

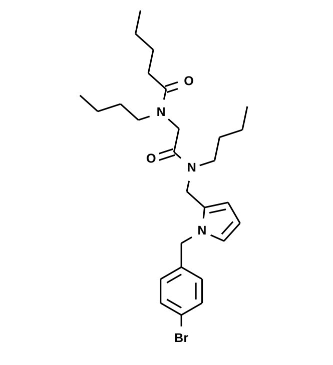

**ALB-H02146008**

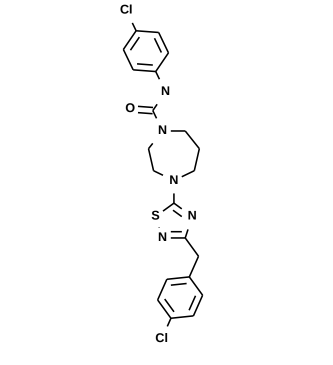

**ALB-H02164105**

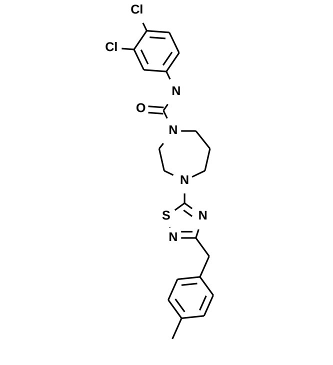

**ALB-H02201908**

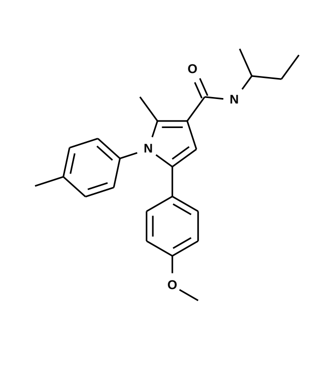

**ALB-H02204633**

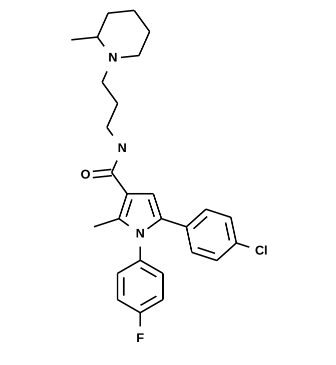

**ALB-H02206189**

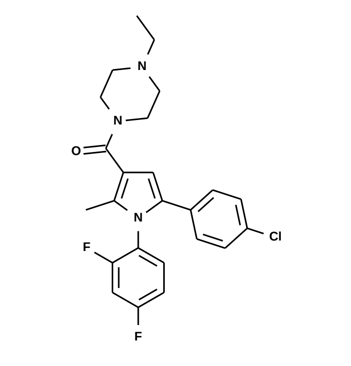

**ALB-H02217146**

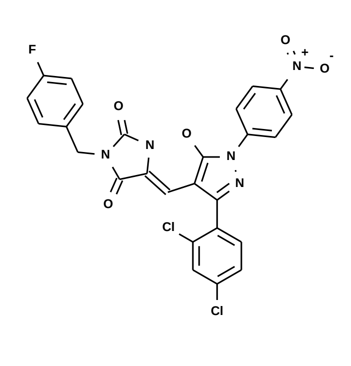

**ALB-H02217160**

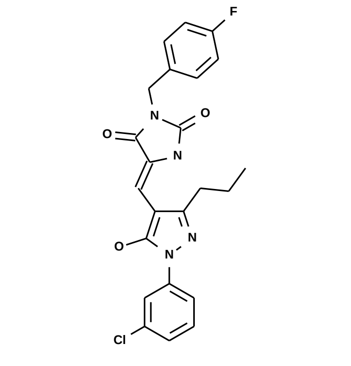

**ALB-H02217620**

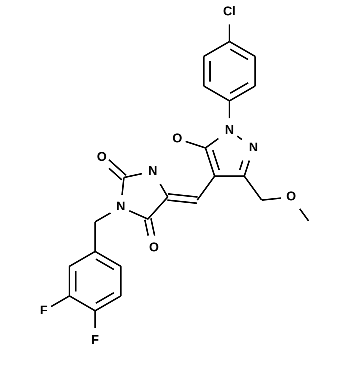

**ALB-H02217807**

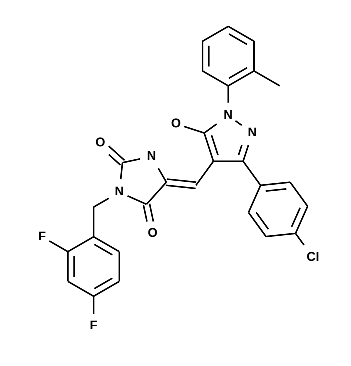

**ALB-H02217808**

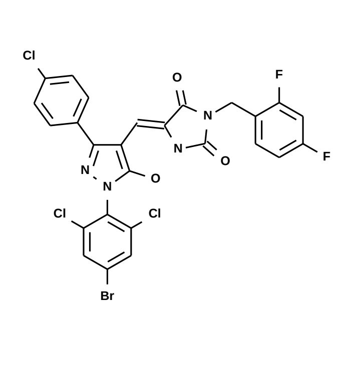

**ALB-H03004996**

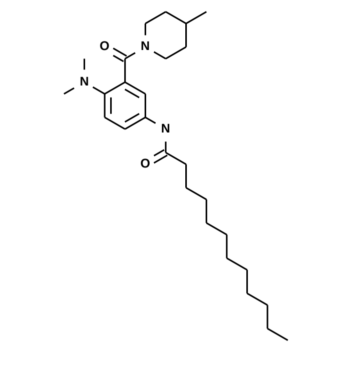

**ALB-H03016026**

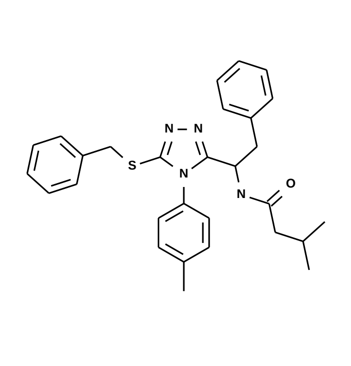

**ALB-H03066396**

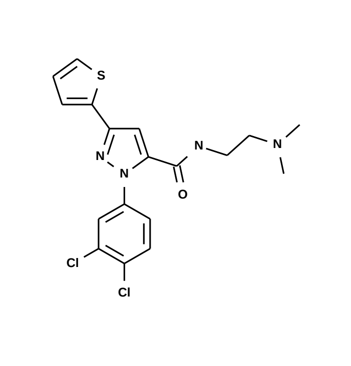

**ALB-H03114063**

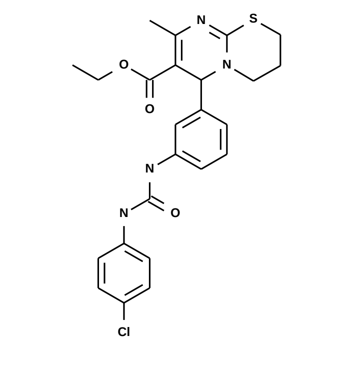

**ALB-H03134961**

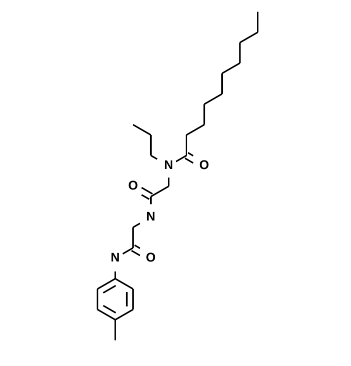

**ALB-H03161338**

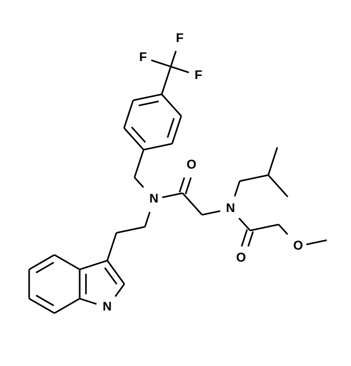

**ALB-H03162903**

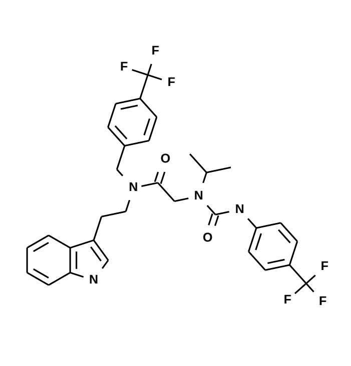

**ALB-H03162935**

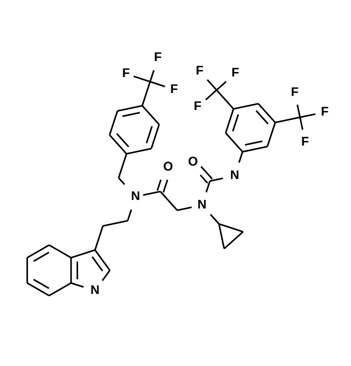

**ALB-H03168075**

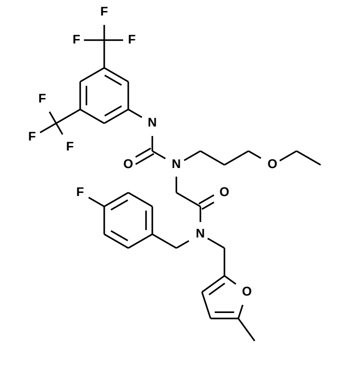

**ALB-H03171195**

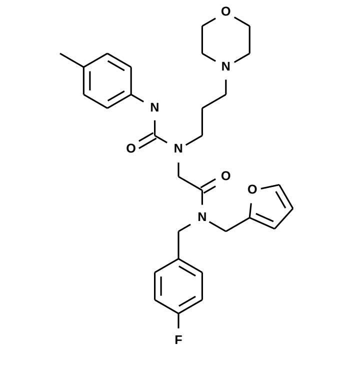

**ALB-H03171234**

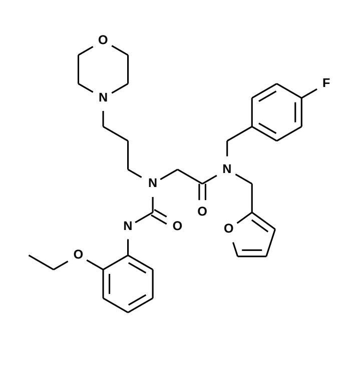

**ALB-H03182139**

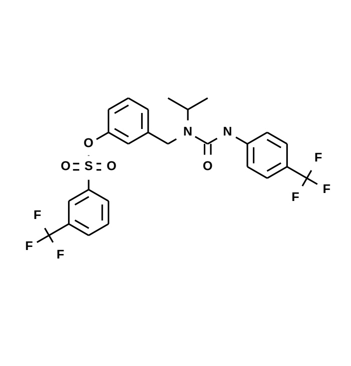

**ALB-H03183574**

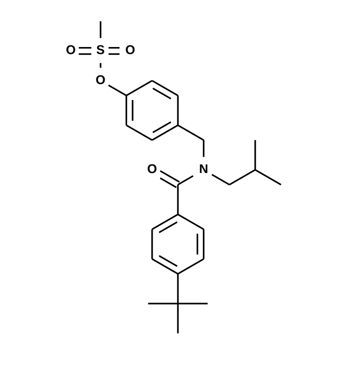

**ALB-H03185558**

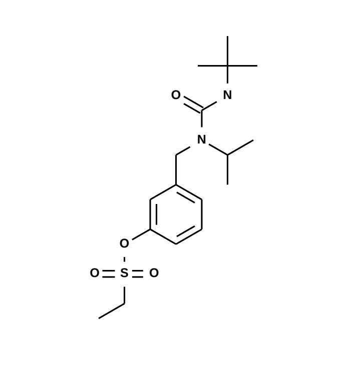

**ALB-H03193308**

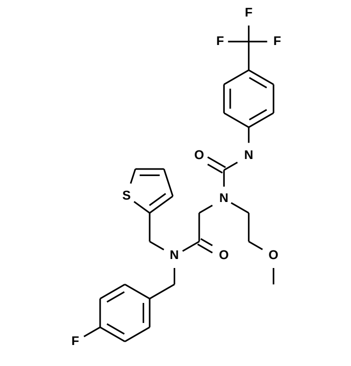

**ALB-H03194044**

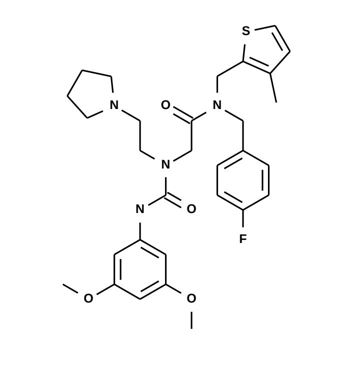

**ALB-H03334056**

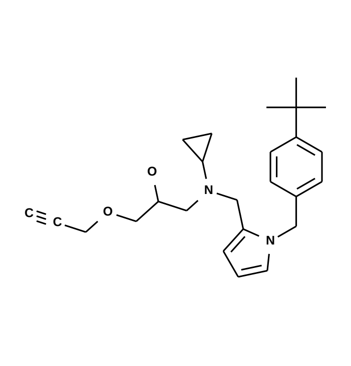

**ALB-H03334102**

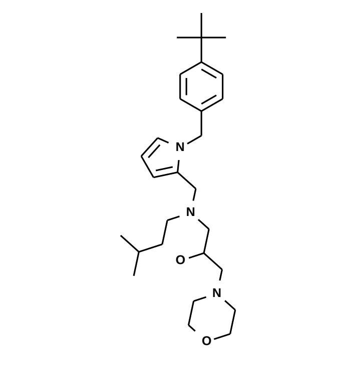

| | | | | | |
|---|---|---|---|---|---|
| **ALB-H03334222** | **ALB-H03334668** | **ALB-H03334710** | **ALB-H03336206** | **ALB-H03340074** | **ALB-H03341329** |
| 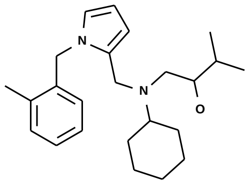 | 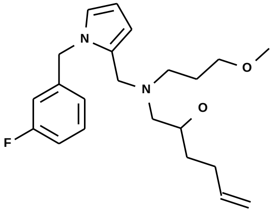 | 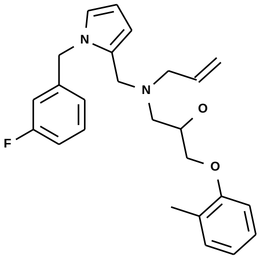 | 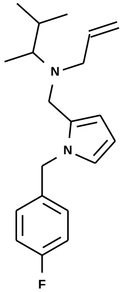 | 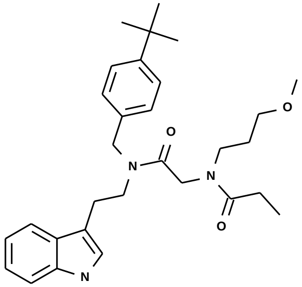 | 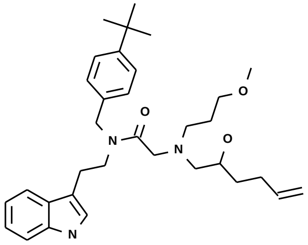 |
| **ALB-H04000068** | **ALB-H04000828** | **ALB-H04001268** | **ALB-H04001287** | **ALB-H04001486** | **ALB-H04001488** |
| 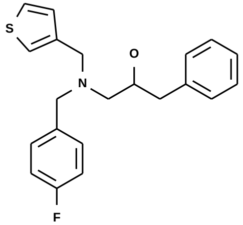 | 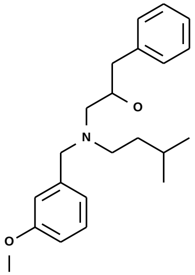 | 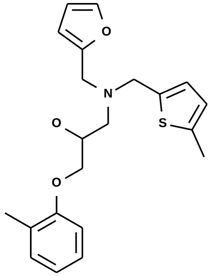 | 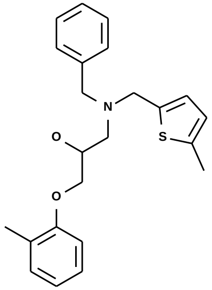 | 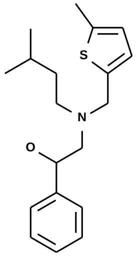 | 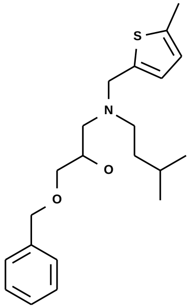 |
| **ALB-H04001490** | **ALB-H04001781** | **ALB-H04002026** | **ALB-H04002047** | **ALB-H04002176** | **ALB-H04002182** |
| 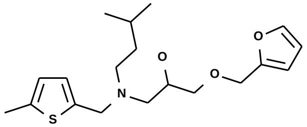 | 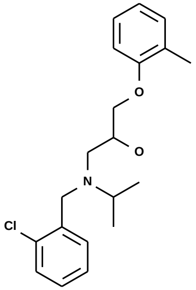 | 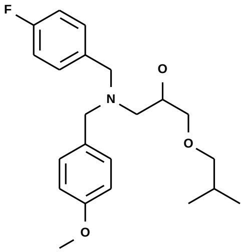 | 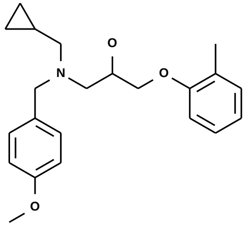 | 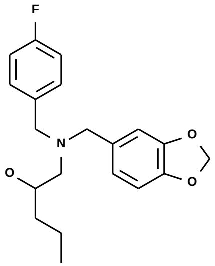 | 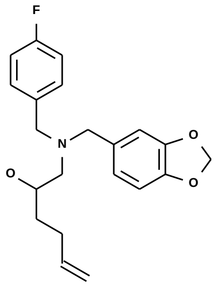 |
| **ALB-H04002467** | **ALB-H04002546** | **ALB-H04002761** | **ALB-H04002766** | **ALB-H04003637** | **ALB-H04003702** |
| 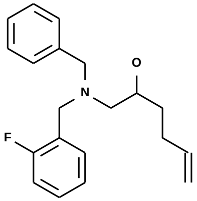 | 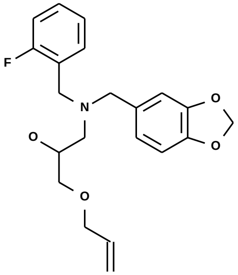 | 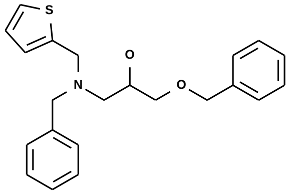 | 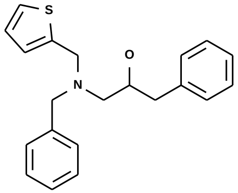 | 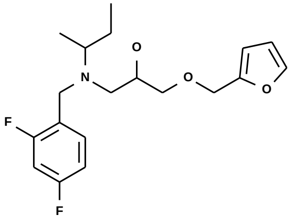 | 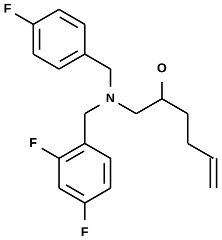 |

| | | | | | |
|---|---|---|---|---|---|
| ALB-H04003748 | ALB-H04003768 | ALB-H04004034 | ALB-H04027354 | ALB-H04032862 | ALB-H04039207 |
| 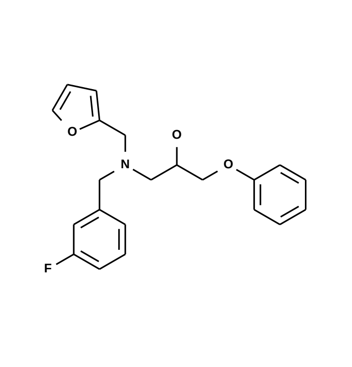 | 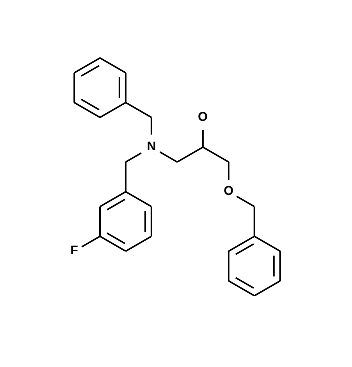 | 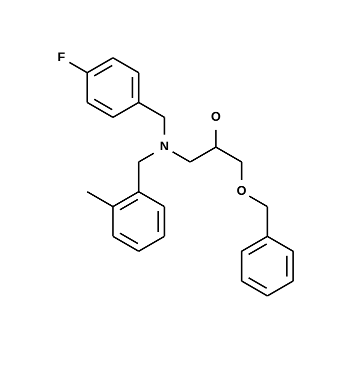 | 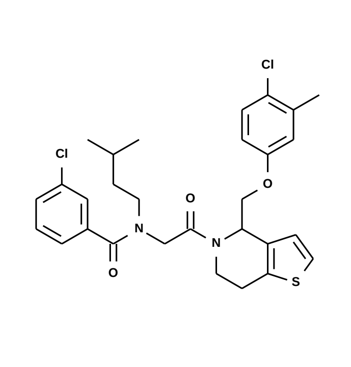 | 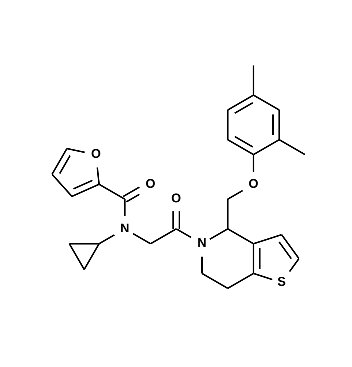 | 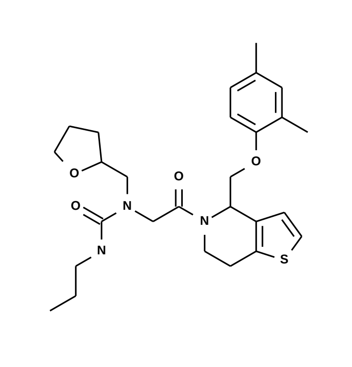 |
| ALB-H04060999 | ALB-H04079334 | ALB-H04097978 | ALB-H04106388 | ALB-H04109844 | ALB-H04138872 |
| 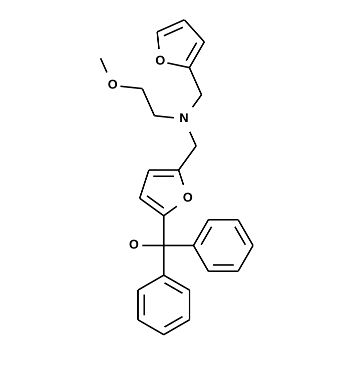 | 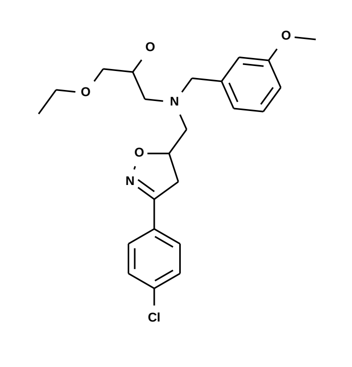 | 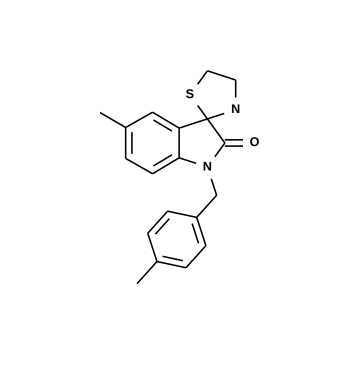 | 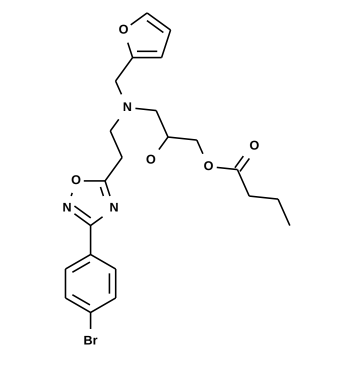 | 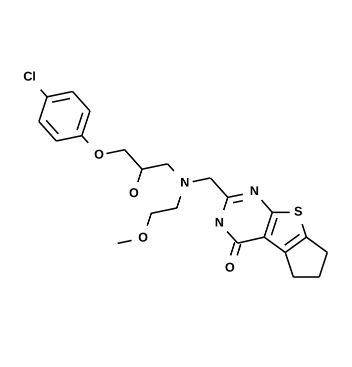 | 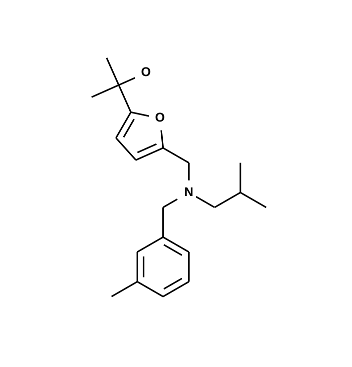 |
| ALB-H04139590 | ALB-H04147525 | ALB-H04154848 | ALB-H04245819 | ALB-H04271771 | ALB-H04273923 |
| 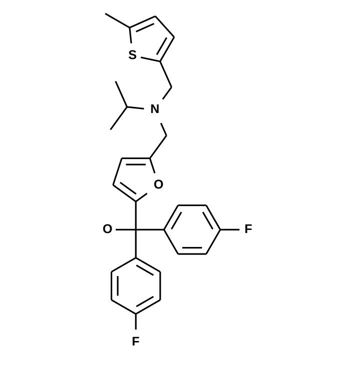 | 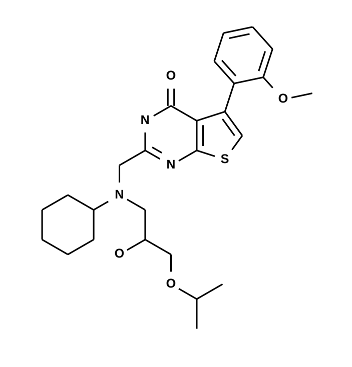 | 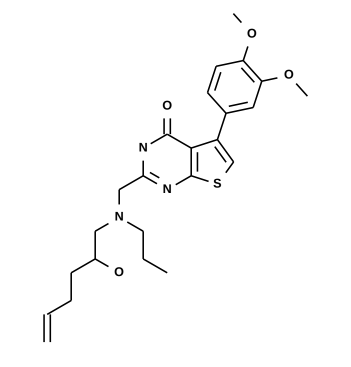 | 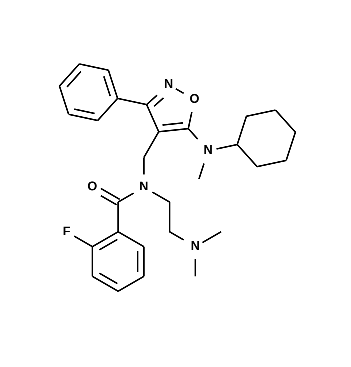 | 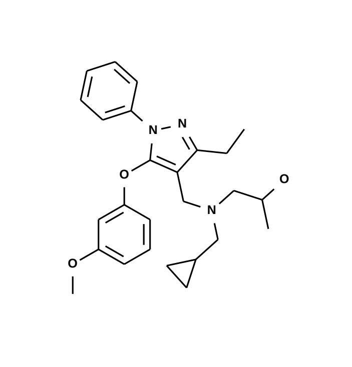 | 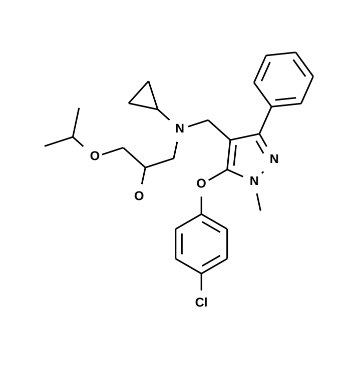 |
| ALB-H04279679 | ALB-H04290857 | ALB-H04311305 | ALB-H04312401 | ALB-H04317289 | ALB-H04318609 |
| 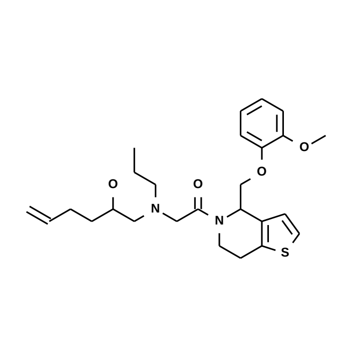 | 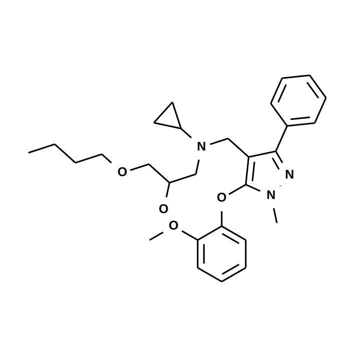 | 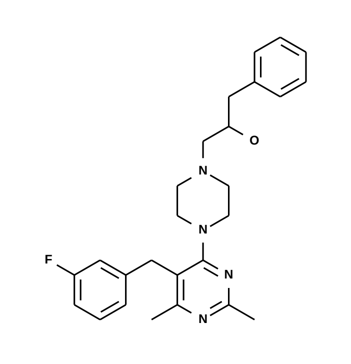 | 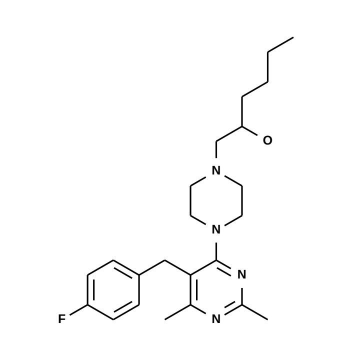 | 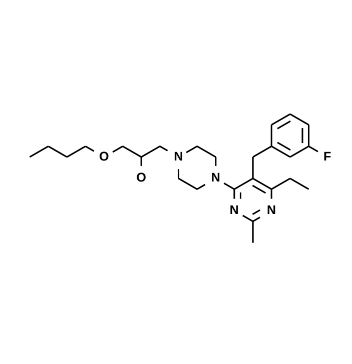 | 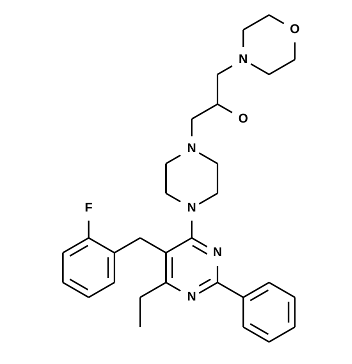 |

**ALB-H04318784**

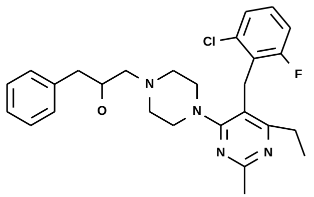

**ALB-H04323998**

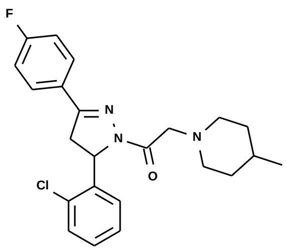

**ALB-H04332437**

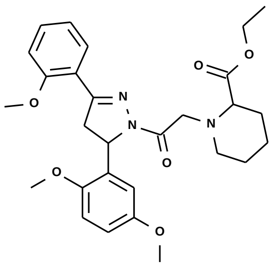

**ALB-H04335442**

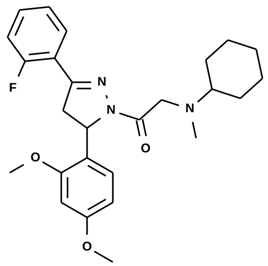

**ALB-H04344946**

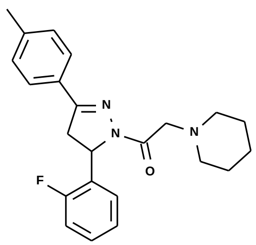

**ALB-H04345135**

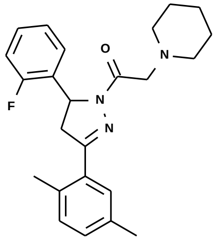

**ALB-H04347021**

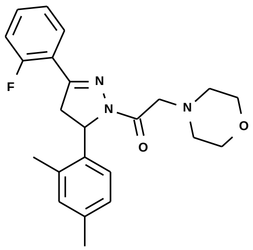

**ALB-H04349347**

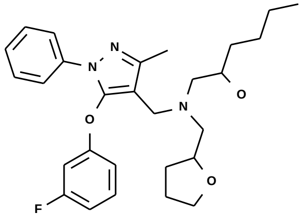

**ALB-H04350886**

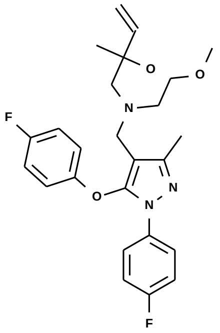

**ALB-H04352561**

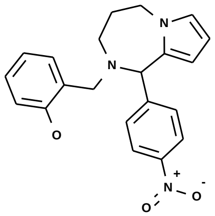

**ALB-H04352577**

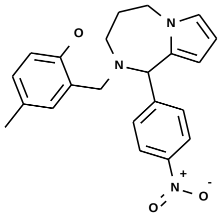

**ALB-H04353431**

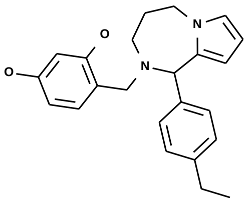

**ALB-H04354534**

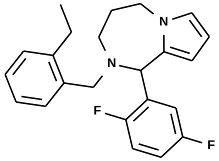

**ALB-H04491222**

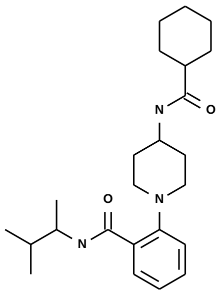

**ALB-H09014869**

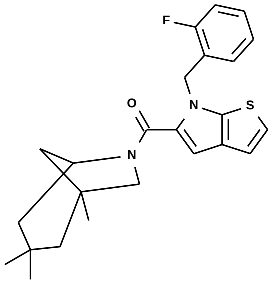

**ALB-H09027575**

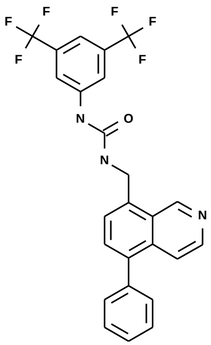

**ALB-H09027712**

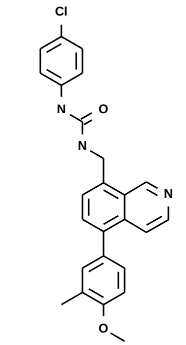

**ALB-H09027731**

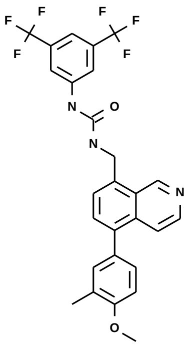

**ALB-H09033306**

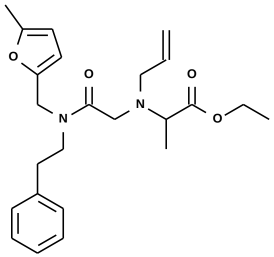

**ALB-H09062143**

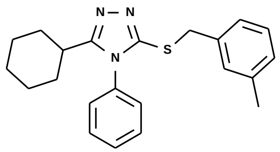

**ALB-H09063304**

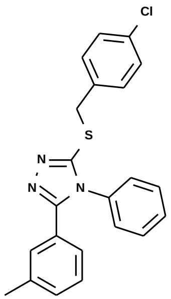

**ALB-H09063329**

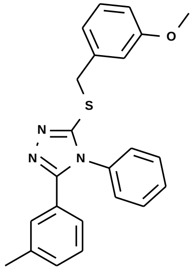

**ALB-H09069453**

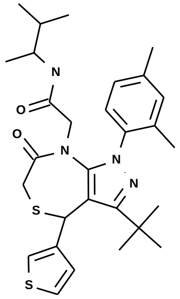

**ALB-H09252326**

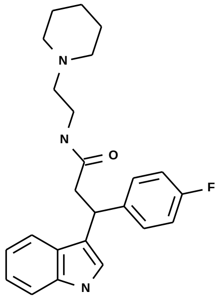

| ALB-H09252492 | ALB-H09252761 | ALB-H09254856 | ALB-H09449960 | ALB-H09450316 | ALB-H09450534 |
|---|---|---|---|---|---|
| 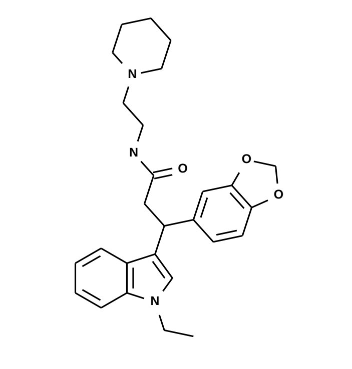 | 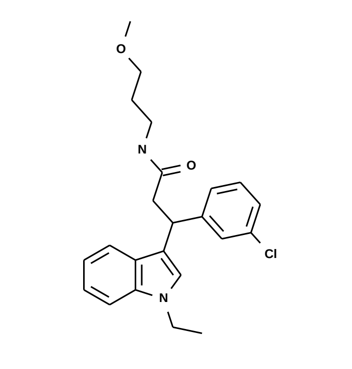 | 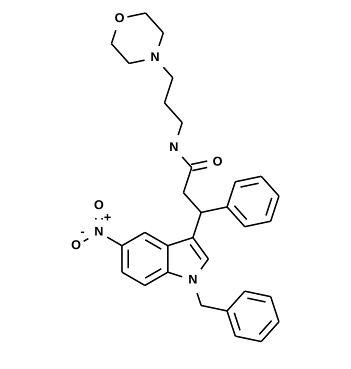 | 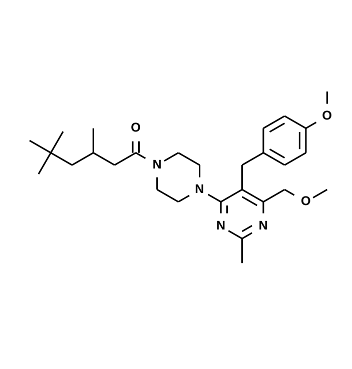 | 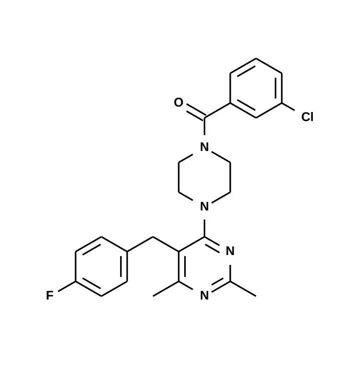 | 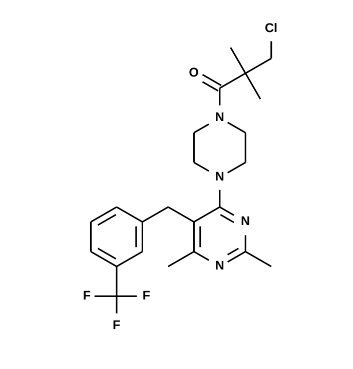 |
| ALB-H09453634 | ALB-H09454509 | ALB-H09454592 | ALB-H09460060 | ALB-H09469411 | ALB-H09469422 |
| 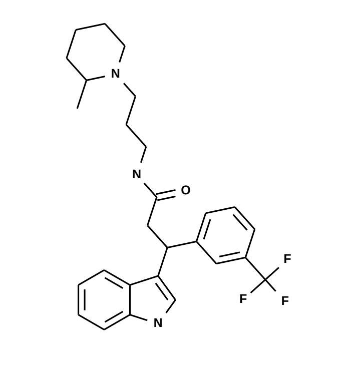 | 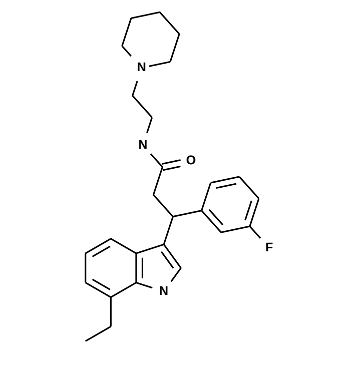 | 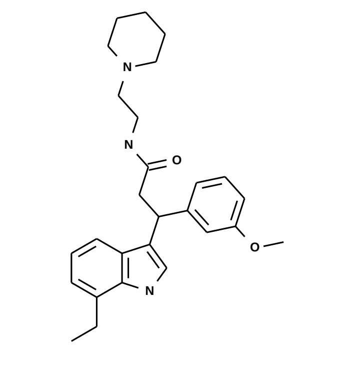 | 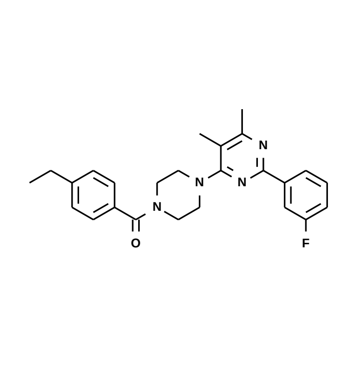 | 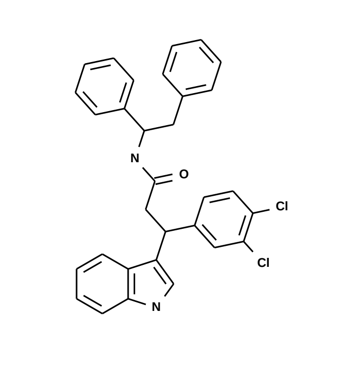 | 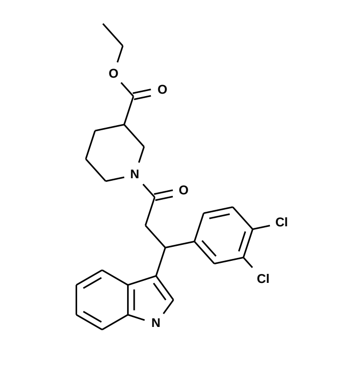 |
| ALB-H09469542 | ALB-H09474247 | ALB-H09485136 | ALB-H09490245 | ALB-H09490273 | ALB-H09490565 |
| 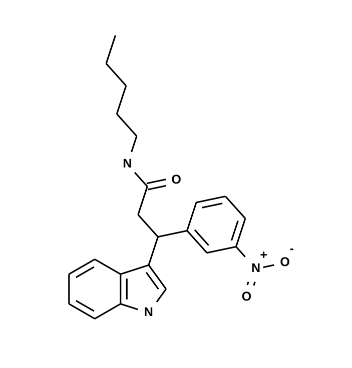 | 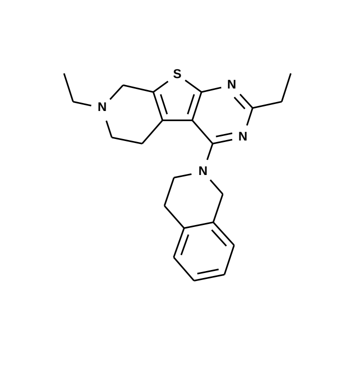 | 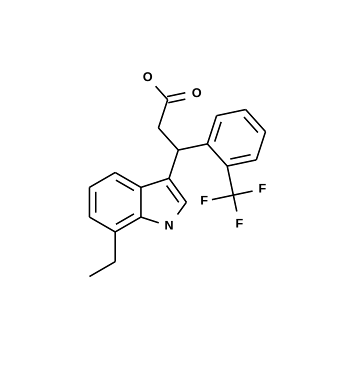 | 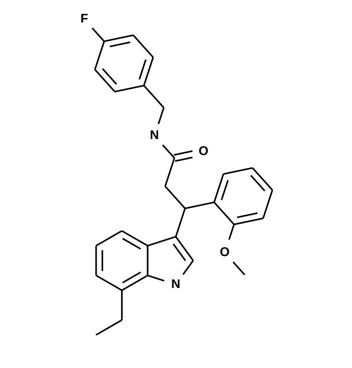 | 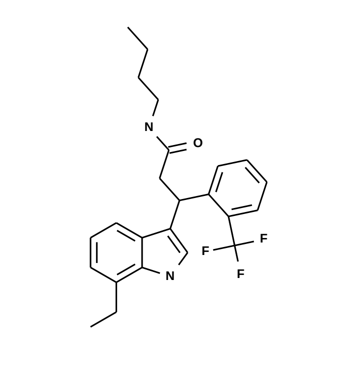 | 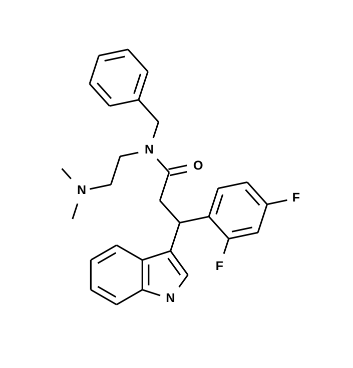 |
| ALB-H09500235 | ALB-H09500554 | ALB-H09507055 | ALB-H09509075 | ALB-H09509214 | ALB-H09509386 |
| 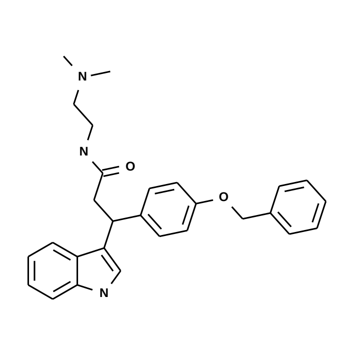 | 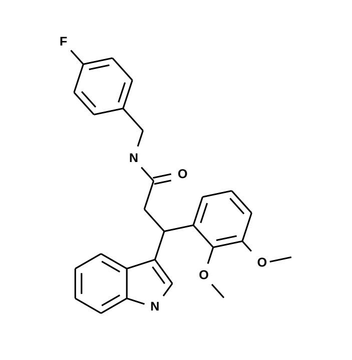 | 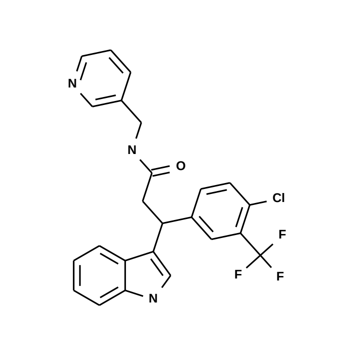 | 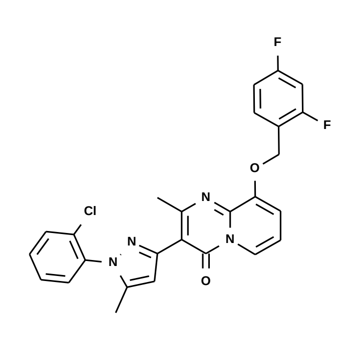 | 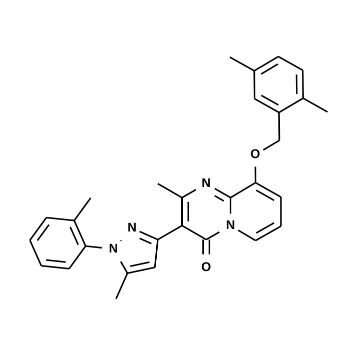 | 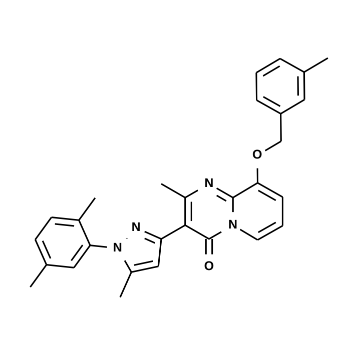 |

**ALB-H09511573**

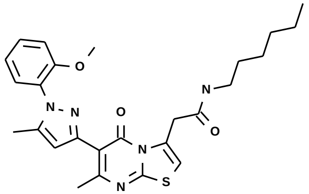

**ALB-H09545669**

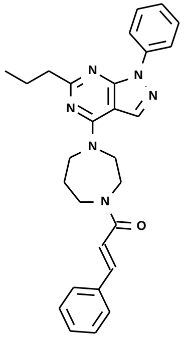

**ALB-H09546357**

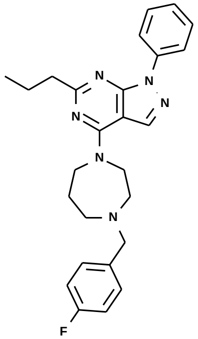

**ALB-H09652704**

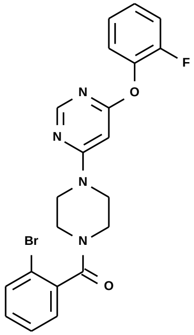

**ALB-H09733014**

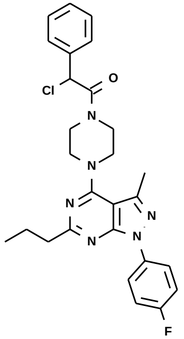

**ALB-H09828397**

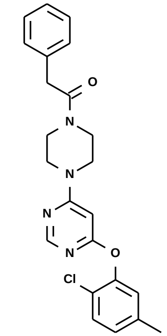

**ALB-H09978268**

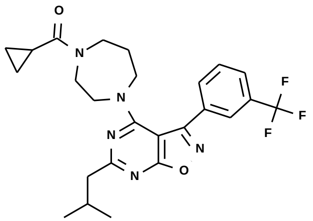

**ALB-H09988469**

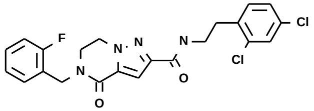

**ALB-H09989501**

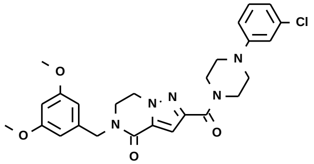

**ALB-H09990625**

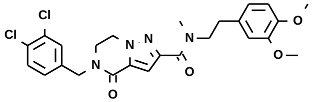

**ALB-H10053119**

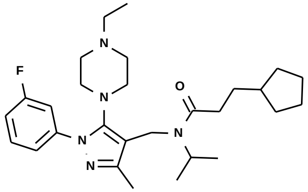

**ALB-H10109623**

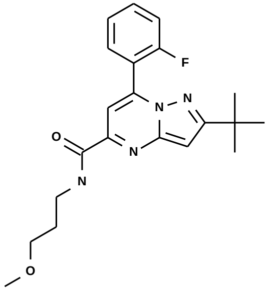

**ALB-H10119147**

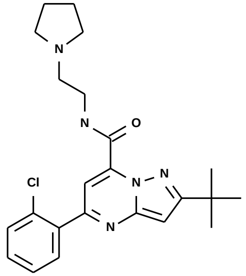

**ALB-H10119487**

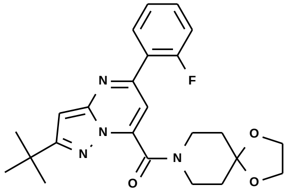

**ALB-H10293904**

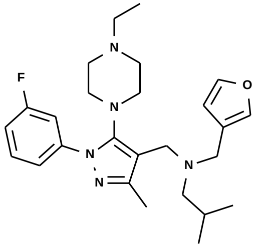

**ALB-H10705246**

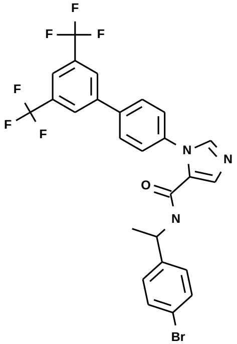

**ALB-H10705303**

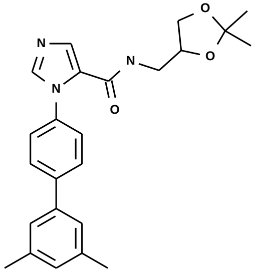

**ALB-H10705704**

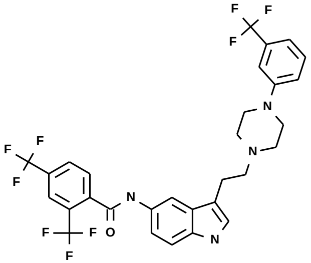

**ALB-H10710816**

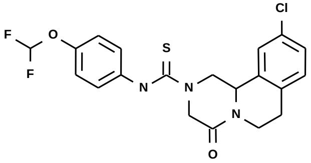

**ALB-H10713342**

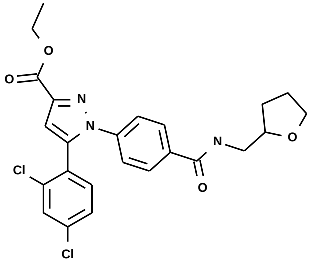

**ALB-H10716881**

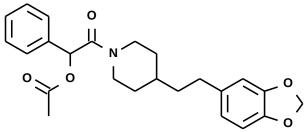

**ALB-H10719209**

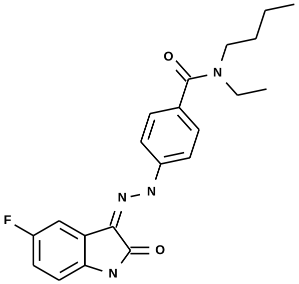

**ALB-H10720953**

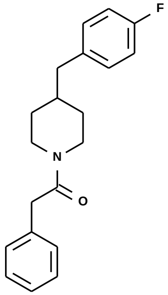

**ALB-H10721017**

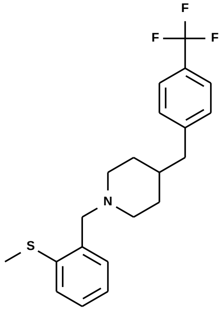

**ALB-H10721531**

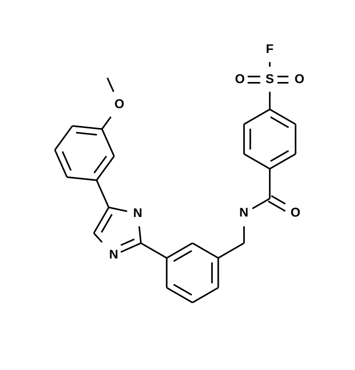

**ALB-H10721849**

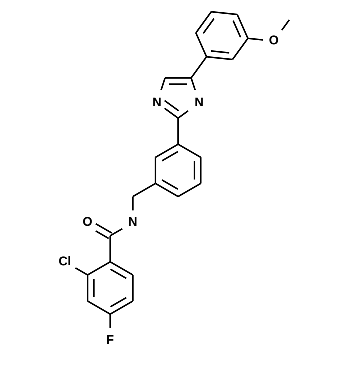

**ALB-H10722143**

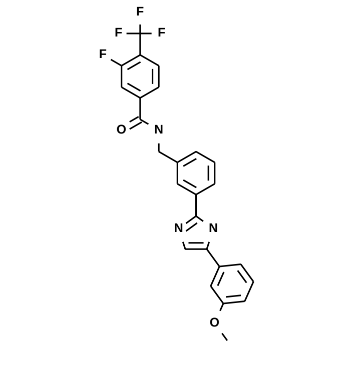

**ALB-H10725278**

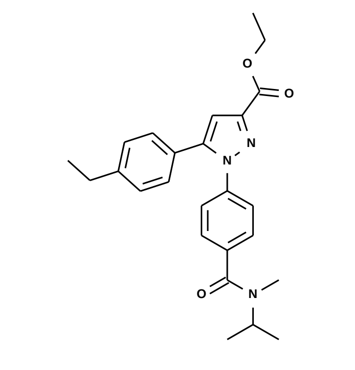

**ALB-H10726123**

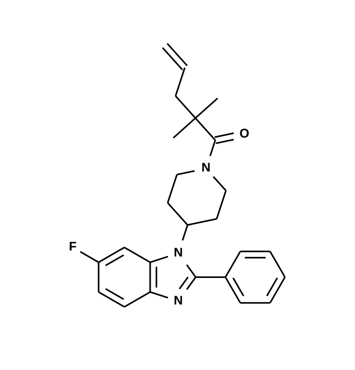

**ALB-H10726214**

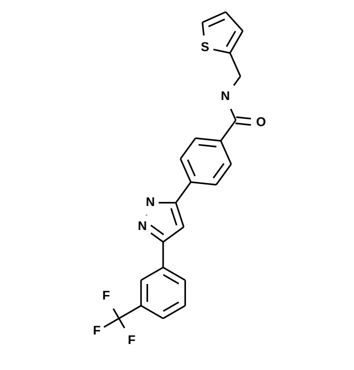

**ALB-H10726606**

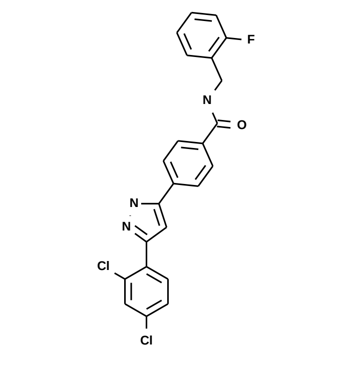

**ALB-H10726614**

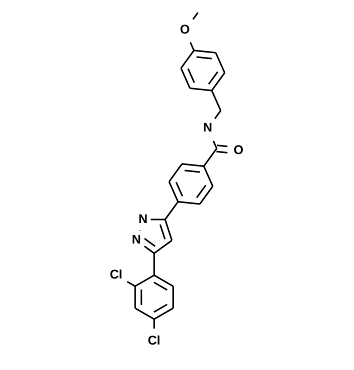

**ALB-H10726913**

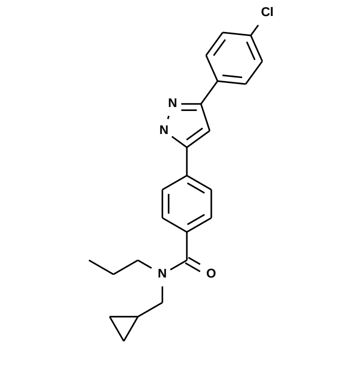

**ALB-H10727038**

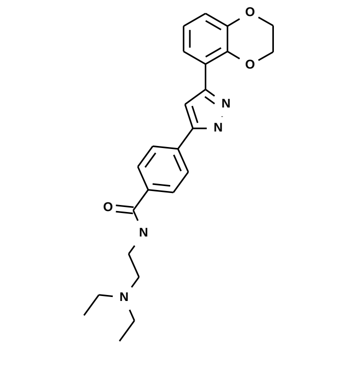

**ALB-H10727084**

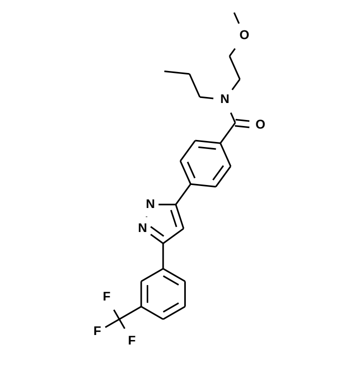

**ALB-H10727259**

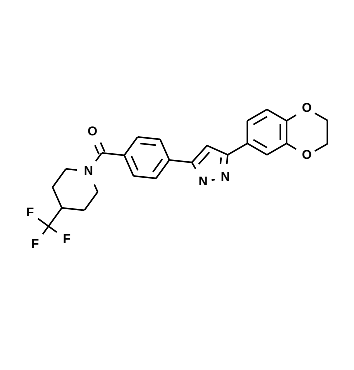

**ALB-H10728280**

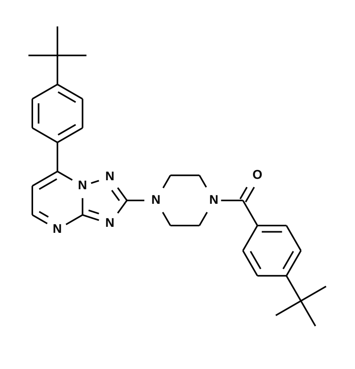

**ALB-H10728351**

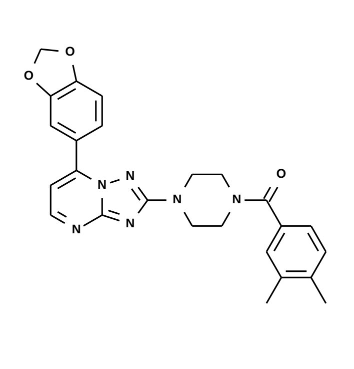

**ALB-H10731349**

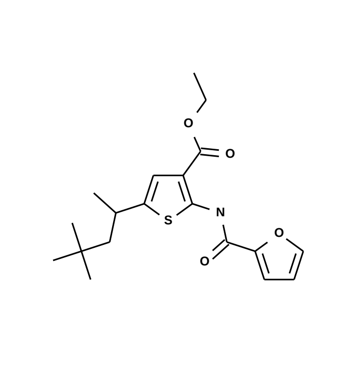

**ALB-H10732494**

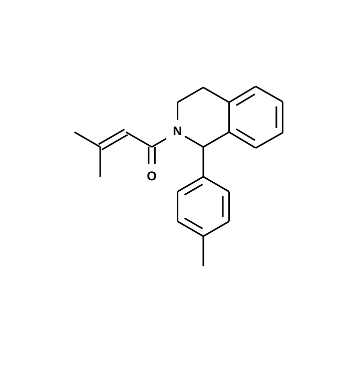

**ALB-H10732581**

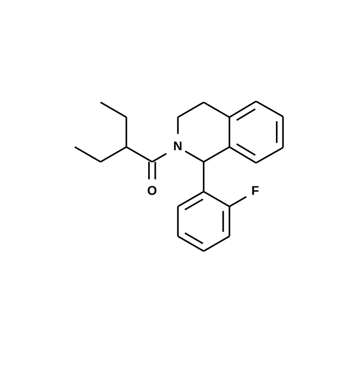

**ALB-H10732606**

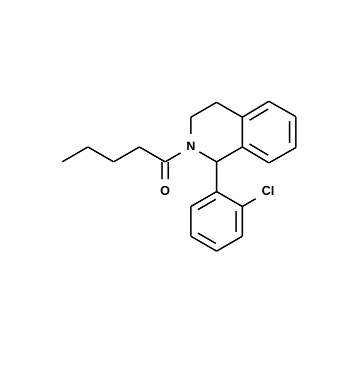

**ALB-H10732615**

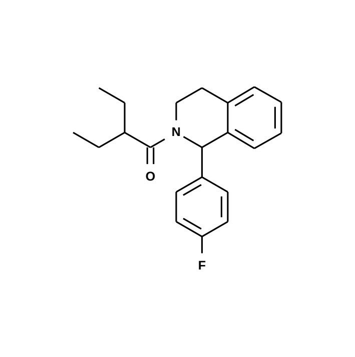

**ALB-H10733662**

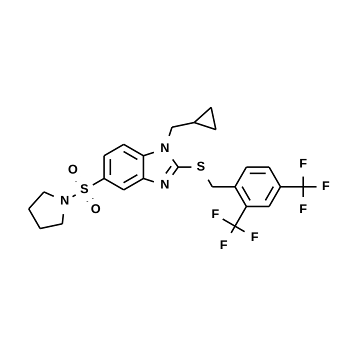

**ALB-H10736021**

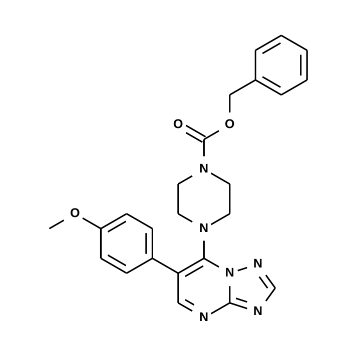

**ALB-H10737041**

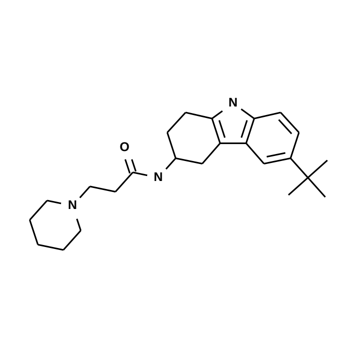

**ALB-H10737303**

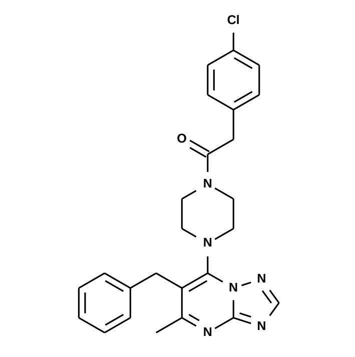

**ALB-H10740083**

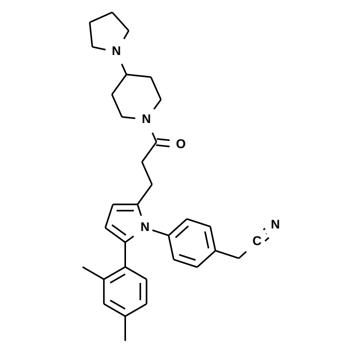

| ALB-H10740612 | ALB-H10742649 | ALB-H10743190 | ALB-H10744061 | ALB-H10744131 | ALB-H10745264 |
|---|---|---|---|---|---|
| 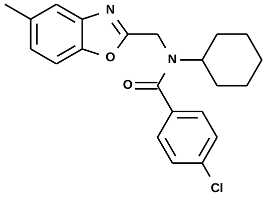 | 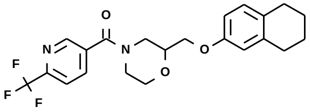 | 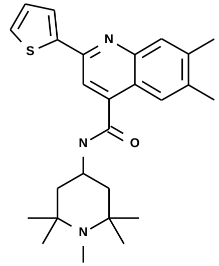 | 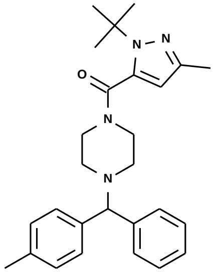 | 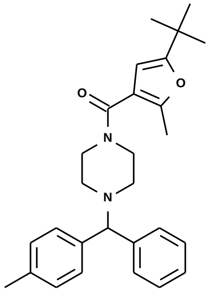 | 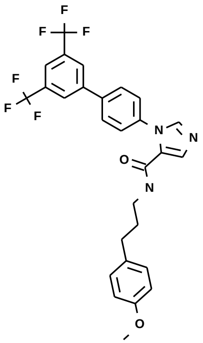 |
| **ALB-H10746586** | **ALB-H10747405** | **ALB-H10749428** | **ALB-H10787372** | **ALB-H10845002** | |
| 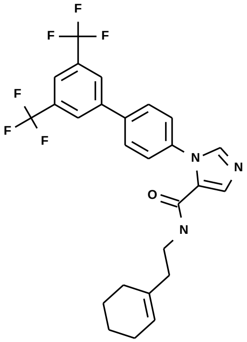 | 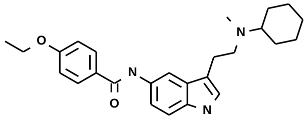 | 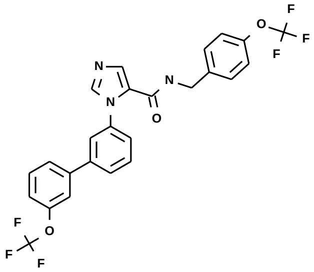 | 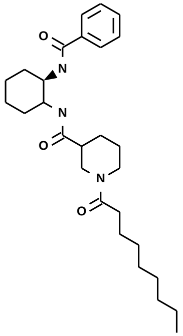 | 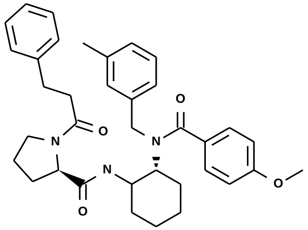 | |
