# Supplementary material for: A Chemocentric Approach to the Identification of Cancer Targets
Source: PLoS One. 2012 Apr 25;7(4):e35582. doi: 10.1371/journal.pone.0035582 (PMC3338416; doi:10.1371/journal.pone.0035582)
Supplement: Table S1 — List of all 115 proteins identified by the DIVISS approach as from small molecule hits selective to HCT116 relative to MRC-5. The OncoScore is the oncogene probability calculated with CGPrio [34]. The arrows next to the gene name mark the set of 29 proteins that are known to be significantly altered (corrected p-value <0.05) in terms of up- or down-regulation in colon cancer, as extracted from the IntOGen platform [33]. (DOC) [file pone.0035582.s006.doc]

**Table S1. List of all 115 proteins identified by the DIVISS approach as from small molecule hits selective to HCT116 relative to MRC-5. The OncoScore is the oncogene probability calculated with CGPrio [Nucl Acids Res 2008;36:e115]. The arrows next to the gene name mark the set of 29 proteins that are known to be significantly altered (corrected p-value < 0.05) in terms of up- or down-regulation in colon cancer, as extracted from the IntOGen platform [Nat Methods 2010;7:92-93].**

| **No.** | **Protein Name** | **Gene Name** | **Uniprot** | **Function** | **OncoScore** |
| --- | --- | --- | --- | --- | --- |
| 1 | Proto-oncogene tyrosine-protein kinase ABL1 | ABL1 | P00519 | EC - Transferase | 1.000 |
| 2 | Alpha-2Da adrenergic receptor | ADRA2DA | Q8JG70 | G Protein-Coupled Receptor | NA |
| 3 | Arachidonate 5-lipoxygenase | ALOX5 **↑** | P09917 | EC - Oxidoreductase | 0.031 |
| 4 | Apelin receptor | APLNR | P35414 | G Protein-Coupled Receptor | 0.195 |
| 5 | Androgen receptor | AR | P10275 | Transcription Factor | 1.000 |
| 6 | Potassium-transporting ATPase alpha chain 1 | ATP4A | P20648 | EC - Hydrolase | 0.078 |
| 7 | Potassium-transporting ATPase subunit beta | ATP4B | P51164 | Other | 0.004 |
| 8 | Serine/threonine-protein kinase 12 | AURKB | Q96GD4 | EC - Transferase | 0.003 |
| 9 | B-Raf proto-oncogene serine/threonine-protein kinase | BRAF | P15056 | EC - Transferase | 1.000 |
| 10 | Carbonic anhydrase 7 | CA7 **↓** | P43166 | EC - Other | 0.191 |
| 11 | Carbonic anhydrase 9 | CA9 **↑** | Q16790 | EC - Other | 0.005 |
| 12 | C-C chemokine receptor type 3 | CCR3 | P51677 | G Protein-Coupled Receptor | 0.021 |
| 13 | Cell division protein kinase 2 | CDK2 | P24941 | EC - Transferase | 0.999 |
| 14 | Cell division protein kinase 5 | CDK5 | Q00535 | EC - Transferase | 0.998 |
| 15 | Cyclin-dependent kinase 5 activator 1 | CDK5R1 | Q15078 | Transcription Factor | 0.701 |
| 16 | Cystic fibrosis transmembrane conductance regulator | CFTR **↓** | P13569 | Ion Channel | 0.463 |
| 17 | Neuronal acetylcholine receptor | CHRNA4 | P43681 | Ion Channel | 0.056 |
| 18 | Neuronal acetylcholine receptor subunit alpha-7 | CHRNA7 | P36544 | Ion Channel | 0.351 |
| 19 | Dual specificity protein kinase CLK4 | CLK4 | Q9HAZ1 | EC - Transferase | 0.120 |
| 20 | Corticotropin-releasing factor receptor 2 | CRHR2 | Q13324 | G Protein-Coupled Receptor | 0.070 |
| 21 | Beta-casein | CSN2 | P05814 | Other | 0.021 |
| 22 | Cytochrome P450 1A2 | CYP1A2 | P05177 | EC - Oxidoreductase | 0.065 |
| 23 | Cytochrome P450 2C9 | CYP2C9 **↓** | P11712 | EC - Oxidoreductase | 0.101 |
| 24 | Cytochrome P450 3A4 | CYP3A4 **↓** | P08684 | EC - Oxidoreductase | 0.099 |
| 25 | Lanosterol 14-alpha demethylase | CYP51A1 **↑** | Q16850 | EC - Oxidoreductase | 0.075 |
| 26 | Dihydroorotate dehydrogenase, mitochondrial | DHODH | Q02127 | EC - Oxidoreductase | 0.017 |
| 27 | D(1A) dopamine receptor | DRD1 | P21728 | G Protein-Coupled Receptor | 0.866 |
| 28 | D(1B) dopamine receptor | DRD5 | P21918 | G Protein-Coupled Receptor | 0.040 |
| 29 | 3-beta-hydroxysteroid-Delta(8),Delta(7)-isomerase | EBP | Q15125 | EC - Other | 0.001 |
| 30 | Endothelin-converting enzyme 1 | ECE1 | P42892 | EC - Hydrolase | 0.039 |
| 31 | Epidermal growth factor receptor | EGFR | P00533 | EC - Transferase | 1.000 |
| 32 | Epoxide hydrolase 2 | EPHX2 **↓** | P34913 | EC - Hydrolase | 0.003 |
| 33 | Estrogen receptor | ESR1 | P03372 | Transcription Factor | 1.000 |
| 34 | Estrogen receptor beta | ESR2 | Q92731 | Transcription Factor | 0.998 |
| 35 | Prothrombin | F2 | P00734 | EC - Hydrolase | 0.006 |
| 36 | 3-oxoacyl-[acyl-carrier-protein] synthase 3 | FABH | P0A6R0 | EC - Transferase | NA |
| 37 | Fructose-1,6-bisphosphatase isozyme 2 | FBP2 | O00757 | EC - Hydrolase | 0.006 |
| 38 | Proto-oncogene tyrosine-protein kinase FGR | FGR | P09769 | EC - Transferase | 0.992 |
| 39 | Peptidyl-prolyl cis-trans isomerase FKBP1A | FKBP1A **↓** | P62942 | EC - Other | 0.999 |
| 40 | Vascular endothelial growth factor receptor 1 | FLT1 | P17948 | EC - Transferase | 1.000 |
| 41 | FL cytokine receptor | FLT3 | P36888 | EC - Transferase | 1.000 |
| 42 | Vascular endothelial growth factor receptor 3 | FLT4 | P35916 | EC - Transferase | 1.000 |
| 43 | Glutamate [NMDA] receptor subunit zeta-1 | GRIN1 | Q05586 | Ion Channel | 0.521 |
| 44 | Metabotropic glutamate receptor 2 | GRM2 | Q14416 | G Protein-Coupled Receptor | 0.032 |
| 45 | Glycogen synthase kinase-3 alpha | GSK3A | P49840 | EC - Transferase | 0.996 |
| 46 | Histone deacetylase 1 | HDAC1 | Q13547 | EC - Hydrolase | 0.895 |
| 47 | Histone deacetylase 10 | HDAC10 | Q969S8 | EC - Hydrolase | 0.199 |
| 48 | Histone deacetylase 11 | HDAC11 | Q96DB2 | EC - Hydrolase | 0.019 |
| 49 | Histone deacetylase 2 | HDAC2 | Q92769 | EC - Hydrolase | 0.119 |
| 50 | Histone deacetylase 3 | HDAC3 | O15379 | EC - Hydrolase | 0.193 |
| 51 | Histone deacetylase 4 | HDAC4 | P56524 | EC - Hydrolase | 0.975 |
| 52 | Histone deacetylase 5 | HDAC5 | Q9UQL6 | EC - Hydrolase | NA |
| 53 | Histone deacetylase 6 | HDAC6 | Q9UBN7 | EC - Hydrolase | 0.895 |
| 54 | Histone deacetylase 7 | HDAC7 | Q8WUI4 | EC - Hydrolase | 0.959 |
| 55 | Histone deacetylase 8 | HDAC8 **↑** | Q9BY41 | EC - Hydrolase | 0.373 |
| 56 | Histone deacetylase 9 | HDAC9 **↓** | Q9UKV0 | EC - Hydrolase | 0.953 |
| 57 | Histamine H3 receptor | HRH3 | Q9Y5N1 | G Protein-Coupled Receptor | NA |
| 58 | Corticosteroid 11-beta-dehydrogenase isozyme 2 | HSD11B2 **↓** | P80365 | EC - Oxidoreductase | 0.011 |
| 59 | Heat shock protein HSP 90-alpha | HSP90AA1 | P07900 | Other | 0.023 |
| 60 | 5-hydroxytryptamine receptor 5A | HTR5A | P47898 | G Protein-Coupled Receptor | 0.152 |
| 61 | Potassium voltage-gated channel subfamily KQT member 1 | KCNQ1 **↑** | P51787 | Ion Channel | 0.192 |
| 62 | Potassium voltage-gated channel subfamily KQT member 2 | KCNQ2 | O43526 | Ion Channel | 0.655 |
| 63 | Vascular endothelial growth factor receptor 2 | KDR | P35968 | EC - Transferase | NA |
| 64 | Mast/stem cell growth factor receptor | KIT **↓** | P10721 | EC - Transferase | 1.000 |
| 65 | Krueppel-like factor 5 | KLF5 **↓** | Q13887 | Transcription Factor | 0.745 |
| 66 | Lanosterol synthase | LSS | P48449 | EC - Other | 0.009 |
| 67 | Leukotriene A-4 hydrolase | LTA4H | P09960 | EC - Hydrolase | 0.040 |
| 68 | Amine oxidase [flavin-containing] A | MAOA **↓** | P21397 | EC - Oxidoreductase | 0.427 |
| 69 | Amine oxidase [flavin-containing] B | MAOB **↓** | P27338 | EC - Oxidoreductase | 0.513 |
| 70 | Dual specificity mitogen-activated protein kinase kinase 1 | MAP2K1 | Q02750 | EC - Transferase | 0.895 |
| 71 | Mitogen-activated protein kinase kinase kinase 8 | MAP3K8 **↑** | P41279 | EC - Transferase | 0.984 |
| 72 | Mitogen-activated protein kinase 10 | MAPK10 | P53779 | EC - Transferase | 0.974 |
| 73 | Mitogen-activated protein kinase 8 | MAPK8 | P45983 | EC - Transferase | 0.665 |
| 74 | Induced myeloid leukemia cell differentiation protein Mcl-1 | MCL1 | Q07820 | Other | 0.118 |
| 75 | E3 ubiquitin-protein ligase Mdm2 | MDM2 **↑** | Q00987 | EC - Other | 0.966 |
| 76 | Hepatocyte growth factor receptor | MET **↑** | P08581 | EC - Transferase | 1.000 |
| 77 | Monoglyceride lipase | MGLL | Q99685 | EC - Hydrolase | 0.426 |
| 78 | NADH-ubiquinone oxidoreductase | MT-ND1 | P03886 | EC - Oxidoreductase | NA |
| 79 | Nuclear factor of activated T-cells, cytoplasmic 1 | NFATC1 | O95644 | Transcription Factor | 0.999 |
| 80 | Nuclear factor of activated T-cells, cytoplasmic 2 | NFATC2 | Q13469 | Transcription Factor | 0.567 |
| 81 | Nitric oxide synthase, inducible | NOS2 **↑** | P35228 | EC - Oxidoreductase | 0.250 |
| 82 | Neuropeptide Y receptor type 5 | NPY5R | Q15761 | G Protein-Coupled Receptor | 0.004 |
| 83 | Bile acid receptor | NR1H4 **↓** | Q96RI1 | Transcription Factor | 0.366 |
| 84 | Steroidogenic factor 1 | NR5A1 | Q13285 | Transcription Factor | 0.100 |
| 85 | Poly [ADP-ribose] polymerase 1 | PARP1 | P09874 | EC - Transferase | 0.711 |
| 86 | Phosphodiesterase isozyme 5 | PDE5 | Q864F1 | Other | NA |
| 87 | Alpha-type platelet-derived growth factor receptor | PDGFRA **↓** | P16234 | EC - Transferase | 1.000 |
| 88 | Phosphatidylinositol-4,5-bisphosphate 3-kinase | PIK3CD | O00329 | EC - Transferase | 0.708 |
| 89 | Serine/threonine-protein kinase Pim-3 | PIM3 | Q86V86 | EC - Transferase | NA |
| 90 | Peroxisome proliferator-activated receptor gamma | PPARG | P37231 | Transcription Factor | 0.959 |
| 91 | Peptidyl-prolyl cis-trans isomerase A | PPIA **↑** | P62937 | EC - Other | NA |
| 92 | Prostaglandin D2 receptor | PTGDR **↓** | Q13258 | G Protein-Coupled Receptor | 0.030 |
| 93 | Prostaglandin E2 receptor EP2 subtype | PTGER2 | P43116 | G Protein-Coupled Receptor | 0.130 |
| 94 | Prostaglandin E2 receptor EP3 subtype | PTGER3 | P43115 | G Protein-Coupled Receptor | 0.029 |
| 95 | Prostaglandin E2 receptor EP4 subtype | PTGER4 **↓** | P35408 | G Protein-Coupled Receptor | 0.286 |
| 96 | RAF proto-oncogene serine/threonine-protein kinase | RAF1 | P04049 | EC - Transferase | 1.000 |
| 97 | Protein recA | RECA | P0A7G6 | Other | NA |
| 98 | C-Rel proto-oncogene protein | REL | Q04864 | Transcription Factor | NA |
| 99 | Sphingosine 1-phosphate receptor 1 | S1PR1 | P21453 | G Protein-Coupled Receptor | 0.863 |
| 100 | Acyl-CoA desaturase | SCD **↑** | O00767 | EC - Oxidoreductase | 0.940 |
| 101 | E-selectin | SELE **↑** | P16581 | Other | 0.027 |
| 102 | Streptokinase A | SKA | P10520 | Other | NA |
| 103 | Sodium-dependent noradrenaline transporter | SLC6A2 | P23975 | Other | NA |
| 104 | Proto-oncogene tyrosine-protein kinase Src | SRC **↑** | P12931 | EC - Transferase | 1.000 |
| 105 | 3-oxo-5-alpha-steroid 4-dehydrogenase 2 | SRD5A2 | P31213 | EC - Oxidoreductase | 0.045 |
| 106 | Somatostatin receptor type 5 | SSTR5 | P35346 | G Protein-Coupled Receptor | NA |
| 107 | Signal transducer and activator of transcription 1-alpha/beta | STAT1 | P42224 | Transcription Factor | 0.824 |
| 108 | Signal transducer and activator of transcription 3 | STAT3 | P40763 | Transcription Factor | 0.999 |
| 109 | Neuromedin-K receptor (NK3) | TACR3 | P29371 | G Protein-Coupled Receptor | 0.339 |
| 110 | Thromboxane A2 receptor | TBXA2R | P21731 | G Protein-Coupled Receptor | NA |
| 111 | Angiopoietin-1 receptor | TEK | Q02763 | EC - Transferase | 1.000 |
| 112 | TGF-beta receptor type-1 | TGFBR1 | P36897 | EC - Transferase | 0.970 |
| 113 | Short transient receptor potential channel 4 | TRPC4 | Q9UBN4 | Ion Channel | 0.981 |
| 114 | Tubulin beta chain | TUBB | P07437 | Other | NA |
| 115 | Protease precursor | UL26 | P10210 | EC - Hydrolase | NA |
