# Supplementary material for: A Chemocentric Approach to the Identification of Cancer Targets
Source: PLoS One. 2012 Apr 25;7(4):e35582. doi: 10.1371/journal.pone.0035582 (PMC3338416; doi:10.1371/journal.pone.0035582)
Supplement: Table S2 — List of all interactions available in PubChem for compounds present within the list of 265 cytotoxic selective in HCT116 cell lines. (DOC) [file pone.0035582.s007.doc]

**Table S2.** List of all interactions available in PubChem for compounds present within the list of 265 cytotoxic selective in HCT116 cell lines.

| **PubChem CID** | **PubChem AID** | **Pubchem_BioAssay_name** |
| --- | --- | --- |
| 12265205 | 477796 | Inhibition of amyloid beta (1 to 40) aggregation |
| 10403008 | 126687 | Ability to inhibit Monoamine oxidase B enzyme |
| 10403008 | 273203 | Inhibition of human supersomes MAOB |
| 10403008 | 273204 | Inhibition of rat brain MAOB |
| 11776102 | 126687 | Ability to inhibit Monoamine oxidase B enzyme |
| 6409776 | 126687 | Ability to inhibit Monoamine oxidase B enzyme |
| 6409776 | 273203 | Inhibition of human supersomes MAOB |
| 6409776 | 273204 | Inhibition of rat brain MAOB |
| 10378713 | 126687 | Ability to inhibit Monoamine oxidase B enzyme |
| 10378713 | 273203 | Inhibition of human supersomes MAOB |
| 10378713 | 273204 | Inhibition of rat brain MAOB |
| 3347358 | 477796 | Inhibition of amyloid beta (1 to 40) aggregation |
| 314791 | 248 | Data for tumor model L1210 Leukemia (intraperitoneal) in B6D2F1 (BDF1) mice |
| 387030 | 1 | Data for the NCI-H23 Non-Small Cell Lung cell line [Confirmatory] |
| 387030 | 3 | Data for the NCI-H226 Non-Small Cell Lung cell line [Confirmatory] |
| 387030 | 5 | Data for the NCI-H322M Non-Small Cell Lung cell line [Confirmatory] |
| 387030 | 7 | Data for the NCI-H460 Non-Small Cell Lung cell line [Confirmatory] |
| 387030 | 13 | Data for the HOP-92 Non-Small Cell Lung cell line [Confirmatory] |
| 387030 | 15 | Data for the NCI-H522 Non-Small Cell Lung cell line [Confirmatory] |
| 387030 | 19 | Data for the A549/ATCC Non-Small Cell Lung cell line [Confirmatory] |
| 387030 | 21 | Data for the EKVX Non-Small Cell Lung cell line [Confirmatory] |
| 387030 | 25 | Data for the M14 Melanoma cell line [Confirmatory] |
| 387030 | 29 | Data for the MALME-3M Melanoma cell line [Confirmatory] |
| 387030 | 31 | Data for the UACC-62 Melanoma cell line [Confirmatory] |
| 387030 | 33 | Data for the UACC-257 Melanoma cell line [Confirmatory] |
| 387030 | 35 | Data for the SK-MEL-2 Melanoma cell line [Confirmatory] |
| 387030 | 37 | Data for the SK-MEL-5 Melanoma cell line [Confirmatory] |
| 387030 | 41 | Data for the PC-3 Prostate cell line [Confirmatory] |
| 387030 | 43 | Data for the DU-145 Prostate cell line [Confirmatory] |
| 387030 | 45 | Data for the SF-268 Central Nervous System cell line [Confirmatory] |
| 387030 | 47 | Data for the SF-295 Central Nervous System cell line [Confirmatory] |
| 387030 | 49 | Data for the SF-539 Central Nervous System cell line [Confirmatory] |
| 387030 | 53 | Data for the SNB-19 Central Nervous System cell line [Confirmatory] |
| 387030 | 55 | Data for the SNB-75 Central Nervous System cell line [Confirmatory] |
| 387030 | 59 | Data for the U251 Central Nervous System cell line [Confirmatory] |
| 387030 | 65 | Data for the HT29 Colon cell line [Confirmatory] |
| 387030 | 67 | Data for the COLO 205 Colon cell line [Confirmatory] |
| 387030 | 71 | Data for the HCT-15 Colon cell line [Confirmatory] |
| 387030 | 73 | Data for the KM12 Colon cell line [Confirmatory] |
| 387030 | 79 | Data for the HCT-116 Colon cell line [Confirmatory] |
| 387030 | 81 | Data for the SW-620 Colon cell line [Confirmatory] |
| 387030 | 83 | Data for the MCF7 Breast cell line [Confirmatory] |
| 387030 | 85 | Data for the MDA-MB-435 Breast cell line [Confirmatory] |
| 387030 | 87 | Data for the MDA-N Breast cell line [Confirmatory] |
| 387030 | 89 | Data for the BT-549 Breast cell line [Confirmatory] |
| 387030 | 91 | Data for the T-47D Breast cell line [Confirmatory] |
| 387030 | 93 | Data for the NCI/ADR-RES Breast cell line [Confirmatory] |
| 387030 | 95 | Data for the MDA-MB-231/ATCC Breast cell line [Confirmatory] |
| 387030 | 97 | Data for the HS 578T Breast cell line [Confirmatory] |
| 387030 | 101 | Data for the IGROV1 Ovarian cell line [Confirmatory] |
| 387030 | 103 | Data for the SK-OV-3 Ovarian cell line [Confirmatory] |
| 387030 | 105 | Data for the OVCAR-4 Ovarian cell line [Confirmatory] |
| 387030 | 107 | Data for the OVCAR-5 Ovarian cell line [Confirmatory] |
| 387030 | 109 | Data for the OVCAR-8 Ovarian cell line [Confirmatory] |
| 387030 | 113 | Data for the RPMI-8226 Leukemia cell line [Confirmatory] |
| 387030 | 115 | Data for the SR Leukemia cell line [Confirmatory] |
| 387030 | 119 | Data for the CCRF-CEM Leukemia cell line [Confirmatory] |
| 387030 | 121 | Data for the K-562 Leukemia cell line [Confirmatory] |
| 387030 | 123 | Data for the MOLT-4 Leukemia cell line [Confirmatory] |
| 387030 | 125 | Data for the HL-60(TB) Leukemia cell line [Confirmatory] |
| 387030 | 131 | Data for the CAKI-1 Renal cell line [Confirmatory] |
| 387030 | 133 | Data for the RXF 393 Renal cell line [Confirmatory] |
| 387030 | 137 | Data for the 786-0 Renal cell line [Confirmatory] |
| 387030 | 139 | Data for the ACHN Renal cell line [Confirmatory] |
| 387030 | 141 | Data for the TK-10 Renal cell line [Confirmatory] |
| 387030 | 143 | Data for the UO-31 Renal cell line [Confirmatory] |
| 387030 | 145 | Data for the SN12C Renal cell line [Confirmatory] |
| 387030 | 179 | NCI AIDS Antiviral Assay [Confirmatory] |
| 387030 | 302239 | Inhibition of MAOB in rat brain mitochondria |
| 387030 | 302240 | Inhibition of MAOA in rat brain mitochondria at 10 uM |
